# Identifying the Basal Ganglia Network Model Markers for Medication-Induced Impulsivity in Parkinson's Disease Patients

**DOI:** 10.1371/journal.pone.0127542

**Published:** 2015-06-04

**Authors:** Pragathi Priyadharsini Balasubramani, V. Srinivasa Chakravarthy, Manal Ali, Balaraman Ravindran, Ahmed A. Moustafa

**Affiliations:** 1 Department of Biotechnology, Indian Institute of Technology, Madras, Chennai, India; 2 School of Medicine, Ain Shams University, Cairo, Egypt; 3 Department of Computer Science and Engineering, Indian Institute of Technology, Madras, Chennai, India; 4 Marcs Institute for Brain and Behaviour & School of Social Sciences and Psychology, University of Western Sydney, Penrith, Australia; Mayo Clinic, UNITED STATES

## Abstract

Impulsivity, i.e. irresistibility in the execution of actions, may be prominent in Parkinson's disease (PD) patients who are treated with dopamine precursors or dopamine receptor agonists. In this study, we combine clinical investigations with computational modeling to explore whether impulsivity in PD patients on medication may arise as a result of abnormalities in risk, reward and punishment learning. In order to empirically assess learning outcomes involving risk, reward and punishment, four subject groups were examined: healthy controls, ON medication PD patients with impulse control disorder (PD-ON ICD) or without ICD (PD-ON non-ICD), and OFF medication PD patients (PD-OFF). A neural network model of the Basal Ganglia (BG) that has the capacity to predict the dysfunction of both the dopaminergic (DA) and the serotonergic (5HT) neuromodulator systems was developed and used to facilitate the interpretation of experimental results. In the model, the BG action selection dynamics were mimicked using a utility function based decision making framework, with DA controlling reward prediction and 5HT controlling punishment and risk predictions. The striatal model included three pools of Medium Spiny Neurons (MSNs), with D1 receptor (R) alone, D2R alone and co-expressing D1R-D2R. Empirical studies showed that reward optimality was increased in PD-ON ICD patients while punishment optimality was increased in PD-OFF patients. Empirical studies also revealed that PD-ON ICD subjects had lower reaction times (RT) compared to that of the PD-ON non-ICD patients. Computational modeling suggested that PD-OFF patients have higher punishment sensitivity, while healthy controls showed comparatively higher risk sensitivity. A significant decrease in sensitivity to punishment and risk was crucial for explaining behavioral changes observed in PD-ON ICD patients. Our results highlight the power of computational modelling for identifying neuronal circuitry implicated in learning, and its impairment in PD. The results presented here not only show that computational modelling can be used as a valuable tool for understanding and interpreting clinical data, but they also show that computational modeling has the potential to become an invaluable tool to predict the onset of behavioral changes during disease progression.

## Introduction

Impulsivity is a multi-factorial problem that is assessed based on the capacity of an individual to accurately perform a goal directed action, and their ability to inhibit action impulses from interfering with the execution of the goal directed action [1–3]. It is also defined as the tendency to act prematurely, and has been linked to motor and cognitive disorders [4]. Some tests for impulsiveness include action selection paradigms such as Go / NoGo tasks, activities assessing response alternation due to delays, contingency degradation, or devaluation [4,5]. All of the above tests measure the subject's capacity to optimise the trade-off between speed and accuracy. Impulsive behaviors are exhibited in these tasks in the form of shorter reaction times, lesser behavioral inhibition over the non-optimal actions, less perseveration, and higher delay discounting [6–8]. Impulsivity is also the hallmark of several other psychiatric disorders such as attention deficit hyperactive disorder, aggression, substance abuse, and obsessive compulsive disorder [6].

### Impulsivity in Parkinson's disease

Parkinson's disease (PD) is characterised by the loss of dopaminergic (DA) neurons in substantia nigra pars compacta (SNc) [9,10]. The key motor symptoms that mark PD are tremor, rigidity, akinesia, and advanced cases may exhibit freezing of gait [11,12]. However non-motor symptoms such as cognitive dysfunction, behavioral and sleep disorders, dysautonomia, psychiatric disorders such as depression and anxiety, are also common in these patients [13]. A class of people suffers from an inability to resist an inappropriate hedonic drive, eventually resulting in performance of unfavorable actions with harmful consequences. This inability is termed as impulse control disorder (ICD), and is displayed in around 14% of ON medication PD (PD-ON) patients who are mostly treated with DA agonists [14]. ICDs include pathological gambling, compulsive shopping, binge eating, punding, overuse of dopaminergic medication, and over-engaging in meaningless hobby-like activities. The reduction of the medication can induce withdrawal symptoms, thus demanding an optimal therapy to ameliorate both the motor and the non-motor symptoms in PD [15].

### Neural substrates identified for impulsivity

Reported neural substrates of impulsivity include cortical structures such as the prefrontal cortex and orbito-frontal cortex, as well as subcortical structures such as the striatum, subthalamic nucleus (STN), globus pallidum externa and interna (GPe and GPi) of the basal ganglia (BG) [8,16]. In-vivo neurochemical analysis in rats performing a serial reaction time task revealed that dysfunction in neuromodulators such as DA and serotonin (5HT) in the fronto-striatal circuitry is associated with impulsivity [8]. Specifically receptors such as DA D2, and 5HT 1,2,6 are shown to significantly contribute to the impulse control disorder [14,17,18]. Computational modelling can be used for a better understanding of the contribution of the above mentioned structures and neurochemicals to impulsive decision making, as is described below.

### Computational modelling of neural substrates of impulsivity

Since PD-ON ICD is primarily linked to impairment in DA signalling and the BG function, several contemporary models of PD-ON ICD have focused on the role of the BG, often by using a reinforcement learning (RL) framework [19,20]. In this framework, learning is driven by rewards and punishments obtained as a result of executing actions [21,22]. The prediction error which is the difference between expected and received rewards is signalled by DA. There is evidence supporting that mesencephalic DA signalling codes for the temporal prediction error in reinforcement learning framework [23–25]. Such a prediction error facilitates the computation of some "goodness" measures such as the value function associated with an action. The value function refers to the expected sum of the future rewards obtained on executing actions. Functional imaging studies suggest that value is computed in striatum of the BG [26,27]. This computation is thought to be achieved by combining the reward prediction error information from the SNc to striatum along with the cortical state conveyed to the striatum by corticostriatal projections [20,26–29].

PD-ON ICD patients are reported to display exaggerated reward learning and attenuated punishment learning [20]. This is in contrast to the OFF- medication PD patients (PD-OFF) who are more sensitive to punishments than rewards [30]. Evidence suggest that phasic DA signals are necessary for reward punishment learning. While positive phasic DA signals are necessary for reward learning, negative phasic DA signals (and the duration of phasic dip) are needed for punishment learning [25]. The loss of dopaminergic neurons and decreased levels of DA [31] is known to amplify phasic dips of DA and hence promote punishment learning. On the contrary, medication increases the basal firing of the dopaminergic neurons and the availability of tonic DA, thereby promoting reward learning. The opponency between the direct and indirect pathways of the BG, mediated by the available DA for a particular subject type, is utilized by several models to explain the ICD behavior [20,32–34].

Some models account for these differences in reward / punishment learning between the PD-ON and PD-OFF patients by invoking differential learning rates for positive and negative feedback learning [20]. According to one model, ICD is an effect of automaticity of the stimulus-response relationship that becomes insensitive to the outcome; thus ICD is thought to be a form of habitual action [14]. Another model that belongs to the actor-critic family of BG models localizes the critic module (which evaluates the *rewards* associated with an action) to ventral striatum, and the actor module (which provides an executable plan for performing actions) to dorsal striatum. A dysfunction in the critic module has been proposed to explain the impaired stimulus-response relationship in PD-ON ICD cases [30]. Some other models use matching law to relate the probability of selecting a choice among two given alternatives to both the relative magnitudes and relative delays of the reinforcers associated with the alternatives [6]. The preference to choices increased with the magnitude of the associated reinforcer, but decreased with the delay associated with the reinforcer. Increased sensitivity to delays was predicted to increase impulsive behavior in that study [6].

### Our modeling approach

In the case of medication-induced impulsivity in PD patients, there are many experiments reporting a non-significant role of DA in medication-induced forms of impulsivity, for example, delay discounting task [35–38]. And some experiments suggest that an impaired balance between 5HT and DA is the root of impulsivity [39–42]. Additionally, there are several instances of experimental studies that relate central 5HT and functional polymorphisms of the 5HT transporter gene to impulsivity [7]. Thus the ætiology of ICD in PD should involve dysfunction in both 5HT and DA systems [7,8]. Therefore a modeling approach that is based solely on DA mediated dynamics in the BG [20] should ideally be expanded to include the 5HT system for better representation of the experimentally observed behavior. Most of the models reviewed above consider only DA dysfunction to explain impulsivity behavior. There is clearly a need for a model that unifies the contributions of other neuromodulators such as 5HT in addition to DA, in order to gain a comprehensive understanding of impulsivity.

In this study, we propose a unified computational network model of the BG that can mimic impulsivity disorder. The model is cast in the RL framework. It explicitly includes the anatomical modules such as the striatum, GPe, GPi and STN [43–45]. In addition to these anatomical components, the model also incorporates the roles of two key neuromodulators implicated in ICD–DA and 5HT. In line with classical RL-based models of the BG, the DA signal corresponds to reward prediction error in the present model. Invoking the natural relationship between impulsivity and risk-seeking [46,47], we borrow elements from a recent model [28] that links 5HT and risk-based decision making, and incorporate them in the proposed model.

The paper is outlined as follows: Section 2 deals with the materials and methods along with the model approach. Section 3 is concerned with the experimental and the modelling results, which are then discussed in Section 4.

## Materials and Methods

### Participants

This study was part of a larger project conducted at Ain Shams University Hospital, Cairo, Egypt. Seventy six participants were recruited for the project containing 160 trials of a probabilistic learning task. The subjects include (1) PD patients tested OFF medication (PD-OFF, n = 26, 6 females); (2) PD patients without ICD tested ON medication (PD-ON non-ICD, n = 14, 3 females); (3) PD patients with ICD tested ON medication (PD-ON ICD, n = 16, 2 females); and (4) healthy controls (n = 20, 3 females). The healthy control participants did not have any history of neurological or psychiatric disorders. The PD-OFF group was withdrawn from medications for a period of at least 18 hours. The majority of ON-medication patients were taking dopamine precursors (levodopa-containing medications) and D2 receptor agonists, specifically, Requip, Mirapex, Stalevo, Kepra, and C-Dopa. The mean disease duration was 8.35, 9.56, and 9.8 years for PD-ON non-ICD, PD-ON ICD, and PD-OFF patients, respectively. The OFF medicated PD patients had 9.8 years of mean disease duration. All participants gave written informed consent and the study was approved by the ethical board of Ain Shams University.

The Unified Parkinson’s Disease Rating Scale (UPDRS) was used to measure the severity of PD [48]. The UPDRS for all patients were measured ON medication. There was no significant difference among the patient groups in their UPDRS scores (F(2,63) = 0.5432, p = 0.5836) and their MMSE scores (F(2,63) = 0.5432, p = 0.5836). All participants were also tested for intact cognitive function and absence of dementia with the Mini-Mental Status Exam- MMSE [49]. Furthermore, there were no significant difference between the patient groups on the North American Adult Reading Test [50], the Beck Depression Inventory [51], and the forward and backward digit span tasks (p > 0.05 in each case using one-factor ANOVA analysis). The scores of all patient groups in Barratt impulsiveness scale were significantly different from each other (F(2,63) = 9.3264, p = 0.0003). A post hoc t- test with two tail analysis showed that ICD patients contributed mostly to the differences observed in the scores.

### Task

**Fig 1:**
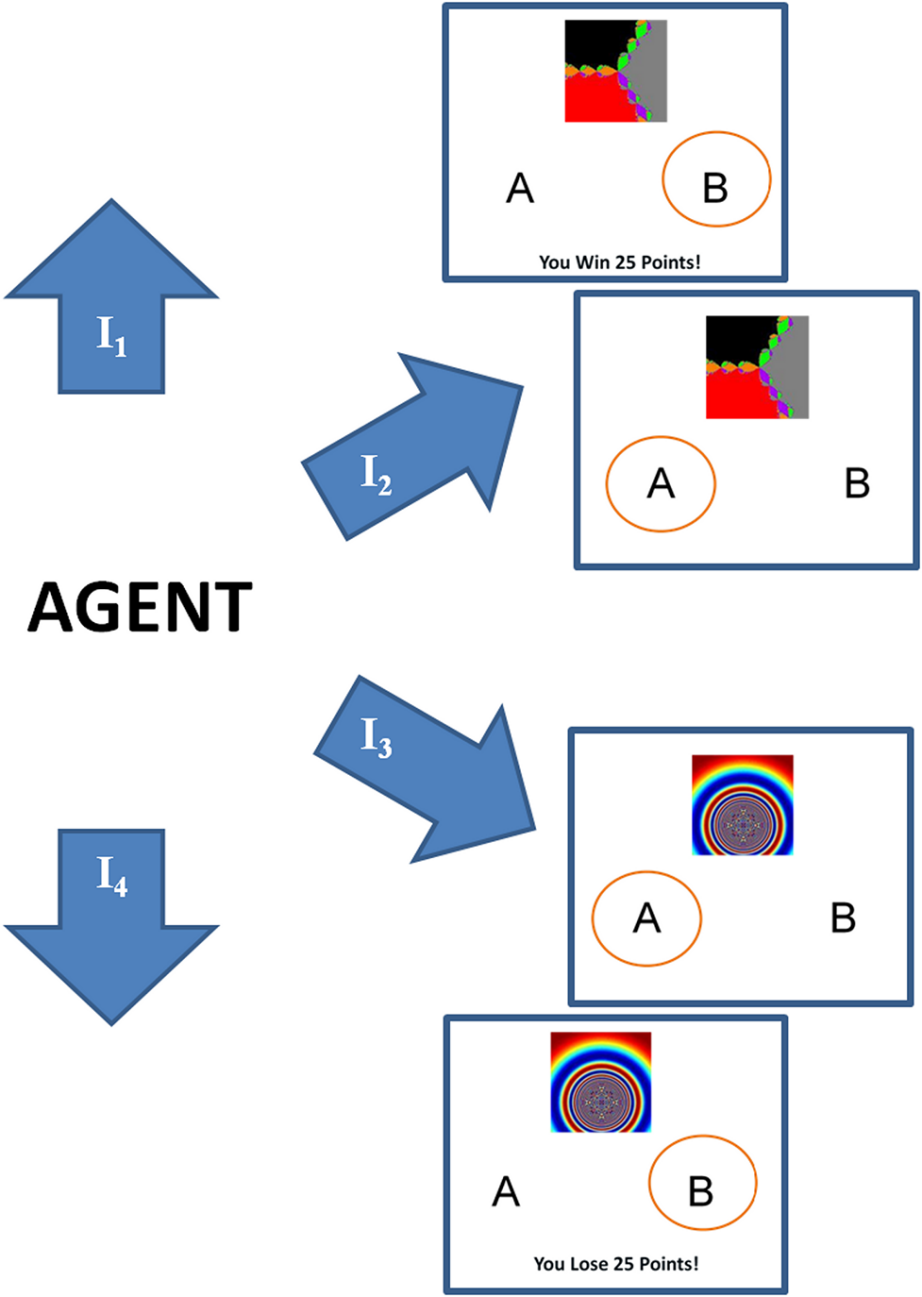
Experimental setup and a schematic of the task. The highlighted circles denote instances of the response selected for receiving an outcome. The images are represented by I1, I2, I3, I4 whose details are provided in Table 1. And the outcomes are presented to the subjects as "You Lose 25 Points", "You Win 25 Points", or none.

The experimental paradigm encompasses probabilistic reward and punishment learning. There were 160 trials wherein each trial, one of four different stimuli (I_1_, I_2_, I_3_, and I_4_) was presented in a pseudorandomized manner. The participants were asked to categorise them to response A or B. Two stimuli (I_1_ and I_2_) were used for testing the reward learning, and the other two stimuli (I_3_ and I_4_) were used for testing the punishment learning. An outcome follows every response, and an optimal response is the one maximising the observed outcome. In reward trials, an optimal response leads to +25 points 80% of the time and no reward for 20% of trials. In contrast, a non-optimal response resulted in +25 points only 20% of the time. In punishment trials, an optimal response resulted in no reward 80% of the time, and -25 points 20% of the time. Whereas a non-optimal response resulted in -25 points 80% of the time (Table 1, Fig 1). This experiment has been previously performed with PD patients and healthy control subjects as described in [30] but the present study extends the same experimental setup to analyse the subject's reaction times.

**Table 1:** Experimental setup and a schematic of the task.

Learning	Reward		Punishment	
**Image presented**	I_1_	I_2_	I_3_	I_4_
**Optimal type**	A	B	A	B
**Probability(points)**	0.8(+25)	0.8(+25)	0.8(0)	0.8(0)
**For optimal type**	0.2(0)	0.2(0)	0.2(-25)	0.2(-25)
**Non-optimal type**	B	A	B	A
**Probability(points)**	0.2(+25)	0.2(+25)	0.2(0)	0.2(0)
**For non-optimal type**	0.8(0)	0.8(0)	0.8(-25)	0.8(-25)

### Model framework

**Fig 2:**
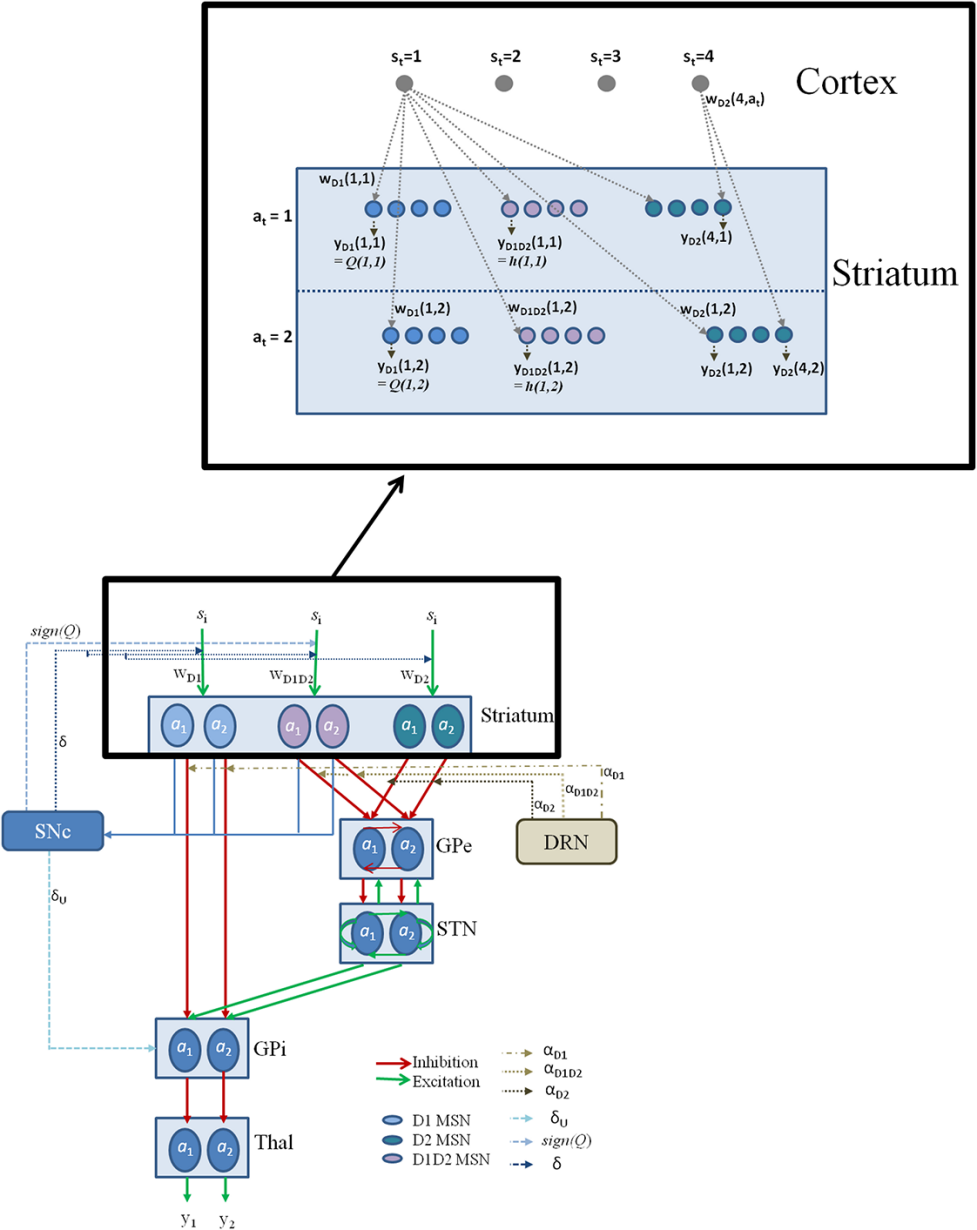
The complete network model of the BG used for the task setup. The BG model components shown are striatum, GPe, GPi, and STN along with SNc, DRN, and Thalamus. The schematic also denotes various DA and 5HT model correlates, as described in the Section: Model framework. The inset details the notations used in model section for representing cortico-striatal weights (*w*) and responses (*y*) of various kinds of MSNs (D1R expressing, D2R expressing, and D1R-D2R co-expressing) in the striatum, with a sample cortical state size of 4, and maximum number of action choices available for performing selection in every state as 2.

Our earlier modeling study [28] showed that the role of the BG in risk-based decision making can be efficiently modeled using utility-based learning, rather than just the value-based learning [19,20,52]. In utility-based learning, the utility of a state and an action pair is a combination of its value function and risk function. The state referred to here is the cortical state that forms the input of the BG, and the action refers to the behavioral response. The striatum of the BG receives input from a wider area of cortex including the pre-frontal cortex, orbito-frontal cortex, and sensory-motor cortices [8]. These nuclei also receive numerous 5HT and DA projections that are proposed to control the perception of value and variance / risk associated with the sampled rewards, respectively [28]. The striatal projections then project to the GPe, STN and GPi through the direct or indirect pathways; which together contribute to the action selection dynamics [43]. The framework used in this study is adapted from classical BG models as proposed in [53–56]. A detailed schematic representation of the current model is provided in Fig 2.

While the value function represents expected reward, risk function tracks reward and reward prediction error's variance over time [28,45,57,58]. Using a utility-based approach [28], that combines value and risk, it was possible to model experiments on reward-punishment learning [59], time scale of the reward prediction [60] and risk-based learning [61]. Moreover, the study also reconciles the multifarious roles of 5HT in the BG, as instantiated in these experiments [59–61], within a single framework. The seemingly unrelated roles in controlling behavioral inhibition, time scale of reward / punishment predictions that control sensitivity to delays in receiving outcomes, and risk learning were captured in our model of utility based decision making—where 5HT is modeled as a parameter affecting the risk prediction error [28]. Hence the current study models 5HT to control the risk function, and uses the classical representation of DA in controlling the reward prediction error. We borrow the above mentioned key ideas from Balasubramani et al. (2014) [28] and present here a detailed network model of the BG (Fig 2) to understand the behavioral data collected from PD patients and healthy controls.

The utility function proposed in (Balasubramani et al 2014) is given below:

$$U_{t}(s_{t},a_{t})=Q_{t}(s_{t},a_{t})-\alpha \;sign(Q_{t}(s_{t},a_{t}))\;\sqrt{h_{t}(s_{t},a_{t})}$$ (1)

where *U*, *Q*, and *h* are respectively the utility, action value and the risk functions associated with a state, 's' and action, 'a' at time, 't'. Risk sensitivity is controlled by the parameter α in the above Eq (1) and is proposed to represent the neuromodulator 5HT.

This lumped model has been extended to the BG network model with the value and the risk functions computed by the medium spiny neurons (MSNs) in the striatum [45]. Our earlier study proposed that striatal DA receptor (D1R) expressing MSNs code for value function, while the MSNs co-expressing both D1R and D2R (D1R-D2R) code the risk function. Whereas the D1R MSNs project via the direct pathway (DP) to GPi, the D2R and the D1R-D2R co-expressing MSNs project to the GPe in the indirect pathway (IP) [45] (Fig 2).

The outputs of the different kinds of MSNs—D1R expressing, D2R expressing and the D1R-D2R co- expressing neurons–are represented by variables *y*_D1_, *y*_D2_, and *y*_D1D2_, respectively in Eq (2). The subscript *t* denotes the time of response.

$$\begin{array}{l} y_{D1,t}(s_{t},a_{t})\;=w_{D1}(s_{t},a_{t})\;x(s_{t})\\ y_{D2,t}(s_{t},a_{t})\;=w_{D2}(s_{t},a_{t})\;x(s_{t})\\ y_{D1D2,t}(s_{t},a_{t})\;=w_{D1D2}(s_{t},a_{t})\;x(s_{t}) \end{array}$$ (2)

In the above equations, '*x*' is a logical variable modeled to be equal to 1 for the current state, *s*_t_, i.e., *x*(*s*_i_) = 1 *if s*_i_ *= s*_t_ (see Fig 2 inset). The Utility, *U*, is then obtained from the network model as described in the following Eq (3) [45].

$$U_{t}(s_{t},a_{t})=Q_{t}(s_{t},a_{t})-\alpha_{D1D2}\;sign(Q_{t}(s_{t},a_{t}))\;\sqrt{h_{t}(s_{t},a_{t})}$$ (3)

where

$$\begin{array}{l} Q_{t}(s_{t},a_{t})=y_{D1,}{}_{t}(s_{t},a_{t})\\ h_{t}(s_{t},a_{t})=y_{D1D2,t}(s_{t},a_{t}) \end{array}$$

Here in Eq (3), the risk sensitivity parameter is defined by *α*_D1D2_ which denotes the specific modulation of 5HT on the D1R-D2R co-expressing MSNs coding the risk function. The model DA parameter is used for the updating of cortico-striatal weights, and also controlling the switching at GPi [43]. Thereby the model postulates multiple forms of DA and 5HT signals, each of which has a differential action on D1R, D2R, and the D1R-D2R MSNs, as detailed later in this section. The bi-directional connectivity in the STN-GPe system that facilitates complex oscillations and "exploratory" behavior is also captured in this model [62]. We now present equations for the individual modules of the proposed network model of the BG. The reader refer our earlier studies for more details [43,45].

#### Model components: Striatum

The Striatum is proposed to have three types of MSNs, D1R expressing MSNs, D2R expressing MSNs, and D1R-D2R co-expressing MSNs, all of which have their gain functions (*λ*) as described below in Eq (4). The *c*_1,_ *c*_2,_ *c*_3_ are constants that vary with the receptor type. The value function (*Q*) requires a continuously increasing gain as a function of DA in the MSNs, which is shown to occur in the DA D1R containing MSNs. The risk function (*h*) [28,45,57,58] would simply require an increasing gain with increasing magnitude of DA, i.e. a 'U' shaped gain function which gives increased response with increasing *δ* ^*2*^. It is plausible that these risk-type of gain functions would then probably be exhibited by the neurons that co-express both the D1R-like gain function that increases as a function of DA, and D2R-like gain function that decreases as a function of DA [63–66], as identified in a recent experimental study [67]. The D2R MSN's gain function whose activity decreases as a function of DA makes them suitable for punishment computation, in opposition to that of the D1R MSNs responding positively to the reward prediction error (DA).

$$\begin{array}{l} \lambda_{D1}(\delta )=\frac{2c_{1}}{1+\exp(c_{2}(\delta +c_{3}))}-1\\ \lambda_{D2}(\delta )=\frac{2c_{1}}{1+\exp(c_{2}(\delta +c_{3}))}-1\\ \lambda_{h-D1}(\delta )=\frac{c_{1}}{1+\exp(c_{2}(\delta +c_{3}))}\\ \lambda_{h-D2}(\delta )=\frac{c_{1}}{1+\exp(c_{2}(\delta +c_{3}))}\\ \lambda_{D1D2}(\delta )=\lambda_{h-D1}(\delta )+\lambda_{h-D1}(\delta ) \end{array}$$ (4)

The weight update equations for a given (state, action) pair in the different kinds of MSNs are provided in Eq (5).

$$\begin{array}{l} \Delta w_{D1}(s_{t},a_{t})\;=\eta_{D1}\lambda_{D1}(\delta (t))\;x(s_{t})\\ \Delta w_{D2}(s_{t},a_{t})\;=\eta_{D2}\lambda_{D2}(\delta (t))\;x(s_{t})\\ \Delta w_{D1D2}(s_{t},a_{t})\;=\eta_{D1D2}\lambda_{D1D2}(\delta (t))\;x(s_{t}) \end{array}$$ (5)

The *δ*'s in the weight update equations are computed for the immediate reward condition as provided in Eq (6). It represents the DA form of activity that updates the cortico-striatal weights and is the classical temporal difference (TD) error [21,68].

$$\delta (t)=r-Q(s_{t},a_{t})$$ (6)

#### STN-GPe system

In the network model of the STN-GPe system, STN and GPe layers have equal number of neurons, with each neuron in STN uniquely connected bidirectionally to a neuron in GPe. Both STN and GPe layers are assumed to have weak lateral connections within the layer. The number of neurons in the STN (or GPe) (Fig 2) is taken to be equal to the number of possible actions for any given state, *n* [69,70]. The dynamics of the STN-GPe network is given below.

$$\begin{array}{l} \tau_{s}\frac{dx_{i}^{STN}}{dt}=-x_{i}^{STN}+\sum_{j=1}^{n}W_{ij}^{STN}y_{i}^{STN}-x_{i}^{GPe}\\ y_{i}^{STN}=\tanh(\lambda_{STN}x_{i}^{STN})\\ \tau_{g}\frac{dx_{i}^{GPe}}{dt}=-x_{i}^{GPe}+\sum_{j=1}^{n}W_{ij}^{GPe}x_{i}^{GPe}+y_{i}^{STN}-x_{i}^{IP} \end{array}$$ (7)

$x_{i}^{GPe}$ - internal state (same as the output) representation of *i*th neuron in GPe;

$x_{i}^{STN}$ - internal state representation of *i*th neuron in STN, with the output represented by $y_{i}^{STN}$;

*W* ^*GPe*^ - lateral connections within GPe, equated to a small negative number *ϵ*_g_ for both the self (*i* = *j*) and non-self (*i* ≠ *j*) connections for every GPe neuron *i*_._

*W* ^*STN*^ - lateral connections within STN, equated to a small positive number *ϵ*_s_ for all non-self (*i* ≠ *j*) lateral connections, while the weight of self-connection (*i* = *j*) is equal to 1+ *ϵ*_s_, for each STN neuron *i*.

Both STN and GPe are modeled to have complete internal connectivity with every neuron in a layer connected to every other neuron in that layer with the same connection strength. That common lateral connection strength is *ϵ*_s_ for STN, and *ϵ*_g_ for GPe. Likewise, STN and GPe neurons are connected in a one-to-one fashion–*i* ^th^ neuron in STN is connected to *i* ^th^ neuron in GPe and vice-versa. For all the simulations presented below, we set *ϵ*_g_ = -*ϵ*_s_; the learning rates 1 / *τ*_S_ = 0.1; 1 / *τ*_g_ = 0.033; and the slope *λ*_STN_ = 3; *ϵ*_s_ = 0.12.

#### The DP and IP projections to GPi

The outputs of D1R expressing MSNs, transmitted over the direct pathway are computed as:

$$x_{t}^{DP}=\alpha_{D1}\;\lambda_{D1}(\delta_{U}(t))\;y_{D1,t}(s_{t},a_{t})$$ (8)

The outputs of the D2R and D1R-D2R expressing MSNs, transmitted to GPe via the indirect pathway, are computed as,

$$\begin{array}{l} x_{t}^{IP}=\alpha_{D2}\;\lambda_{D2}(\delta_{U}(t))\;y_{D2,t}(s_{t},a_{t})\;+\\ \;\alpha_{D1D2}\;sign(y_{D1,t}(s_{t},a_{t}))\;\lambda_{D1D2}(\delta_{U}(t))\;\sqrt{y_{D1D2,t}(s_{t},a_{t})} \end{array}$$ (9)

Description for the parameters *α*_D1_, *α*_D2,_ *α*_D1D2_ in the Eqs (8 and 9): The neuromodulator 5HT's specificity in expression along with a particular type of MSN is not known [71–74]. In the present model, 5HT is thought to modulate the activity of all three kinds of MSNs (D1R expressing, D2R expressing and the D1R-D2R co-expressing). Hence the modeling correlates of 5HT are the parameters *α*_D1_ (Eq (8))_,_ *α*_D2,_ *α*_D1D2_ (Eq (9)) for modulating the output of the D1R, D2R and the D1R-D2R MSNs respectively, and they represent the tonic-5HT modulation exerted by dorsal raphe nucleus (DRN) [75–77]. The utility function described in Eq (3) involves specifically the 5HT parameter, *α*_D1D2_, to represent the selective modulation on 5HT on the risk-coding D1R-D2R MSNs; it does not involve the *α*_D2_ parameter which represents the effect of 5HT on D2R MSNs in the striatum.

The variables *y*_D1,t_, *y*_D2,t,_ *y*_D1D2,t_ as a function of state (*s*) and action (*a*) at time, *t*, are obtained from Eq (2).

Description for the parameters *λ*_D1_, *λ*_D2,_ *λ*_D1D2_ in the Eqs (8 and 9): The D2R and the D1R-D2R MSNs form part of the striatal matrisomes known to project to the IP, while the D1R MSNs project to the DP [69,71,72,78,79]. It should also be noted that *λ*s used as a gain factor in Eqs (8 and 9) have different parameters from *λ*s used in Eq (5). And the gain functions in Eq (8 and 9) are a function of the DA form [80] which represents the temporal difference in utility function, *δ*_U_ (Eq (10)). This is different from the DA form, *δ*, described in Eq (6).

$$\delta_{U}(t)=U_{t}(s_{t},a_{t})-U_{t-1}(s_{t},a_{t-1})$$ (10)

Another *correlate of DA* (Fig 2) affecting the model is the *sign*(*Q*) term in the Eqs (3 and 9), that is a form of value function, *Q* [22,24]. This term ensures the non-linear risk sensitivity observed in subjects based on the nature of the outcomes: risk aversive for rewards and risk-seeking for punishments [28,46]. The *utility difference form of DA* (Eq (10)) is proposed to be computed in SNc using the value inputs from D1R MSNs, and the risk inputs from the D1R-D2R MSNs, for a particular (state, action) pair. Hence, both the D1R and the D1R-D2R MSNs form a part of the striatal striosomes that contribute to the computation of DA error signal in SNc [69,71,72,78,79]. A summary of different mathematical forms of DA and 5HT used in the present model are listed in Table 2. Utility is thought to be computed in the SNc where the projections from D1R and D1R-D2R MSNs converge; D2 MSNs are not modeled to project to SNc [69,71,72,78,79] (Fig 2). Therefore the utility in Eq (3) is constructed as a summation of the value function computed by the D1R MSNs and the risk function computed by the D1R-D2R MSNs. But the action selection dynamics at GPi involve all the three types of MSNs (D1R, D2R and the D1R-D2R MSNs) through Eqs (8 and 9).

**Table 2:** Summary of different DA and 5HT model correlates.

**DA**	*δ*_[_ _21__,_ _68__]_	Eq (6)
	*δ*_U [_ _43__,_ _45__,_ _80__]_	Eq (10)
	*sign*(*Q*) _[_ _22__,_ _24__]_	Eqs (3 and 9)
**5HT**	*α*_D1_	Eq (8)
	*α*_D2_	Eq (9)
	*α*_D1D2_	Eq (9)

#### Action Selection at GPi

Action selection at GPi is implemented using the combination of the DP and IP contributions as follows:

$$x_{i}^{GP_{i}}=-x_{i}^{DP}+w_{i}^{STN-Gpi}\;y_{i}^{STN}$$ (11)

Since D1R is activated at increased dopamine levels, higher dopamine levels favour activating DP (constituted by the projections of D1R MSNs) over IP. This is consistent with the nature of switching facilitated by DA in the striatum [81–84]. The relative weightage of the STN projections to GPi is represented by *w* ^STN-GPi^, and is set to 1 for all the GPi neurons in the current study.

#### Action Selection at Thalamus

GPi neurons project to thalamus through inhibitory connections. Hence the thalamic afferents can be simply expressed as a modified form of Eq (11).

$$x_{i}^{Thalamus_{i}}=x_{i}^{DP}-w_{i}^{STN-Gpi}\;y_{i}^{STN}$$ (12)

These afferents in Eq (12) activate thalamic neurons as follows,

$$\frac{dy_{i}^{Thalamus}}{dt}=-y_{i}^{Thalamus}+x_{i}^{Thalamus}$$ (13)

where $y_{i}^{Thalamus}$ is the state of the *i* ^th^ thalamic neuron. Action selected is simply the '*i*' (*i =* 1,2,..,n) whose $y_{i}^{Thalamus}$ first crosses the threshold on integration. In the case of many actions crossing the threshold at the same time, the action with maximum $y_{i}^{Thalamus}$ at that time is selected. The reaction times (RT) associated with the trial is the number of iterations required for $y_{i}^{Thalamus}$ of the selected action to reach the threshold [85–87]. The threshold value used in the current simulation is 1.815.

#### Modeling Parkinson’s disease

The PD version of the proposed model has the following features (Eq (14)) for OFF and ON medication. PD pathology is associated with a huge loss in SNc dopaminergic neurons [88]. Since DA levels are lower in PD than in healthy controls, the *δ* (Eq (6)) is clamped to an upper bound (*δ*_Lim_), and this marks the PD-OFF case. In the PD-ON case, there is a higher level of tonic DA available due to medication. This is modeled by a simple addition of a fixed constant (*δ*_Med_ denoting the medication levels) to the clamped *δ* [89–93].

$$\begin{array}{l} \delta (t)=\left\{ \begin{array}{l} {}[a,b]\;for\;controls\\ {}[a,\delta_{Lim}]\;for\;PD-OFF\\ {}[a,\delta_{Lim}+\delta_{Med}]\;for\;PD-ON \end{array}\right.\\ Clamping\;the\;availability\;of\;DA\;(PD-OFF):\\ if\;\delta >\delta_{Lim};\;\delta =\delta_{Lim}\\ Increase\;in\;the\;availability\;of\;DA\;due\;to\;medication:\\ \delta :=\delta_{}\;+\delta_{Med} \end{array}$$ (14)

## Results

### Experimental results

Behavioural performance was assessed by analysing the optimality of participant responses and their reaction times. First, proportions of optimal responding to reward and punishment stimuli were calculated for each participant. A one-way ANOVA revealed significant group differences between optimizing rewards (F(3,72) = 12.12, p = 1.64X10^-6^) and punishments (F(3,72) = 3.76, p = 0.01) (Table 3). Post hoc analysis showed increased differences existing in the distributions of PD-OFF and PD-ON ICD patients responses (p = 2.23x10^-7^) for having optimality in reward learning (Stimuli I1 and I2) as the factor of analysis, and (p = 0.003) while having optimality in punishment learning (Stimuli I3 and I4) as the factor of analysis. That is, PD-ON ICD patients showed increased reward optimisation and decreased punishment optimisation relative to PD-OFF patients. The PD-ON non-ICD patients and healthy controls showed comparatively equal reward and punishment based optimality.

**Table 3:** One way Analysis of Variance (ANOVA) for outcome valences (a) reward (b) punishment, and (c) subject's reaction time, taken as the factor of analysis.

	*Source of Variation*	*SS*	*df*	*MS*	*F*	*P-value*	*F crit*
(a)	Between Groups	12771.04	3	4257.01	12.12	1.64 x10^-06^	2.73
	Within Groups	25286.69	72	351.20			
	Total	38057.73	75				
(b)	Between Groups	1796.26	3	598.75	3.76	0.01	2.73
	Within Groups	11450.28	72	159.03			
	Total	13246.55	75				
(c)	Between Groups	45939.84	3	15313.28	11.63	2.65x10^-06^	2.73
	Within Groups	94765.95	72	1316.19			
	Total	140705.8	75				

This is performed to understand the significance of categorising the subjects to various sub-types for different valences.

A similar analysis was conducted on reaction times, revealing overall significant group differences (F(3,72) = 11.63, p = 2.65X10^-6^), as shown in Table 3. The post hoc analysis showed this difference to be driven by the RT distributions of the PD-ON non-ICD, for having significantly larger RT distributions than the PD-OFF groups (p = 7.39x10^-6^), whilst PD-ON ICD group did not differ significantly from healthy controls.

### Modeling results

The network model described in the previous section is now applied to the experimental data. The reward of 25 points is simulated as *r* = +1, the punishment of -25 points as *r* = -1, and 0 points is simulated by *r* = 0. The four kinds of images (I_1_, I_2_, I_3_, I_4_) are simulated as states (*s*), and the two kinds of responses (choosing A or B) for a given image are simulated as actions (*a*) (Figs 1 and 2).

**Fig 3:**
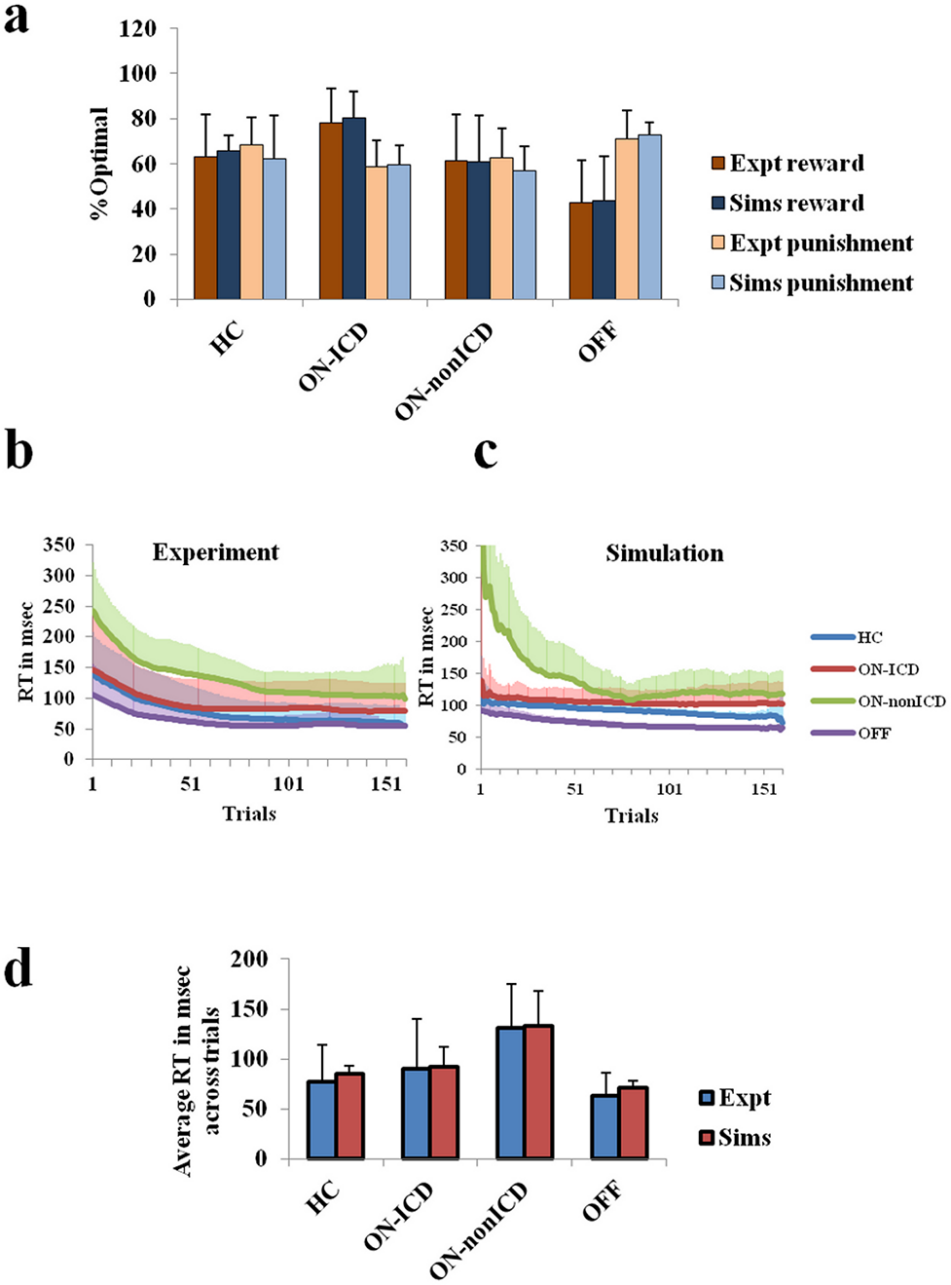
Analysing the action selection optimality and RT in the experiment and simulation for various subject categories. (a) The percentage optimality is depicted for various subject categories as obtained from the experimental data and the simulations (run for 100 instances). The reaction times (RT) in msec through trials are also shown for (b) the experimental data, and (c) for simulation. The average RTs in msec across the subject groups are provided for both experiment and simulation in Fig (d). The outliers are in prior removed with p = 0.05 on the iterative Grubbs test [133]. The similarity between the experiment and the simulation is analysed using a one way ANOVA, with reward valence, punishment valence, and RT as factors of analysis. They showed significant differences among the subject groups as seen in the experimental data, but no significant difference is observed between the simulation and the experiment. The subject categories healthy controls, PD-ON ICD, PD-ON non-ICD and PD-OFF are represented as HC, ON-ICD, ON-non-ICD, and OFF in the figures.

The experimental and the simulation results showing the selection optimality in the task-setup for different subject groups is shown in Fig 3A. Experimental reaction time analysis for every subject group is provided in the Fig 3B. The same is matched through our proposed model. The RT results from the simulation are shown in Fig 3C and 3D.

The modeling study suggests that optimising the parameters (Tables 4 and 5) related to DA- *δ* (viz. *δ*_Lim_ and *δ*_Med_ in Eq (14)), and 5HT–(*α*_D1_, *α*_D2_, *α*_D1D2_ in Eqs (3, 8 and 9)) are essential to model the ICD behavior in the PD patients. The following are the key modeling results:

1. An increased reward sensitivity in PD-ON, and increased punishment sensitivity in PD-OFF cases, are seen (Fig 3A)
2. Decreased reaction times are seen in ICD category of the PD-ON cases compared to that of the non-ICD PD-ON group (expt-Fig 3B, sims-Fig 3C, Fig 3D).
3. The model correlates of 5HT along with DA have to be optimized for improving the reward-punishment sensitivity in PD patients. The 5HT+DA model (*α*_D1D2_ > 0) captures the experiment profile better than just a DA model of the BG (*α*_D1_ = 1, *α*_D2_ = 1, *α*_D1D2_ = 0) (Table 5, S2 File, S3 File).
4. PD-ON ICD case required significantly reduced 5HT modulation of the striatal D2R (*α*_D2_) and the D1R-D2R (*α*_D1D2_) MSNs.
5. PD-ON non-ICD case is explained in our model by an increased 5HT modulation of D2R MSNs (*α*_D2_), and a decreased 5HT modulation of D1R-D2R MSNs (*α*_D1D2_).
6. A significant increase in the modulation of D2R MSNs (*α*_D2_) characterizes the PD-OFF case of the model. The above comparisons are made with respect to the healthy controls.

**Table 4:** Parametric values used for the Eqs (5,8 and 9), with *η*_D2_ = .1, *η*_D1D2_ = 0.1, and *η*_D1_ = .01.

Reference	Eq (5)				Eq (8)	Eq (9)		
	*λ*_*D1*_	*λ*_*D2*_	*λ*_*h-D1*_	*λ*_*h-D2*_	*λ*_*D1*_	*λ*_*D2*_	*λ*_*h-D1*_	*λ*_*h-D2*_
c_1_	1	1	.05	.05	1	1	.05	.05
c_2_	-50	50	-.01	.01	-50	50	-.01	.01
c_3_	0	-1	-.05	.05	0.01	0.01	-.05	.05

**Table 5:** The key parameters defining different subject categories.

	*α*_D1_	*α*_D2_	*α*_D1D2_	*δ*_lim_	*δ*_med_
Healthy controls	1	0.185	0.997	-	-
PD-OFF	1	0.991	0.033	0.001	-
PD-ON-ICD	1	0.046	0.001	0.001	0.06
PD-ON-non-ICD	1	0.916	0.160	0.001	0.06

#### Details of optimization

To investigate if the model can veritably predict differences in reaction time between the four different groups, given the selection accuracy alone, we performed the following tests:

Step 1: First, we identified parameter sets that are optimal for the cost function based on reward punishment action selection optimality only.
Step 2: We then selected solutions from Step 1 that can also explain the desired RT measures. The resulting parameter set is then taken as the optimal solution to the problem for a specific group.

The parameters for each experiment are initially selected using grid search and are eventually optimized using genetic algorithm (GA) [94] (Details of the GA option set are given in S1 File). The optimized parameter set for explaining the behavioral data in various subject groups is provided in Table 5. The procedure followed for optimizing the key parameters in the Table 5 using grid search are as follows:

1. The parameters *α*_D1_, *α*_D2_, and *α*_D1D2_ are optimized in the model of healthy controls.
2. For a model of PD-OFF, the parameters *α*_D1_, *α*_D2_, *α*_D1D2_, and *δ*_Lim_ are optimized to match the experimental results. Setting the parameter *δ*_Lim_ is a key addition to the PD-OFF model when compared to the healthy controls. This constraint reflects the deficit in DA availability in the model.
3. Then to explain action selection accuracy and reaction times of ICD in PD-ON medication case, *α*_D1_, *α*_D2_, *α*_D1D2_ and *δ*_Med_ are optimized. The *δ*_Lim_ value denoting DA deficit is kept the same as that obtained for the OFF medication case.
4. The non-ICD category of the PD-ON patients’ behavior is finally captured in the model by only optimising the parameters [*α*_D1_, *α*_D2_, *α*_D1D2_]. As mentioned above, *δ*_Lim_ is set to be the same in PD-ON (ICD and nonICD) and PD-OFF cases. Similarly, the medication level (*δ*_Med_) is maintained to be the same across the ICD and the non-ICD categories of the PD-ON patients. Hence the parameters differentiating the PD-ON ICD and the nonICD subjects are [*α*_D1_, *α*_D2_, *α*_D1D2_].

## Discussion

The aim of this study is to understand ICD in PD patients. Our experimental results suggest that the PD-ON ICD patients are more sensitive to rewards than to punishments. The PD-ON non-ICD patients had no significant difference between reward and punishment learning, similar to the healthy controls. The PD-OFF patients, on the contrary, showed significantly higher (Fig 3A) learning for punitive outcomes compared to rewarding outcomes. Within the PD-ON group, the ICD group showed shorter RTs than the non-ICD patients. The PD-OFF subjects were observed to have the shortest RT. Such trends in RT and reward-punishment based action selection accuracy have been reported previously in similar studies [20,30] on PD patients.

Application of the proposed network model to the experimental data suggests how impaired actions of DA and 5HT in the BG contribute to ICD behavior in the PD patients.

The proposed BG model uses utility function framework to model action selection and the associated reaction times [85,87,95–98]. This is an extended form of classical BG models as proposed in [53–56]. The oscillatory dynamics of the STN-GPe is modeled by using a simple Lienard oscillator model [43,62,99]. In the model, the BG system is thought to compute value and risk functions necessary for decision making [28,43]. Specifically, the DA-D1R containing MSNs compute the value function, whilst the co-expressing D1R-D2R containing MSNs compute risk function. Anatomical studies in primates reporting that D1R-D2R co-expressing MSNs form a significant proportion of the striatal MSNs [71,72,78,100]. The MSNs of the striatum project through the direct and the indirect pathways to the BG's output nuclei, GPi. The GPi then relays to the thalamus. Time taken for the activity of the winning thalamic neuron to reach a threshold corresponds to RT, while the index of winning thalamic neuron corresponds to the action selected.

The neuromodulators DA and 5HT affect BG dynamics in the model via different mechanisms as mentioned in Table 2. The variables that represent DA in the model are:

- the temporal difference error, *δ*, that updates the cortico-striatal weights [21,68],
- the temporal difference of utility [80], *δ*_U_, that aids the action selection at the GPi level [43], and
- the *sign*(value function) term controlling the response of D1R-D2R MSNs [22,24,45].

Likewise, 5HT differentially affects the D1R, D2R and D1R-D2R co-expressing MSNs, which is represented by the model parameters *α*_D1_, *α*_D2_, and *α*_D1D2_ respectively. Serotonin is proposed to control risk sensitivity in action selection performance of the BG [28]. Particularly, 5HT is shown to affect the D2R MSNs and co-expressing D1R-D2R MSNs (S2 File).

### Significance of the current study

Our previous study [28] has shown similarity between the effects of discount factor used to control *myopicity* of reward prediction [101] and the risk sensitivity factor (*α*) of Eq (1), in a delay discounting task. Some models relate impulsivity to discount factor, i.e., an increased discounting and myopicity in reward prediction is related to impulsive behavior [19,60,102]. We show that such effects can be captured in the proposed model by the risk sensitivity term (*α*_D1D2_) of the Eq (3) [28]. Furthermore, earlier models of ICD in PD only take DA deficiency in striatum into account [30], leaving behind other potential factors such as 5HT.

In some other models, reduced learning from the negative consequences in PD-ON ICD patients was modeled using an explicitly reduced learning rate parameter associated to negative prediction error [30]. But the proposed model naturally takes the nonlinearity in reward-punishment learning into consideration through the *sign*() term in risk function computation (Eq (3)). The nonlinearity mediated by *α*.*sign*() term towards rewards and punishments results in the PD-ON ICD case to learn more from rewarding outcomes, and the PD-OFF case to be more sensitive to punitive outcomes. The lower availability of DA leads to devaluation of the reward-associated choices more than that of the punishment in the PD-OFF case (Fig 3A) which favors punishment learning. Similarly in PD-ON cases, the punishment linked choices are overvalued to reduce the optimality in punishment learning.

Our model finds that the modulation of both DA and 5HT in the BG model is necessary to effectively explain the aspects of impulsive behaviour observed in our experiment. Please refer S2 File for computations showing the necessity of optimizing α_D1_, α_D2_, α_D1D2_ to explain the experimental data; and refer S3 File for computations showing that just DA related parameters cannot explain the experimental data. Using only the effect of D1R MSNs and D2R MSNs (α_D1_ = 1; α_D2_ = 1) without including the co-expressing D1R-D2R MSNs along with the 5HT effect (α_D1D2_ = 0), does not explain the experimental results (S2 File). This differentiates our model from those that invoke only the opponency between the DA mediated activity of D1R MSNs and D2R MSNs for explaining the PD-ON ICD behavior [20,32–34]. The main results from modeling of striatal MSNs are included in Table 6.

**Table 6:** Striatal MSNs and different sensitivities of decision making.

MSN	SENSITIVITY
D1R	Reward
D2R	Punishment
D1R-D2R	Risk

By investigating the functioning of neuromodulators DA and 5HT in this study, we find that there is a sub-optimal utility computation driven by the neuromodulators DA and 5HT in the PD patients. The clamping done to the availability of DA (Eq (14)) represents reduced DA availability or DA receptor density or dopaminergic projections to the BG in the PD-OFF case [103,104]. In the PD-ON case, an increased tonic level of DA is modeled by the addition of a medication constant (*δ*_Med_) [89–93]. Our model also predicts a lower availability of 5HT in the BG for both PD-OFF and PD-ON cases as previously reported by various experimental studies [9,105–107]. Specifically based on 5HT modulation in the model, a lowered sensitivity to the D2R MSNs and the D1R-D2R MSNs are observed in ICD. They exhibit a significantly reduced inhibition of actions along with risk-seeking behavior. Thus extremely low *α*_D2_ and *α*_D1D2_ efficiently differentiates ICD group among the PD-ON cases. The model also shows that the PD-OFF patients would have very high sensitivity to punishment (*α*_D2_) and increased behavioral inhibition, while the healthy controls have a higher sensitivity to risk (*α*_D1D2_).

Concisely, the model classifies the medication induced ICD in the PD patients to be possessing limited DA and altered 5HT modulations particularly on the D2R and D1R-D2R MSNs.

### Limitations of the study and future work

The co-expressing D1R-D2R MSNs are experimentally shown to significantly contribute to both the direct and the indirect pathways of the BG [71,72,108]. These two distinct pools of D1R-D2R MSNs—one following DP that controls exploitation, and the other following IP that controls exploration [43,44,62], might be used for modeling the non-linearity in risk sensitivity based on outcomes (i.e., risk aversion during gains and risk seeking during losses) [46]. The inherent opponency between the DP and IP [55,109] pathway would facilitate the projections of corresponding D1R-D2R MSNs for showing contrasting risk sensitive behavior. Each of the neuronal pools computing the risk function should then be weighed by appropriate sensitivity coefficients (representing neuromodulators DA and 5HT [28]) to capture the non-linear risk sensitive behavior [46] based on the valence of outcomes (Eq (3)). This is simplified in the present modeling study by considering the projections of D1R-D2R MSNs to IP alone, multiplied by a (*α sign(Q)*) term. Moreover, the increased magnitude of risk associated with an action is experimentally found to enhance exploration in the dynamics [110–112]. This is made possible in the model by routing the co-expressing D1R-D2R MSN activity to the IP, since in the present model it is IP that predominantly controls levels of exploration [43,44,62]. Moreover, there is evidence supporting the involvement of STN in controlling impulsivity [113], as their lesions are shown to decrease RT and increase premature responding behavior [95,114–116]. Also, the levels of synchronisation in STN-GPe contribute to the cognitive symptoms namely impulsivity [98,117], similar to its contribution to the motor symptoms in PD namely tremor, postural instability and gait disturbances [118–120]. In PD, markedly depleted levels of DA are associated with highly synchronised firing pattern and a slight increase in firing rates in STN [121,122]. These are the motivations behind specifically considering the projections of D1R-D2R MSNs to IP only. Expanding the framework to include the D1R-D2R MSNs projections to GPi (in the DP) would be incorporated in our future work. We would also involve the detailed neuronal modeling of STN-GPe system in our future work, to understand the possible role of oscillatory activity of STN in PD-related impulsivity [98,117].

Projections from GPe to GPi are found in the primates [56,123,124]. GPe projections to GPi are thought to be more focused, compared to the more diffuse projections of STN to GPi. These GPe-GPi connections bypass the GPe-STN-GPi connectivity—wherein the former are thought to perform a *focused suppression of GPi response to a particular action*, whereas the latter impose a *Global NoGo* influence [56,125]. Though the functional significance of these connections is not known, not accounting for this connectivity (STN-GPe-GPi) is a limitation of the modeling study. However, since we do not differentiate a global / local NoGo in our study, the proposed minimal model adapted from classical BG models [53–56] is demonstrated to capture the required experimental results at the neural network level.

STN also receives extensive norepinephrine (NE) afferents from locus ceruleus (LC) [125,126]. Furthermore since the dynamics of STN-GPe is strongly controlled by the neuromodulator NE [127,128], we would like to explore the possible role of NE in the BG dynamics. Particularly, NE is expected to control the lateral connection strengths in STN-GPe, and the gain of cortical input [110,129,130] to striatum and STN. The control of response inhibition through STN is thought to be established through NE activity in STN, and a dysfunction in such control could be related to ICD [131,132]. A detailed model of STN-GPe dynamics and the effect of NE, could help us better understand the role of the STN-GPe system in impulsivity and design better deep brain stimulation protocols to cure ICD [20].

Although DA, 5HT and NE along with the STN-GPe dynamics figure prominently in the experimental studies on impulsivity, computational models that closely resemble the neurobiological data supporting all those factors do not exist. Our model becomes the first of its kind to include the contributions of both DA and 5HT in the ICD pathology, and present a better "bench to bedside" proposal.

## Supporting Information

S1 File Optimization details. (PDF)

S2 File Analysing the effect of parameters (α_D1,_ α_D2,_ α_D1D2_) in various conditions (healthy controls, PD-ON ICD, PD-ON non-ICD, PD-OFF). (DOCX)

S3 File Sensitivity analysis of the parameters controlling the model DA parameter. (PDF)

S4 File Experimental Data set. (ZIP)
