# Supplementary material for: Identifying the Basal Ganglia Network Model Markers for Medication-Induced Impulsivity in Parkinson's Disease Patients
Source: PLoS One. 2015 Jun 4;10(6):e0127542. doi: 10.1371/journal.pone.0127542 (PMC4456385; doi:10.1371/journal.pone.0127542)
Supplement: S1 File — (PDF) [file pone.0127542.s001.pdf]

Supporting Information File S1

The Genetic Algorithm (Goldberg, 1989b) option set for optimization is given in the following table. Optimization toolbox 6.0, Matlab R2011a, The Mathworks Inc. is used.

Table S1: Option set for the GA tool

**Option**	**Value**
Population Size	20
Crossover fraction	0.8
Elite count	4
Generation time	1000
Function tolerance	$1\ x10^{-6}$
Cost function	$(\text{Expt measure - Sims measure})^2$
