# Supplementary material for: Identifying the Basal Ganglia Network Model Markers for Medication-Induced Impulsivity in Parkinson's Disease Patients
Source: PLoS One. 2015 Jun 4;10(6):e0127542. doi: 10.1371/journal.pone.0127542 (PMC4456385; doi:10.1371/journal.pone.0127542)
Supplement: S2 File — (DOCX) [file pone.0127542.s002.docx]

**Supporting Information File S2**

This material deals with the analysis of different subsets of the group containing α_D1,_ α_D2,_ α_D1D2_. Since the final decision only depends on the relative magnitudes of the three terms defined above in eqns. (8, 9), the α parameters are varied at the most two at a time. Thus the different cases that can be analysed from this material are summarized by the following table S2. Here, ‘*’ indicates that corresponding coefficient is varied, while ‘1’ indicates that it is fixed at 1.

**Table S2: Different case sets (α_D1,_ α_D2,_ α_D1D2_) for analysis**

|  | α_D1_ | α_D2_ | α_D1D2_ |
| --- | --- | --- | --- |
| Case 1 | * | 1 | 1 |
| Case 2 | 1 | * | 1 |
| Case 3 | 1 | 1 | * |
| Case 4 | * | * | 1 |
| Case 5 | 1 | * | * |
| Case 6 | * | * | * |

The results (in this supporting file for the Healthy controls, PD-ON ICD, PD-ON nonICD, PD-OFF conditions) depict the ability of each of the cases to explain the experiment reported in the manuscript.

To investigate if the model can predict the correct solutions for the reaction times of different subject types, given the selection accuracy alone, we performed the following steps.

*Step 1:* Firstly the parameters (multiple solutions) are drawn for the cost function only involving the reward punishment action selection optimality.

From the following graphs, we see that there could be multiple solutions to the cost function minimizing only the error that arose from the reward-punishment action selection optimality.

*Step 2:* We then check for the solutions that can explain the desired RT measures too. This is from the set of solutions from *step 1* that can best represent the reaction times (RT) of various subject types. The resulting parameter set is then taken as the optimal solution to the problem, for a given subject type.

**Step 1:**

**The following figures for healthy controls, PD-ON ICD, PD-ON non-ICD, PD-OFF analyse the error as a function of [*α*_D1_, *α*_D2_, *α*_D1D2_].**

*Representing normalised Error = ((expt-sims)/expt)* **^2^** *summated for the % mean reward [rew] and the % mean punishment [pun] optimality.*

*Error = ((expt_rew_-sims_rew_)/expt_rew_)* **^2^** *+ ((expt_pun_-sims_pun_)/expt_pun_)* **^2^**

**Table: The Expt values used for the analysis**

|  | Healthy controls | PD-ON ICD | PD-ON nonICD | PD-OFF |
| --- | --- | --- | --- | --- |
| rew | 63.25 | 78.28 | 61.16 | 43 |
| pun | 68.31 | 58.82 | 62.66 | 71.3 |

(Note that in Step 1, the cost function involves only the reward-punishment optimality).

**Analysis for the healthy controls**

**
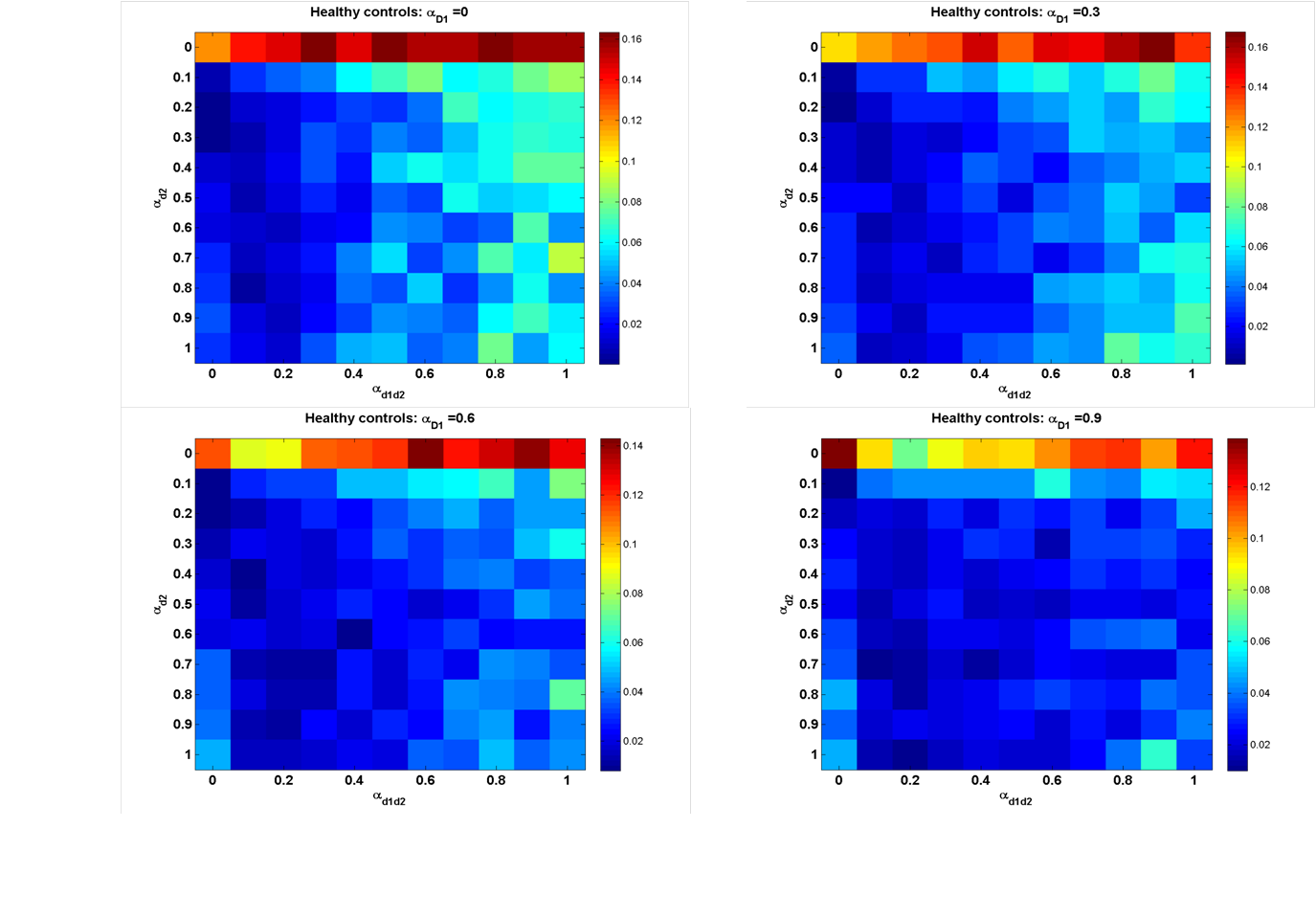
**

**Analysis for the PD-ON ICD**

**
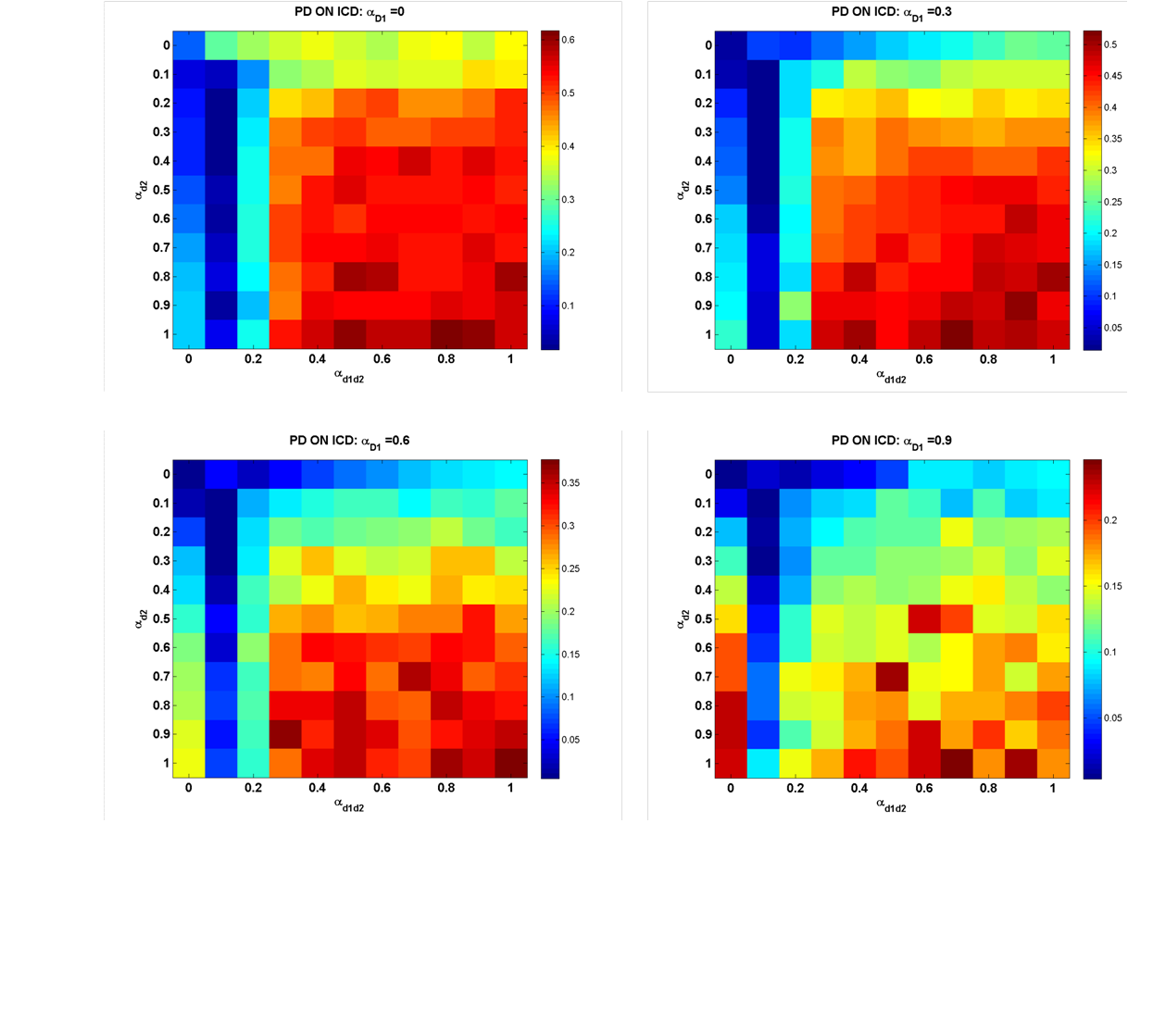
**

**Analysis for the PD-ON nonICD**

**
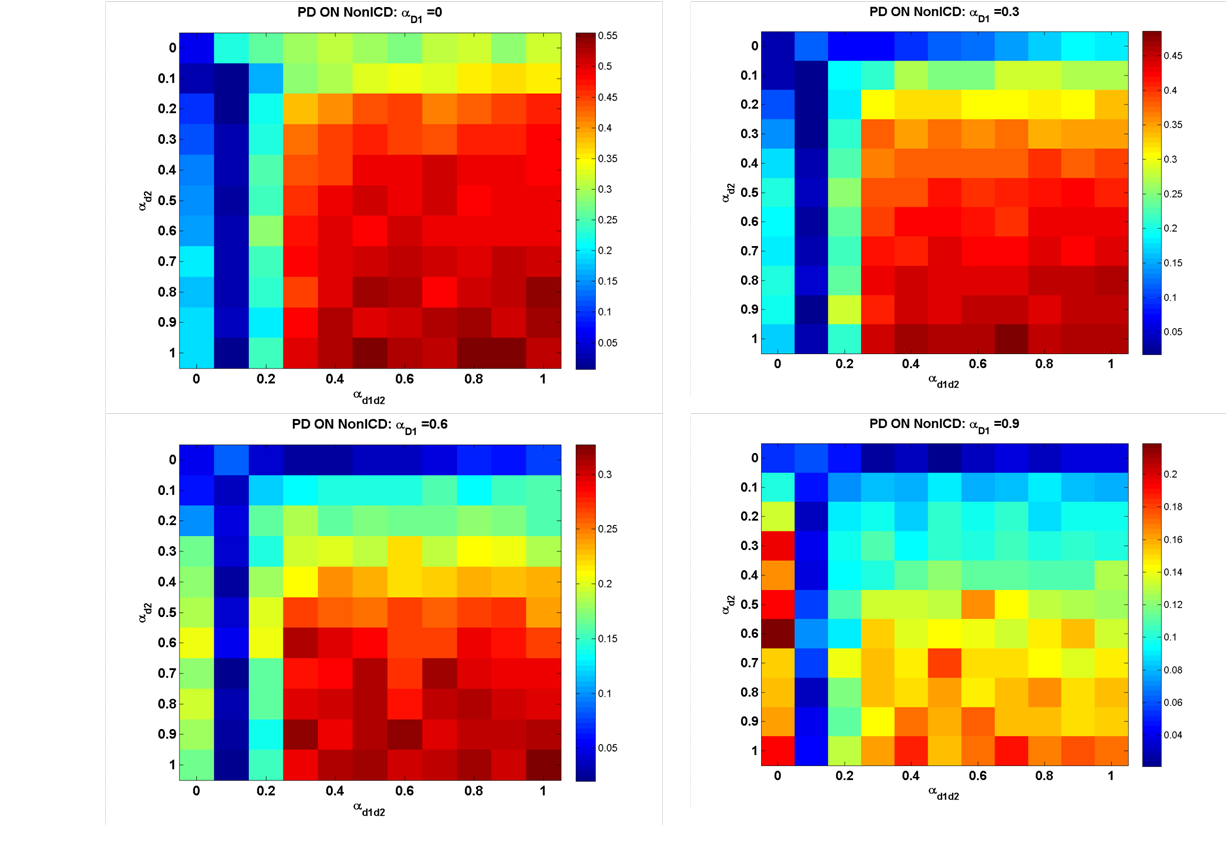
**

**Analysis for the PD-OFF**

**
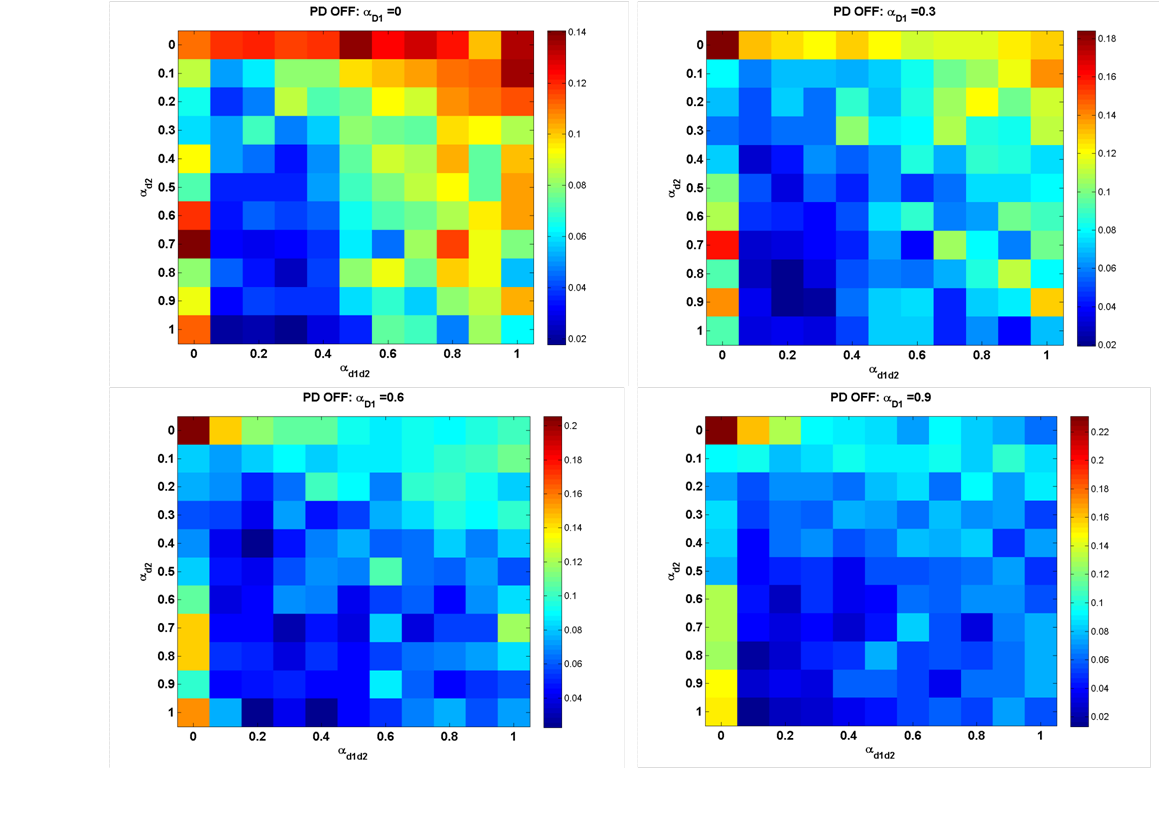
**

**Step 2:**

**The following figures for healthy controls, PD-ON ICD, PD-ON non-ICD, PD-OFF analyse the error as a function of [*α*_D1_, *α*_D2_, *α*_D1D2_].**

**Representing normalised Error = ((expt-sims)/expt)^2 summated for the % mean reward [rew] and the % mean punishment [pun] optimality, and also the mean reaction time [RT].**

**Error = ((expt_rew_-sims_rew_)/expt_rew_)^2 + ((expt_pun_-sims_pun_)/expt_pun_)^2 + ((expt_RT_-sims_RT_)/expt_RT_)^2**

**Table S3: The Expt values used for the analysis**

|  | Healthy controls | PD-ON ICD | PD-ON nonICD | PD-OFF |
| --- | --- | --- | --- | --- |
| RT | 76.78 | 90.19 | 131.11 | 62.81 |
| rew | 63.25 | 78.28 | 61.16 | 43 |
| pun | 68.31 | 58.82 | 62.66 | 71.3 |

**Analysis for the healthy controls**

**
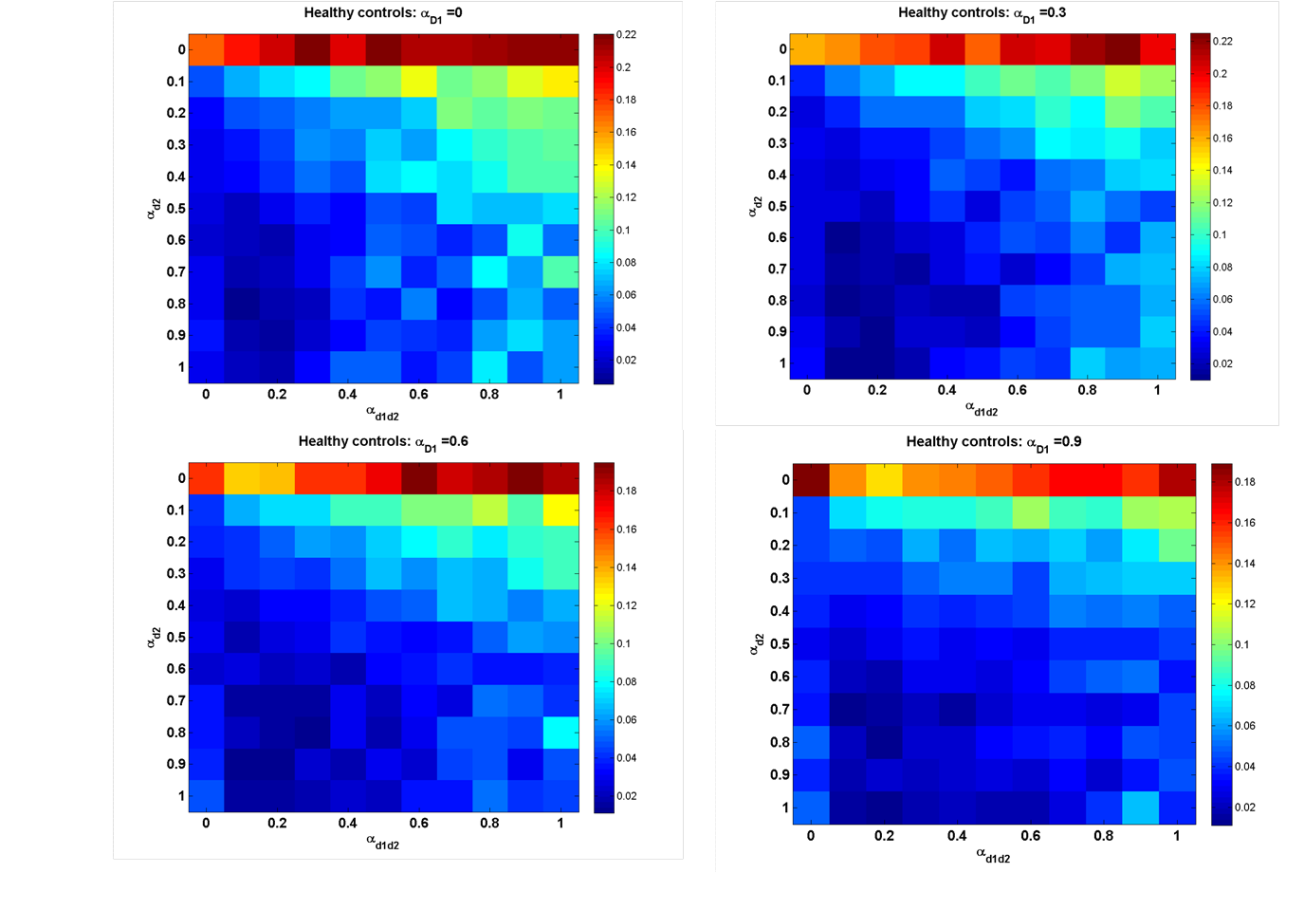
**

**Analysis for the PD-ON ICD**

**
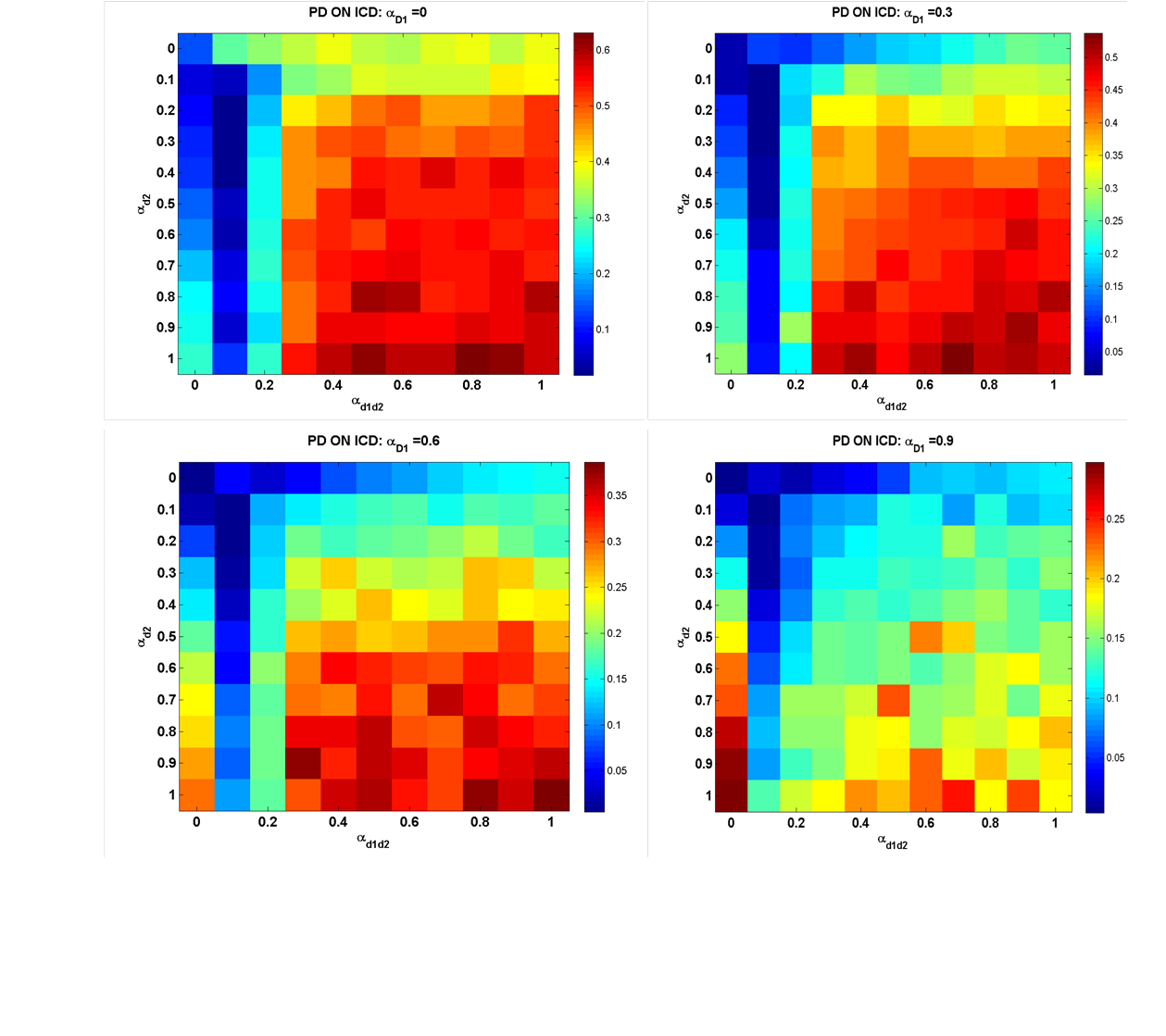
**

**Analysis for the PD-ON nonICD
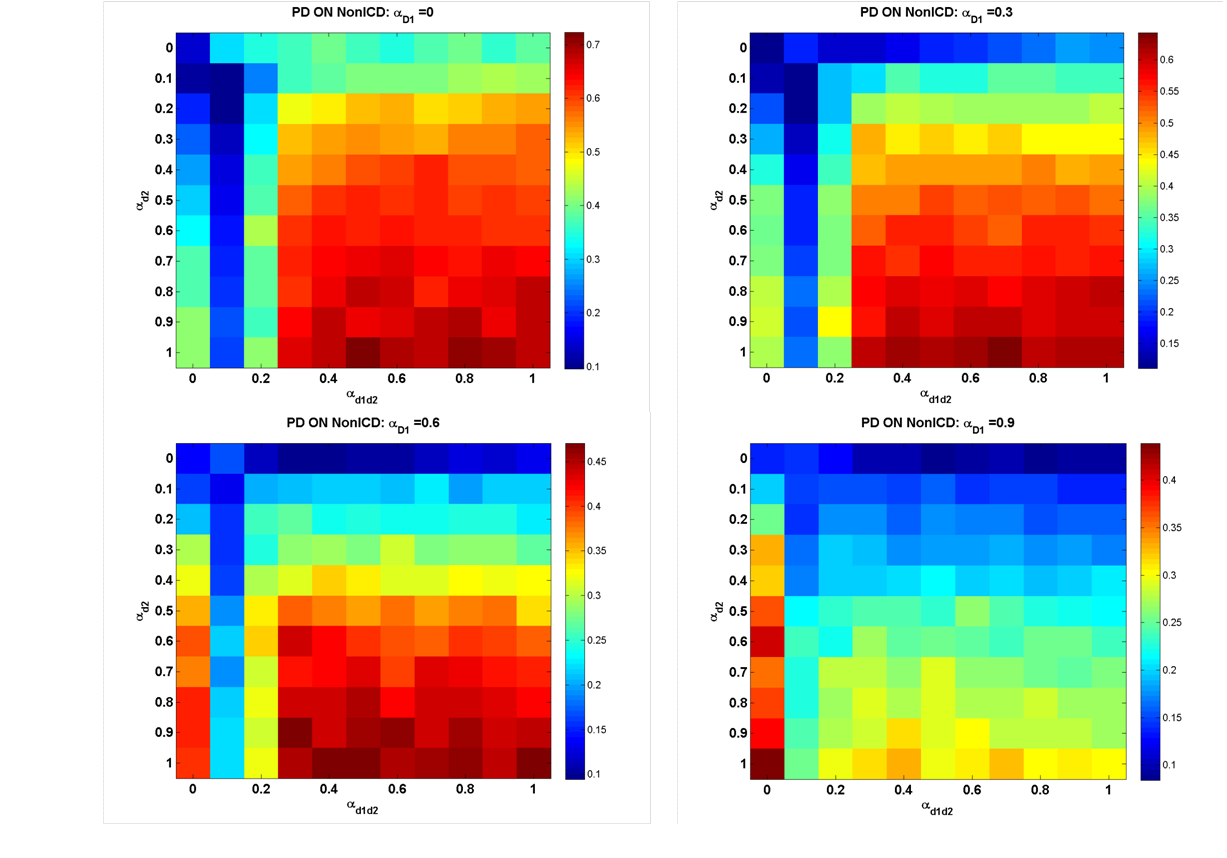
**

**Analysis for the PD-OFF**

**
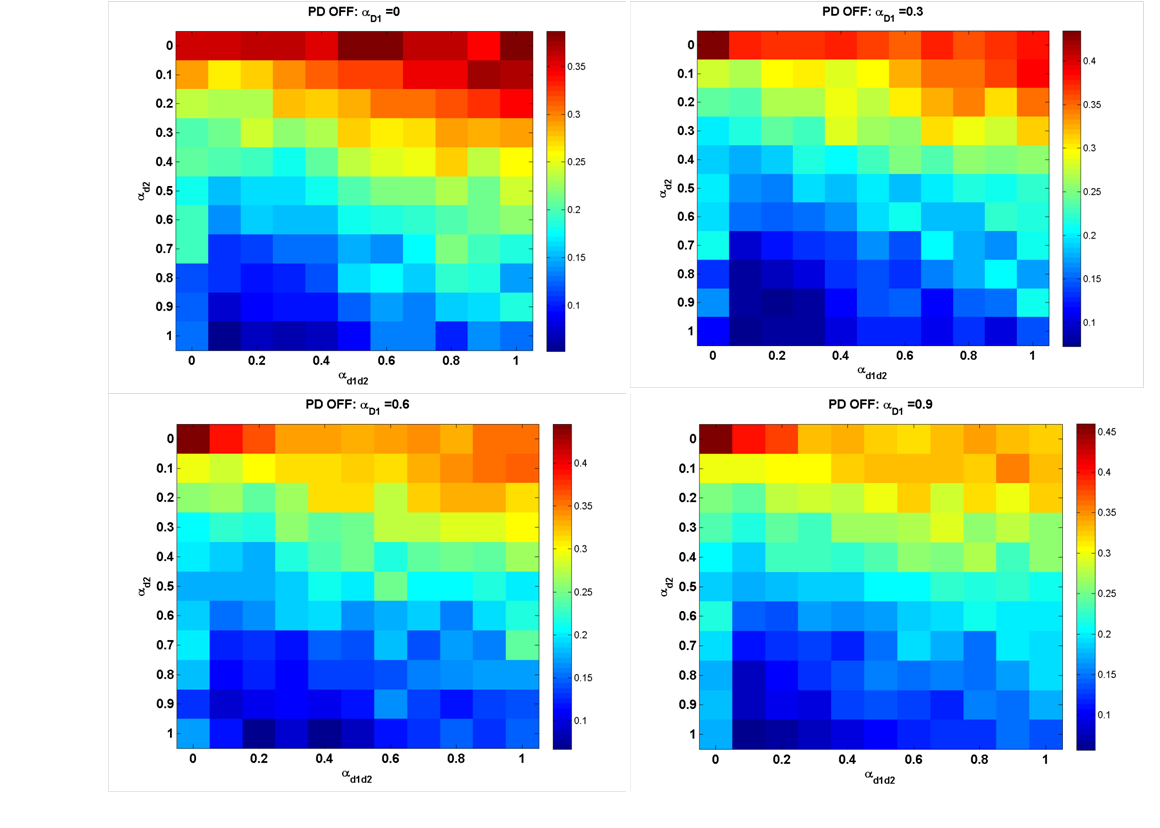
**

From the above figures, it can be noted that the derived solutions (with minimal error measures) after steps (1&2) are a subset of that obtained after step (1). Hence the 5HT model parameters (α_D1_ , α_D2_ , α_D1D2_) are not put there by design, and are contributing significantly for the RTs and action selection optimality. In fact they are able to narrow down and predict the solutions after step 1 itself.

The contributions of 5HT mediated D1R-D2R MSNs activity (α_D1D2_) was also found necessary. Refer high error measures for computations not considering activity from D1R-D2R MSNs (α_D1D2_ = 0 ), and considering only the activities from D1R and D2R MSNs (α_D1_ = 1; α_D2_ = 1) in our model. This is in contrast to the contemporary modelling literature possessing only the D1R and D2R MSNs.
