# Supplementary material for: Identifying the Basal Ganglia Network Model Markers for Medication-Induced Impulsivity in Parkinson's Disease Patients
Source: PLoS One. 2015 Jun 4;10(6):e0127542. doi: 10.1371/journal.pone.0127542 (PMC4456385; doi:10.1371/journal.pone.0127542)
Supplement: S3 File — (PDF) [file pone.0127542.s003.pdf]

Supporting Information File S3

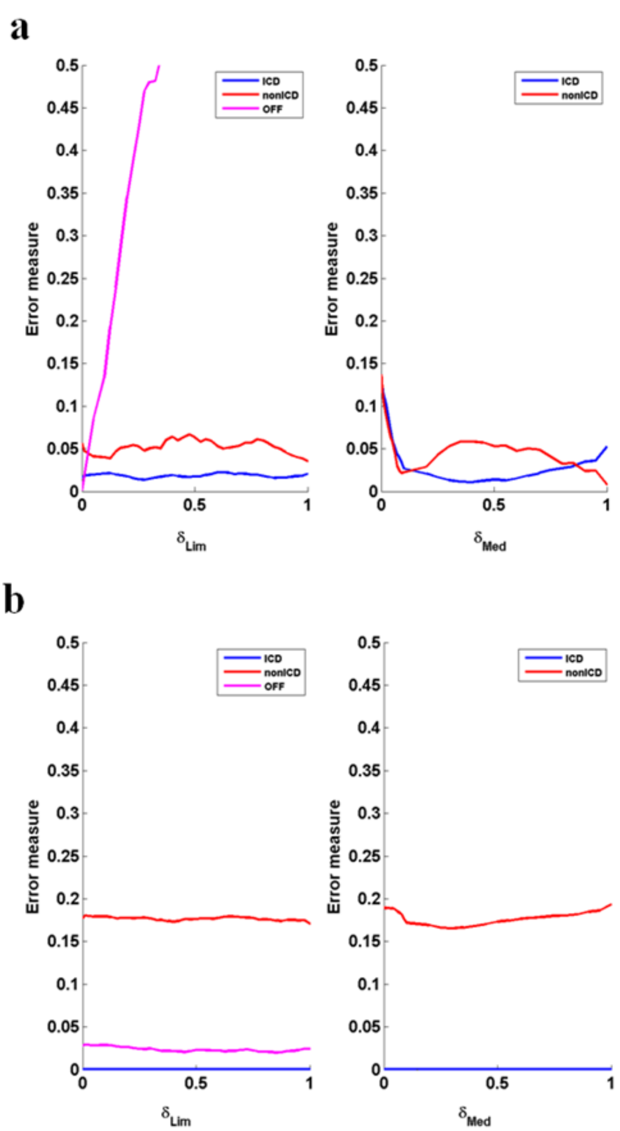

Figure S5: Sensitivity analysis of the parameters controlling DA (δ_{Lim}, δ_{Med}). The ranges adopted for the analysis are δ_{lim} = [0:0.1]; δ_{med} = [0:0.1], and they are normalised to be depicted in the same [0 1] x-axes scale limit; Each subplot in the above figure depicts the sensitivity of the parameter focussed in the 'title' by varying it over the mentioned range. The other three parameters are fixed to be at an operating point corresponding to the subject type (healthy controls, ICD, nonICD, OFF) as mentioned in the table 4b. The normalised error measure is calculated as the summation of ((expt-sims)/expt)^2 for measures: figure (a)- percentage reward optimality and percentage punishment optimality, and figure (b)- average reaction times in msec (RT), for a given subject-type. The results show the importance of modulating all the parameters (DA (δ_{Lim}, δ_{Med}) and that of 5HT) to match the accuracy and the reaction time of the model to the experimental results (see fig. 3 for the experimental results). Also refer Supplementary material B for more details.
