# Supplementary material for: Identifying the Basal Ganglia Network Model Markers for Medication-Induced Impulsivity in Parkinson's Disease Patients
Source: PLoS One. 2015 Jun 4;10(6):e0127542. doi: 10.1371/journal.pone.0127542 (PMC4456385; doi:10.1371/journal.pone.0127542)
Supplement: S4 File — (ZIP) [file pone.0127542.s004.zip › SMat/ICD1.doc]

```
QuartersCF4
Subject ID: PdmedBN3

Key: A/B is subj response, 0/1 is correct?, 0/1 is optimal?
1 Green_Pict,StimC,A,0,25   B 0  0    RT 650
2 CirclePict,StimA,A,25,0   A 1  1    RT 325
3 SticksPict,StimD,A,0,25   A 1  0    RT 231
4 Green_Pict,StimC,A,0,25   A 1  1    RT 152
5 SticksPict,StimD,A,0,25   A 1  0    RT 93
6 Worms_Pict,StimB,B,25,0   B 1  1    RT 178
7 Worms_Pict,StimB,B,25,0   A 0  0    RT 249
8 CirclePict,StimA,A,25,0   A 1  1    RT 375
9 Green_Pict,StimC,A,0,25   A 1  1    RT 480
10 Green_Pict,StimC,B,0,25   A 0  1    RT 194
11 Green_Pict,StimC,A,0,25   B 0  0    RT 173
12 SticksPict,StimD,B,0,25   A 0  0    RT 150
13 SticksPict,StimD,B,0,25   A 0  0    RT 125
14 Worms_Pict,StimB,A,25,0   B 0  1    RT 248
15 SticksPict,StimD,B,0,25   A 0  0    RT 96
16 CirclePict,StimA,A,25,0   B 0  0    RT 239
17 SticksPict,StimD,B,0,25   B 1  1    RT 169
18 Green_Pict,StimC,A,0,25   A 1  1    RT 186
19 CirclePict,StimA,B,25,0   A 0  1    RT 139
20 CirclePict,StimA,A,25,0   A 1  1    RT 213
21 SticksPict,StimD,B,0,25   A 0  0    RT 123
22 CirclePict,StimA,A,25,0   A 1  1    RT 457
23 SticksPict,StimD,B,0,25   B 1  1    RT 309
24 CirclePict,StimA,A,25,0   A 1  1    RT 86
25 Worms_Pict,StimB,A,25,0   B 0  1    RT 146
26 Green_Pict,StimC,A,0,25   A 1  1    RT 111
27 Worms_Pict,StimB,B,25,0   A 0  0    RT 223
28 CirclePict,StimA,B,25,0   A 0  1    RT 97
29 Worms_Pict,StimB,B,25,0   A 0  0    RT 139
30 CirclePict,StimA,A,25,0   A 1  1    RT 120
31 CirclePict,StimA,A,25,0   A 1  1    RT 152
32 Green_Pict,StimC,B,0,25   B 1  0    RT 162
33 Worms_Pict,StimB,B,25,0   B 1  1    RT 109
34 Green_Pict,StimC,A,0,25   A 1  1    RT 259
35 Worms_Pict,StimB,B,25,0   B 1  1    RT 100
36 Green_Pict,StimC,A,0,25   A 1  1    RT 419
37 SticksPict,StimD,B,0,25   B 1  1    RT 85
38 SticksPict,StimD,B,0,25   B 1  1    RT 60
39 Worms_Pict,StimB,B,25,0   B 1  1    RT 60
40 Worms_Pict,StimB,B,25,0   B 1  1    RT 241
41 Green_Pict,StimC,A,0,25   B 0  0    RT 170
42 CirclePict,StimA,A,25,0   A 1  1    RT 123
43 Green_Pict,StimC,A,0,25   A 1  1    RT 120
44 Worms_Pict,StimB,B,25,0   B 1  1    RT 85
45 Green_Pict,StimC,A,0,25   A 1  1    RT 63
46 CirclePict,StimA,A,25,0   A 1  1    RT 77
47 SticksPict,StimD,B,0,25   B 1  1    RT 111
48 CirclePict,StimA,A,25,0   A 1  1    RT 116
49 SticksPict,StimD,A,0,25   B 0  1    RT 94
50 Worms_Pict,StimB,B,25,0   B 1  1    RT 90
51 Worms_Pict,StimB,A,25,0   B 0  1    RT 126
52 SticksPict,StimD,B,0,25   B 1  1    RT 157
53 CirclePict,StimA,A,25,0   A 1  1    RT 111
54 CirclePict,StimA,B,25,0   A 0  1    RT 182
55 SticksPict,StimD,B,0,25   B 1  1    RT 129
56 CirclePict,StimA,A,25,0   A 1  1    RT 92
57 Green_Pict,StimC,A,0,25   A 1  1    RT 169
58 CirclePict,StimA,A,25,0   A 1  1    RT 69
59 Worms_Pict,StimB,B,25,0   B 1  1    RT 71
60 Green_Pict,StimC,A,0,25   A 1  1    RT 63
61 Worms_Pict,StimB,A,25,0   B 0  1    RT 69
62 Worms_Pict,StimB,B,25,0   B 1  1    RT 88
63 CirclePict,StimA,A,25,0   A 1  1    RT 66
64 SticksPict,StimD,B,0,25   A 0  0    RT 187
65 Worms_Pict,StimB,B,25,0   B 1  1    RT 190
66 Green_Pict,StimC,A,0,25   B 0  0    RT 335
67 SticksPict,StimD,A,0,25   B 0  1    RT 91
68 Green_Pict,StimC,A,0,25   A 1  1    RT 102
69 SticksPict,StimD,B,0,25   A 0  0    RT 85
70 SticksPict,StimD,B,0,25   A 0  0    RT 183
71 CirclePict,StimA,A,25,0   A 1  1    RT 63
72 Worms_Pict,StimB,B,25,0   B 1  1    RT 93
73 CirclePict,StimA,B,25,0   A 0  1    RT 65
74 Worms_Pict,StimB,B,25,0   B 1  1    RT 73
75 Green_Pict,StimC,B,0,25   A 0  1    RT 80
76 SticksPict,StimD,B,0,25   A 0  0    RT 136
77 Green_Pict,StimC,B,0,25   A 0  1    RT 163
78 Green_Pict,StimC,A,0,25   A 1  1    RT 134
79 SticksPict,StimD,B,0,25   A 0  0    RT 57
80 Worms_Pict,StimB,B,25,0   B 1  1    RT 155
81 CirclePict,StimA,A,25,0   A 1  1    RT 107
82 Worms_Pict,StimB,B,25,0   B 1  1    RT 72
83 Green_Pict,StimC,A,0,25   B 0  0    RT 210
84 CirclePict,StimA,B,25,0   A 0  1    RT 178
85 Green_Pict,StimC,A,0,25   B 0  0    RT 178
86 SticksPict,StimD,B,0,25   B 1  1    RT 345
87 SticksPict,StimD,B,0,25   B 1  1    RT 85
88 Worms_Pict,StimB,B,25,0   B 1  1    RT 80
89 CirclePict,StimA,A,25,0   A 1  1    RT 63
90 Worms_Pict,StimB,B,25,0   B 1  1    RT 174
91 SticksPict,StimD,A,0,25   B 0  1    RT 487
92 Green_Pict,StimC,B,0,25   B 1  0    RT 87
93 CirclePict,StimA,B,25,0   A 0  1    RT 99
94 CirclePict,StimA,A,25,0   A 1  1    RT 87
95 SticksPict,StimD,B,0,25   A 0  0    RT 61
96 SticksPict,StimD,B,0,25   B 1  1    RT 170
97 CirclePict,StimA,A,25,0   A 1  1    RT 70
98 Worms_Pict,StimB,B,25,0   B 1  1    RT 65
99 Worms_Pict,StimB,A,25,0   B 0  1    RT 86
100 Green_Pict,StimC,A,0,25   A 1  1    RT 279
101 Green_Pict,StimC,B,0,25   A 0  1    RT 144
102 Worms_Pict,StimB,B,25,0   B 1  1    RT 220
103 SticksPict,StimD,B,0,25   A 0  0    RT 126
104 SticksPict,StimD,A,0,25   A 1  0    RT 190
105 Green_Pict,StimC,A,0,25   A 1  1    RT 140
106 Green_Pict,StimC,A,0,25   A 1  1    RT 65
107 SticksPict,StimD,B,0,25   A 0  0    RT 141
108 Green_Pict,StimC,A,0,25   B 0  0    RT 365
109 Green_Pict,StimC,A,0,25   A 1  1    RT 363
110 Worms_Pict,StimB,B,25,0   B 1  1    RT 217
111 CirclePict,StimA,A,25,0   A 1  1    RT 68
112 Worms_Pict,StimB,B,25,0   B 1  1    RT 98
113 CirclePict,StimA,A,25,0   A 1  1    RT 147
114 SticksPict,StimD,B,0,25   A 0  0    RT 156
115 Worms_Pict,StimB,A,25,0   B 0  1    RT 154
116 SticksPict,StimD,B,0,25   A 0  0    RT 412
117 CirclePict,StimA,A,25,0   A 1  1    RT 478
118 Worms_Pict,StimB,B,25,0   B 1  1    RT 74
119 Green_Pict,StimC,A,0,25   A 1  1    RT 238
120 CirclePict,StimA,A,25,0   A 1  1    RT 81
121 CirclePict,StimA,A,25,0   A 1  1    RT 129
122 SticksPict,StimD,A,0,25   A 1  0    RT 227
123 SticksPict,StimD,B,0,25   A 0  0    RT 94
124 Worms_Pict,StimB,B,25,0   B 1  1    RT 149
125 Green_Pict,StimC,A,0,25   A 1  1    RT 319
126 Green_Pict,StimC,A,0,25   B 0  0    RT 242
127 Green_Pict,StimC,A,0,25   B 0  0    RT 353
128 CirclePict,StimA,A,25,0   A 1  1    RT 61
129 SticksPict,StimD,B,0,25   B 1  1    RT 320
130 Green_Pict,StimC,B,0,25   B 1  0    RT 154
131 CirclePict,StimA,A,25,0   A 1  1    RT 77
132 SticksPict,StimD,B,0,25   B 1  1    RT 89
133 Worms_Pict,StimB,B,25,0   B 1  1    RT 143
134 CirclePict,StimA,A,25,0   A 1  1    RT 69
135 CirclePict,StimA,B,25,0   A 0  1    RT 110
136 CirclePict,StimA,A,25,0   A 1  1    RT 76
137 SticksPict,StimD,A,0,25   B 0  1    RT 249
138 SticksPict,StimD,B,0,25   B 1  1    RT 165
139 CirclePict,StimA,A,25,0   A 1  1    RT 72
140 Worms_Pict,StimB,A,25,0   B 0  1    RT 87
141 Green_Pict,StimC,A,0,25   B 0  0    RT 485
142 SticksPict,StimD,B,0,25   A 0  0    RT 796
143 CirclePict,StimA,B,25,0   A 0  1    RT 245
144 Worms_Pict,StimB,B,25,0   B 1  1    RT 143
145 Worms_Pict,StimB,A,25,0   B 0  1    RT 82
146 CirclePict,StimA,A,25,0   A 1  1    RT 79
147 Worms_Pict,StimB,B,25,0   B 1  1    RT 155
148 Green_Pict,StimC,A,0,25   A 1  1    RT 472
149 SticksPict,StimD,B,0,25   A 0  0    RT 350
150 SticksPict,StimD,B,0,25   A 0  0    RT 180
151 Worms_Pict,StimB,B,25,0   B 1  1    RT 81
152 Green_Pict,StimC,A,0,25   B 0  0    RT 144
153 Green_Pict,StimC,B,0,25   B 1  0    RT 169
154 Worms_Pict,StimB,B,25,0   B 1  1    RT 72
155 SticksPict,StimD,B,0,25   B 1  1    RT 611
156 Worms_Pict,StimB,B,25,0   B 1  1    RT 85
157 Green_Pict,StimC,A,0,25   A 1  1    RT 334
158 CirclePict,StimA,A,25,0   A 1  1    RT 72
159 Worms_Pict,StimB,B,25,0   B 1  1    RT 86
160 Green_Pict,StimC,A,0,25   A 1  1    RT 155


Total AB Correct = 60 (75% correct)
Total CD Correct = 43 (53.75% correct)
Total AB optimal = 76 (95% optimal)
Total CD optimal = 43 (53.75% optimal)
Total points = 1075
```
