# Supplementary material for: Identifying the Basal Ganglia Network Model Markers for Medication-Induced Impulsivity in Parkinson's Disease Patients
Source: PLoS One. 2015 Jun 4;10(6):e0127542. doi: 10.1371/journal.pone.0127542 (PMC4456385; doi:10.1371/journal.pone.0127542)
Supplement: S4 File — (ZIP) [file pone.0127542.s004.zip › SMat/ICD2.doc]

```
QuartersCF4
Subject ID: PdmedBN7
 Stimuli:
A = CirclePict B= Worms_Pict
C = Green_Pict C= SticksPict
Stim A is class A 80% of trials; Stim B is class A 20% of trials
    win = 25 points, lose is 0
Stim C is class A 80% of trials; Stim D is class A 20% of trials
    win = 0, lose = 25 points


Key: A/B is subj response, 0/1 is correct?, 0/1 is optimal?
1 SticksPict,StimD,B,0,25   A 0  0    RT 889
2 Green_Pict,StimC,A,0,25   B 0  0    RT 234
3 Green_Pict,StimC,A,0,25   B 0  0    RT 221
4 CirclePict,StimA,A,25,0   A 1  1    RT 193
5 Worms_Pict,StimB,B,25,0   B 1  1    RT 432
6 Worms_Pict,StimB,B,25,0   A 0  0    RT 132
7 CirclePict,StimA,A,25,0   B 0  0    RT 129
8 CirclePict,StimA,A,25,0   B 0  0    RT 132
9 CirclePict,StimA,B,25,0   B 1  0    RT 111
10 SticksPict,StimD,A,0,25   B 0  1    RT 215
11 CirclePict,StimA,A,25,0   A 1  1    RT 91
12 SticksPict,StimD,B,0,25   A 0  0    RT 120
13 Worms_Pict,StimB,A,25,0   B 0  1    RT 79
14 SticksPict,StimD,B,0,25   A 0  0    RT 145
15 Worms_Pict,StimB,B,25,0   B 1  1    RT 127
16 Worms_Pict,StimB,B,25,0   A 0  0    RT 108
17 Green_Pict,StimC,A,0,25   B 0  0    RT 442
18 Worms_Pict,StimB,B,25,0   B 1  1    RT 590
19 SticksPict,StimD,B,0,25   B 1  1    RT 593
20 CirclePict,StimA,A,25,0   A 1  1    RT 69
21 CirclePict,StimA,A,25,0   A 1  1    RT 266
22 SticksPict,StimD,A,0,25   A 1  0    RT 143
23 Green_Pict,StimC,A,0,25   B 0  0    RT 601
24 Green_Pict,StimC,A,0,25   A 1  1    RT 114
25 Worms_Pict,StimB,B,25,0   B 1  1    RT 88
26 SticksPict,StimD,B,0,25   A 0  0    RT 102
27 Worms_Pict,StimB,B,25,0   B 1  1    RT 71
28 CirclePict,StimA,B,25,0   A 0  1    RT 163
29 CirclePict,StimA,A,25,0   A 1  1    RT 66
30 Green_Pict,StimC,B,0,25   B 1  0    RT 407
31 Worms_Pict,StimB,B,25,0   B 1  1    RT 87
32 SticksPict,StimD,B,0,25   A 0  0    RT 238
33 SticksPict,StimD,B,0,25   B 1  1    RT 43
34 Green_Pict,StimC,B,0,25   B 1  0    RT 121
35 Green_Pict,StimC,A,0,25   B 0  0    RT 65
36 Worms_Pict,StimB,A,25,0   B 0  1    RT 158
37 SticksPict,StimD,B,0,25   B 1  1    RT 185
38 Green_Pict,StimC,A,0,25   B 0  0    RT 97
39 CirclePict,StimA,A,25,0   A 1  1    RT 52
40 Green_Pict,StimC,A,0,25   B 0  0    RT 89
41 CirclePict,StimA,A,25,0   A 1  1    RT 36
42 Green_Pict,StimC,A,0,25   A 1  1    RT 84
43 CirclePict,StimA,B,25,0   A 0  1    RT 34
44 SticksPict,StimD,B,0,25   A 0  0    RT 63
45 Worms_Pict,StimB,B,25,0   B 1  1    RT 44
46 Worms_Pict,StimB,B,25,0   B 1  1    RT 45
47 CirclePict,StimA,A,25,0   A 1  1    RT 32
48 SticksPict,StimD,A,0,25   B 0  1    RT 43
49 Green_Pict,StimC,A,0,25   A 1  1    RT 105
50 CirclePict,StimA,B,25,0   A 0  1    RT 37
51 Green_Pict,StimC,A,0,25   B 0  0    RT 119
52 Worms_Pict,StimB,B,25,0   B 1  1    RT 65
53 CirclePict,StimA,A,25,0   A 1  1    RT 36
54 SticksPict,StimD,B,0,25   B 1  1    RT 141
55 Green_Pict,StimC,A,0,25   A 1  1    RT 95
56 Worms_Pict,StimB,A,25,0   B 0  1    RT 42
57 CirclePict,StimA,A,25,0   A 1  1    RT 31
58 SticksPict,StimD,B,0,25   A 0  0    RT 82
59 SticksPict,StimD,B,0,25   B 1  1    RT 45
60 CirclePict,StimA,A,25,0   A 1  1    RT 38
61 CirclePict,StimA,A,25,0   A 1  1    RT 32
62 Green_Pict,StimC,B,0,25   B 1  0    RT 393
63 SticksPict,StimD,B,0,25   A 0  0    RT 149
64 Worms_Pict,StimB,B,25,0   B 1  1    RT 46
65 Green_Pict,StimC,A,0,25   B 0  0    RT 44
66 SticksPict,StimD,B,0,25   B 1  1    RT 79
67 SticksPict,StimD,A,0,25   B 0  1    RT 41
68 Green_Pict,StimC,B,0,25   A 0  1    RT 86
69 Green_Pict,StimC,A,0,25   B 0  0    RT 50
70 Worms_Pict,StimB,B,25,0   B 1  1    RT 52
71 Worms_Pict,StimB,B,25,0   B 1  1    RT 33
72 SticksPict,StimD,B,0,25   A 0  0    RT 166
73 Green_Pict,StimC,A,0,25   B 0  0    RT 79
74 Worms_Pict,StimB,B,25,0   B 1  1    RT 39
75 SticksPict,StimD,B,0,25   A 0  0    RT 504
76 Worms_Pict,StimB,A,25,0   B 0  1    RT 37
77 CirclePict,StimA,A,25,0   A 1  1    RT 28
78 Worms_Pict,StimB,B,25,0   B 1  1    RT 32
79 CirclePict,StimA,A,25,0   A 1  1    RT 42
80 Green_Pict,StimC,A,0,25   A 1  1    RT 86
81 Worms_Pict,StimB,B,25,0   B 1  1    RT 41
82 Worms_Pict,StimB,B,25,0   B 1  1    RT 51
83 Green_Pict,StimC,A,0,25   A 1  1    RT 72
84 SticksPict,StimD,B,0,25   B 1  1    RT 77
85 Worms_Pict,StimB,B,25,0   B 1  1    RT 38
86 CirclePict,StimA,A,25,0   A 1  1    RT 42
87 SticksPict,StimD,B,0,25   B 1  1    RT 58
88 Worms_Pict,StimB,B,25,0   B 1  1    RT 34
89 SticksPict,StimD,B,0,25   A 0  0    RT 236
90 SticksPict,StimD,B,0,25   B 1  1    RT 64
91 SticksPict,StimD,A,0,25   B 0  1    RT 55
92 Green_Pict,StimC,A,0,25   B 0  0    RT 81
93 Green_Pict,StimC,A,0,25   B 0  0    RT 144
94 CirclePict,StimA,A,25,0   A 1  1    RT 30
95 Green_Pict,StimC,A,0,25   A 1  1    RT 77
96 Worms_Pict,StimB,B,25,0   B 1  1    RT 39
97 CirclePict,StimA,A,25,0   A 1  1    RT 26
98 Worms_Pict,StimB,B,25,0   B 1  1    RT 48
99 Green_Pict,StimC,A,0,25   A 1  1    RT 46
100 Worms_Pict,StimB,A,25,0   B 0  1    RT 55
101 Green_Pict,StimC,A,0,25   A 1  1    RT 92
102 CirclePict,StimA,A,25,0   A 1  1    RT 35
103 SticksPict,StimD,B,0,25   B 1  1    RT 37
104 Worms_Pict,StimB,A,25,0   B 0  1    RT 35
105 Green_Pict,StimC,A,0,25   A 1  1    RT 125
106 CirclePict,StimA,A,25,0   A 1  1    RT 31
107 CirclePict,StimA,A,25,0   A 1  1    RT 43
108 Green_Pict,StimC,B,0,25   A 0  1    RT 130
109 SticksPict,StimD,A,0,25   B 0  1    RT 149
110 CirclePict,StimA,B,25,0   A 0  1    RT 32
111 SticksPict,StimD,B,0,25   A 0  0    RT 86
112 Green_Pict,StimC,B,0,25   A 0  1    RT 178
113 Worms_Pict,StimB,B,25,0   B 1  1    RT 38
114 SticksPict,StimD,B,0,25   B 1  1    RT 44
115 CirclePict,StimA,A,25,0   A 1  1    RT 33
116 SticksPict,StimD,B,0,25   A 0  0    RT 78
117 CirclePict,StimA,B,25,0   A 0  1    RT 32
118 Worms_Pict,StimB,B,25,0   B 1  1    RT 36
119 CirclePict,StimA,A,25,0   A 1  1    RT 93
120 Green_Pict,StimC,A,0,25   B 0  0    RT 689
121 SticksPict,StimD,B,0,25   B 1  1    RT 522
122 Worms_Pict,StimB,B,25,0   B 1  1    RT 92
123 SticksPict,StimD,B,0,25   B 1  1    RT 212
124 CirclePict,StimA,A,25,0   A 1  1    RT 56
125 Worms_Pict,StimB,B,25,0   B 1  1    RT 48
126 CirclePict,StimA,A,25,0   A 1  1    RT 48
127 Worms_Pict,StimB,B,25,0   B 1  1    RT 49
128 Worms_Pict,StimB,B,25,0   B 1  1    RT 69
129 Green_Pict,StimC,A,0,25   A 1  1    RT 675
130 Worms_Pict,StimB,A,25,0   B 0  1    RT 56
131 Green_Pict,StimC,B,0,25   B 1  0    RT 78
132 SticksPict,StimD,A,0,25   A 1  0    RT 76
133 CirclePict,StimA,A,25,0   A 1  1    RT 50
134 CirclePict,StimA,B,25,0   A 0  1    RT 47
135 SticksPict,StimD,B,0,25   A 0  0    RT 148
136 CirclePict,StimA,A,25,0   A 1  1    RT 133
137 Worms_Pict,StimB,B,25,0   B 1  1    RT 58
138 SticksPict,StimD,B,0,25   B 1  1    RT 197
139 SticksPict,StimD,A,0,25   B 0  1    RT 51
140 Green_Pict,StimC,A,0,25   A 1  1    RT 102
141 CirclePict,StimA,A,25,0   A 1  1    RT 63
142 Green_Pict,StimC,A,0,25   B 0  0    RT 63
143 CirclePict,StimA,A,25,0   A 1  1    RT 40
144 Worms_Pict,StimB,A,25,0   B 0  1    RT 38
145 Green_Pict,StimC,A,0,25   A 1  1    RT 52
146 Green_Pict,StimC,A,0,25   B 0  0    RT 210
147 Worms_Pict,StimB,B,25,0   B 1  1    RT 41
148 Green_Pict,StimC,A,0,25   A 1  1    RT 45
149 SticksPict,StimD,B,0,25   B 1  1    RT 137
150 SticksPict,StimD,B,0,25   B 1  1    RT 43
151 SticksPict,StimD,B,0,25   B 1  1    RT 58
152 Worms_Pict,StimB,B,25,0   B 1  1    RT 46
153 CirclePict,StimA,B,25,0   A 0  1    RT 37
154 Green_Pict,StimC,A,0,25   A 1  1    RT 127
155 Green_Pict,StimC,B,0,25   A 0  1    RT 48
156 CirclePict,StimA,A,25,0   A 1  1    RT 38
157 Worms_Pict,StimB,B,25,0   B 1  1    RT 41
158 Green_Pict,StimC,A,0,25   A 1  1    RT 142
159 CirclePict,StimA,A,25,0   A 1  1    RT 50
160 SticksPict,StimD,B,0,25   B 1  1    RT 178


Total AB Correct = 61 (76.25% correct)
Total CD Correct = 40 (50% correct)
Total AB optimal = 75 (93.75% optimal)
Total CD optimal = 44 (55% optimal)
Total points = 1025
```
