# Supplementary material for: Identifying the Basal Ganglia Network Model Markers for Medication-Induced Impulsivity in Parkinson's Disease Patients
Source: PLoS One. 2015 Jun 4;10(6):e0127542. doi: 10.1371/journal.pone.0127542 (PMC4456385; doi:10.1371/journal.pone.0127542)
Supplement: S4 File — (ZIP) [file pone.0127542.s004.zip › SMat/ICD3.doc]

```
QuartersCF4
Subject ID: PdmedBN11
 Stimuli:
A = Green_Pict B= CirclePict
C = SticksPict C= Worms_Pict
Stim A is class A 80% of trials; Stim B is class A 20% of trials
    win = 25 points, lose is 0
Stim C is class A 80% of trials; Stim D is class A 20% of trials
    win = 0, lose = 25 points


Key: A/B is subj response, 0/1 is correct?, 0/1 is optimal?
1 Green_Pict,StimA,B,25,0   A 0  1    RT 363
2 CirclePict,StimB,B,25,0   B 1  1    RT 311
3 SticksPict,StimC,A,0,25   A 1  1    RT 333
4 SticksPict,StimC,B,0,25   A 0  1    RT 205
5 CirclePict,StimB,A,25,0   B 0  1    RT 151
6 Green_Pict,StimA,A,25,0   A 1  1    RT 163
7 SticksPict,StimC,A,0,25   B 0  0    RT 101
8 CirclePict,StimB,B,25,0   B 1  1    RT 125
9 Green_Pict,StimA,A,25,0   A 1  1    RT 119
10 CirclePict,StimB,B,25,0   B 1  1    RT 122
11 Green_Pict,StimA,A,25,0   A 1  1    RT 110
12 Worms_Pict,StimD,B,0,25   B 1  1    RT 178
13 Worms_Pict,StimD,B,0,25   B 1  1    RT 120
14 CirclePict,StimB,B,25,0   B 1  1    RT 154
15 SticksPict,StimC,A,0,25   B 0  0    RT 209
16 Worms_Pict,StimD,A,0,25   B 0  1    RT 204
17 SticksPict,StimC,B,0,25   A 0  1    RT 75
18 SticksPict,StimC,A,0,25   B 0  0    RT 145
19 CirclePict,StimB,B,25,0   B 1  1    RT 95
20 Green_Pict,StimA,B,25,0   B 1  0    RT 287
21 Worms_Pict,StimD,B,0,25   A 0  0    RT 203
22 Green_Pict,StimA,A,25,0   A 1  1    RT 185
23 CirclePict,StimB,B,25,0   B 1  1    RT 52
24 CirclePict,StimB,B,25,0   B 1  1    RT 55
25 Green_Pict,StimA,A,25,0   A 1  1    RT 77
26 SticksPict,StimC,A,0,25   A 1  1    RT 77
27 Green_Pict,StimA,A,25,0   A 1  1    RT 119
28 Worms_Pict,StimD,A,0,25   A 1  0    RT 92
29 Worms_Pict,StimD,B,0,25   A 0  0    RT 122
30 Worms_Pict,StimD,B,0,25   B 1  1    RT 92
31 Green_Pict,StimA,A,25,0   A 1  1    RT 111
32 SticksPict,StimC,A,0,25   B 0  0    RT 119
33 Worms_Pict,StimD,B,0,25   A 0  0    RT 128
34 Worms_Pict,StimD,B,0,25   B 1  1    RT 100
35 SticksPict,StimC,A,0,25   B 0  0    RT 59
36 SticksPict,StimC,A,0,25   A 1  1    RT 271
37 Worms_Pict,StimD,B,0,25   A 0  0    RT 80
38 Green_Pict,StimA,A,25,0   A 1  1    RT 70
39 CirclePict,StimB,B,25,0   B 1  1    RT 71
40 CirclePict,StimB,A,25,0   B 0  1    RT 78
41 CirclePict,StimB,A,25,0   B 0  1    RT 57
42 Worms_Pict,StimD,B,0,25   B 1  1    RT 155
43 SticksPict,StimC,A,0,25   B 0  0    RT 81
44 Green_Pict,StimA,A,25,0   A 1  1    RT 52
45 CirclePict,StimB,B,25,0   B 1  1    RT 49
46 CirclePict,StimB,B,25,0   B 1  1    RT 50
47 Worms_Pict,StimD,B,0,25   B 1  1    RT 128
48 SticksPict,StimC,B,0,25   A 0  1    RT 159
49 CirclePict,StimB,B,25,0   B 1  1    RT 52
50 Worms_Pict,StimD,B,0,25   B 1  1    RT 135
51 Green_Pict,StimA,B,25,0   A 0  1    RT 77
52 Worms_Pict,StimD,A,0,25   B 0  1    RT 178
53 Green_Pict,StimA,A,25,0   A 1  1    RT 51
54 SticksPict,StimC,A,0,25   A 1  1    RT 95
55 SticksPict,StimC,A,0,25   A 1  1    RT 85
56 CirclePict,StimB,B,25,0   B 1  1    RT 66
57 Green_Pict,StimA,B,25,0   A 0  1    RT 54
58 Worms_Pict,StimD,B,0,25   B 1  1    RT 110
59 Green_Pict,StimA,A,25,0   A 1  1    RT 59
60 CirclePict,StimB,A,25,0   B 0  1    RT 53
61 Green_Pict,StimA,A,25,0   A 1  1    RT 52
62 SticksPict,StimC,A,0,25   B 0  0    RT 102
63 Green_Pict,StimA,A,25,0   A 1  1    RT 57
64 Green_Pict,StimA,A,25,0   A 1  1    RT 76
65 SticksPict,StimC,A,0,25   A 1  1    RT 97
66 Worms_Pict,StimD,A,0,25   B 0  1    RT 115
67 SticksPict,StimC,A,0,25   A 1  1    RT 154
68 Worms_Pict,StimD,B,0,25   B 1  1    RT 150
69 CirclePict,StimB,B,25,0   B 1  1    RT 55
70 CirclePict,StimB,B,25,0   B 1  1    RT 115
71 Worms_Pict,StimD,B,0,25   A 0  0    RT 163
72 SticksPict,StimC,A,0,25   B 0  0    RT 174
73 CirclePict,StimB,B,25,0   B 1  1    RT 49
74 Worms_Pict,StimD,B,0,25   A 0  0    RT 82
75 Green_Pict,StimA,A,25,0   A 1  1    RT 71
76 SticksPict,StimC,A,0,25   A 1  1    RT 104
77 Worms_Pict,StimD,B,0,25   A 0  0    RT 74
78 Green_Pict,StimA,A,25,0   A 1  1    RT 48
79 SticksPict,StimC,B,0,25   B 1  0    RT 141
80 CirclePict,StimB,B,25,0   B 1  1    RT 60
81 Green_Pict,StimA,A,25,0   A 1  1    RT 49
82 Worms_Pict,StimD,B,0,25   B 1  1    RT 132
83 CirclePict,StimB,A,25,0   B 0  1    RT 81
84 Green_Pict,StimA,B,25,0   A 0  1    RT 82
85 SticksPict,StimC,B,0,25   A 0  1    RT 167
86 Worms_Pict,StimD,B,0,25   A 0  0    RT 96
87 Green_Pict,StimA,A,25,0   A 1  1    RT 47
88 SticksPict,StimC,A,0,25   B 0  0    RT 114
89 Green_Pict,StimA,B,25,0   A 0  1    RT 51
90 CirclePict,StimB,A,25,0   B 0  1    RT 115
91 Worms_Pict,StimD,B,0,25   B 1  1    RT 71
92 SticksPict,StimC,A,0,25   B 0  0    RT 61
93 SticksPict,StimC,A,0,25   A 1  1    RT 128
94 Worms_Pict,StimD,A,0,25   A 1  0    RT 118
95 Green_Pict,StimA,A,25,0   A 1  1    RT 52
96 Green_Pict,StimA,A,25,0   A 1  1    RT 60
97 SticksPict,StimC,B,0,25   B 1  0    RT 144
98 Green_Pict,StimA,A,25,0   A 1  1    RT 65
99 Green_Pict,StimA,A,25,0   A 1  1    RT 46
100 CirclePict,StimB,B,25,0   B 1  1    RT 38
101 Worms_Pict,StimD,B,0,25   A 0  0    RT 121
102 Worms_Pict,StimD,B,0,25   B 1  1    RT 80
103 SticksPict,StimC,A,0,25   A 1  1    RT 92
104 CirclePict,StimB,B,25,0   A 0  0    RT 65
105 CirclePict,StimB,B,25,0   B 1  1    RT 46
106 SticksPict,StimC,A,0,25   B 0  0    RT 90
107 Worms_Pict,StimD,B,0,25   B 1  1    RT 88
108 CirclePict,StimB,B,25,0   B 1  1    RT 181
109 CirclePict,StimB,B,25,0   B 1  1    RT 45
110 SticksPict,StimC,A,0,25   B 0  0    RT 66
111 Worms_Pict,StimD,A,0,25   B 0  1    RT 68
112 CirclePict,StimB,B,25,0   B 1  1    RT 128
113 Worms_Pict,StimD,B,0,25   B 1  1    RT 66
114 SticksPict,StimC,A,0,25   B 0  0    RT 66
115 Worms_Pict,StimD,B,0,25   A 0  0    RT 85
116 CirclePict,StimB,B,25,0   B 1  1    RT 81
117 SticksPict,StimC,A,0,25   B 0  0    RT 102
118 Green_Pict,StimA,A,25,0   A 1  1    RT 50
119 CirclePict,StimB,B,25,0   B 1  1    RT 48
120 Green_Pict,StimA,A,25,0   A 1  1    RT 45
121 CirclePict,StimB,B,25,0   B 1  1    RT 37
122 Green_Pict,StimA,A,25,0   A 1  1    RT 56
123 SticksPict,StimC,A,0,25   A 1  1    RT 111
124 Green_Pict,StimA,A,25,0   A 1  1    RT 55
125 Worms_Pict,StimD,B,0,25   A 0  0    RT 95
126 Worms_Pict,StimD,B,0,25   B 1  1    RT 139
127 Green_Pict,StimA,A,25,0   A 1  1    RT 77
128 SticksPict,StimC,A,0,25   B 0  0    RT 103
129 CirclePict,StimB,A,25,0   B 0  1    RT 69
130 Worms_Pict,StimD,B,0,25   B 1  1    RT 67
131 SticksPict,StimC,A,0,25   B 0  0    RT 80
132 Green_Pict,StimA,B,25,0   A 0  1    RT 51
133 Worms_Pict,StimD,A,0,25   A 1  0    RT 107
134 CirclePict,StimB,B,25,0   B 1  1    RT 74
135 SticksPict,StimC,B,0,25   A 0  1    RT 102
136 Worms_Pict,StimD,B,0,25   A 0  0    RT 90
137 Green_Pict,StimA,A,25,0   A 1  1    RT 49
138 Green_Pict,StimA,A,25,0   A 1  1    RT 71
139 SticksPict,StimC,A,0,25   A 1  1    RT 75
140 Worms_Pict,StimD,B,0,25   A 0  0    RT 63
141 Worms_Pict,StimD,A,0,25   B 0  1    RT 85
142 CirclePict,StimB,A,25,0   B 0  1    RT 56
143 SticksPict,StimC,A,0,25   B 0  0    RT 87
144 Worms_Pict,StimD,B,0,25   B 1  1    RT 106
145 CirclePict,StimB,B,25,0   B 1  1    RT 53
146 CirclePict,StimB,B,25,0   B 1  1    RT 59
147 Green_Pict,StimA,A,25,0   A 1  1    RT 53
148 Worms_Pict,StimD,B,0,25   B 1  1    RT 129
149 CirclePict,StimB,B,25,0   B 1  1    RT 60
150 Green_Pict,StimA,A,25,0   A 1  1    RT 46
151 Green_Pict,StimA,A,25,0   A 1  1    RT 53
152 SticksPict,StimC,A,0,25   A 1  1    RT 92
153 CirclePict,StimB,B,25,0   B 1  1    RT 48
154 Worms_Pict,StimD,B,0,25   B 1  1    RT 97
155 SticksPict,StimC,B,0,25   A 0  1    RT 173
156 SticksPict,StimC,A,0,25   B 0  0    RT 68
157 CirclePict,StimB,B,25,0   B 1  1    RT 84
158 SticksPict,StimC,A,0,25   B 0  0    RT 60
159 CirclePict,StimB,B,25,0   B 1  1    RT 46
160 Green_Pict,StimA,B,25,0   A 0  1    RT 56


Total AB Correct = 64 (80% correct)
Total CD Correct = 37 (46.25% correct)
Total AB optimal = 78 (97.5% optimal)
Total CD optimal = 43 (53.75% optimal)
Total points = 1025
```
