# Supplementary material for: Identifying the Basal Ganglia Network Model Markers for Medication-Induced Impulsivity in Parkinson's Disease Patients
Source: PLoS One. 2015 Jun 4;10(6):e0127542. doi: 10.1371/journal.pone.0127542 (PMC4456385; doi:10.1371/journal.pone.0127542)
Supplement: S4 File — (ZIP) [file pone.0127542.s004.zip › SMat/ICD4.doc]

```
QuartersCF4
Subject ID: PdmedBN10
 Stimuli:
A = SticksPict B= Green_Pict
C = Worms_Pict C= CirclePict
Stim A is class A 80% of trials; Stim B is class A 20% of trials
    win = 25 points, lose is 0
Stim C is class A 80% of trials; Stim D is class A 20% of trials
    win = 0, lose = 25 points


Key: A/B is subj response, 0/1 is correct?, 0/1 is optimal?
1 CirclePict,StimD,A,0,25   A 1  0    RT 59
2 CirclePict,StimD,B,0,25   A 0  0    RT 38
3 Green_Pict,StimB,B,25,0   B 1  1    RT 141
4 SticksPict,StimA,A,25,0   B 0  0    RT 47
5 SticksPict,StimA,A,25,0   B 0  0    RT 50
6 Green_Pict,StimB,A,25,0   B 0  1    RT 51
7 Worms_Pict,StimC,B,0,25   A 0  1    RT 50
8 SticksPict,StimA,A,25,0   A 1  1    RT 154
9 SticksPict,StimA,A,25,0   A 1  1    RT 46
10 Worms_Pict,StimC,A,0,25   B 0  0    RT 65
11 Green_Pict,StimB,B,25,0   B 1  1    RT 68
12 CirclePict,StimD,B,0,25   A 0  0    RT 49
13 Green_Pict,StimB,A,25,0   B 0  1    RT 42
14 Worms_Pict,StimC,A,0,25   A 1  1    RT 66
15 Worms_Pict,StimC,A,0,25   B 0  0    RT 67
16 Worms_Pict,StimC,A,0,25   A 1  1    RT 79
17 SticksPict,StimA,A,25,0   B 0  0    RT 106
18 SticksPict,StimA,A,25,0   B 0  0    RT 38
19 CirclePict,StimD,B,0,25   A 0  0    RT 45
20 Worms_Pict,StimC,A,0,25   B 0  0    RT 65
21 Green_Pict,StimB,B,25,0   A 0  0    RT 47
22 CirclePict,StimD,B,0,25   A 0  0    RT 62
23 Green_Pict,StimB,B,25,0   B 1  1    RT 637
24 Worms_Pict,StimC,A,0,25   A 1  1    RT 53
25 CirclePict,StimD,B,0,25   B 1  1    RT 38
26 CirclePict,StimD,A,0,25   A 1  0    RT 51
27 Green_Pict,StimB,B,25,0   B 1  1    RT 38
28 Green_Pict,StimB,B,25,0   B 1  1    RT 30
29 Green_Pict,StimB,B,25,0   B 1  1    RT 27
30 SticksPict,StimA,A,25,0   B 0  0    RT 84
31 Worms_Pict,StimC,A,0,25   A 1  1    RT 57
32 CirclePict,StimD,B,0,25   B 1  1    RT 59
33 SticksPict,StimA,A,25,0   A 1  1    RT 59
34 Worms_Pict,StimC,A,0,25   B 0  0    RT 40
35 Worms_Pict,StimC,B,0,25   A 0  1    RT 40
36 CirclePict,StimD,B,0,25   A 0  0    RT 48
37 Green_Pict,StimB,B,25,0   B 1  1    RT 41
38 SticksPict,StimA,B,25,0   A 0  1    RT 57
39 CirclePict,StimD,B,0,25   B 1  1    RT 42
40 SticksPict,StimA,B,25,0   B 1  0    RT 67
41 SticksPict,StimA,A,25,0   B 0  0    RT 31
42 Green_Pict,StimB,A,25,0   B 0  1    RT 41
43 SticksPict,StimA,A,25,0   B 0  0    RT 39
44 CirclePict,StimD,B,0,25   A 0  0    RT 29
45 Worms_Pict,StimC,B,0,25   B 1  0    RT 30
46 Worms_Pict,StimC,A,0,25   B 0  0    RT 26
47 Green_Pict,StimB,B,25,0   B 1  1    RT 46
48 Green_Pict,StimB,A,25,0   B 0  1    RT 27
49 SticksPict,StimA,B,25,0   A 0  1    RT 60
50 Green_Pict,StimB,B,25,0   B 1  1    RT 39
51 SticksPict,StimA,A,25,0   A 1  1    RT 60
52 CirclePict,StimD,A,0,25   B 0  1    RT 53
53 SticksPict,StimA,B,25,0   A 0  1    RT 62
54 CirclePict,StimD,B,0,25   B 1  1    RT 38
55 SticksPict,StimA,A,25,0   A 1  1    RT 68
56 Green_Pict,StimB,B,25,0   B 1  1    RT 37
57 SticksPict,StimA,A,25,0   A 1  1    RT 51
58 CirclePict,StimD,A,0,25   B 0  1    RT 47
59 CirclePict,StimD,B,0,25   A 0  0    RT 52
60 Worms_Pict,StimC,A,0,25   A 1  1    RT 46
61 CirclePict,StimD,B,0,25   B 1  1    RT 40
62 Worms_Pict,StimC,A,0,25   A 1  1    RT 30
63 CirclePict,StimD,B,0,25   B 1  1    RT 54
64 Worms_Pict,StimC,B,0,25   A 0  1    RT 38
65 Green_Pict,StimB,B,25,0   B 1  1    RT 34
66 Green_Pict,StimB,B,25,0   B 1  1    RT 28
67 CirclePict,StimD,B,0,25   A 0  0    RT 45
68 Worms_Pict,StimC,A,0,25   B 0  0    RT 43
69 SticksPict,StimA,A,25,0   A 1  1    RT 47
70 Worms_Pict,StimC,A,0,25   A 1  1    RT 78
71 Worms_Pict,StimC,A,0,25   B 0  0    RT 73
72 SticksPict,StimA,A,25,0   A 1  1    RT 44
73 Worms_Pict,StimC,A,0,25   B 0  0    RT 68
74 Green_Pict,StimB,B,25,0   B 1  1    RT 37
75 CirclePict,StimD,B,0,25   B 1  1    RT 40
76 Green_Pict,StimB,B,25,0   B 1  1    RT 40
77 CirclePict,StimD,B,0,25   B 1  1    RT 39
78 SticksPict,StimA,A,25,0   A 1  1    RT 37
79 Green_Pict,StimB,B,25,0   B 1  1    RT 37
80 Worms_Pict,StimC,A,0,25   A 1  1    RT 71
81 SticksPict,StimA,B,25,0   A 0  1    RT 46
82 CirclePict,StimD,B,0,25   B 1  1    RT 40
83 CirclePict,StimD,A,0,25   A 1  0    RT 40
84 SticksPict,StimA,A,25,0   A 1  1    RT 42
85 SticksPict,StimA,A,25,0   A 1  1    RT 28
86 Green_Pict,StimB,B,25,0   B 1  1    RT 42
87 SticksPict,StimA,A,25,0   A 1  1    RT 37
88 Worms_Pict,StimC,A,0,25   B 0  0    RT 43
89 SticksPict,StimA,A,25,0   A 1  1    RT 32
90 SticksPict,StimA,B,25,0   A 0  1    RT 73
91 CirclePict,StimD,A,0,25   B 0  1    RT 59
92 SticksPict,StimA,A,25,0   A 1  1    RT 28
93 Worms_Pict,StimC,A,0,25   B 0  0    RT 54
94 SticksPict,StimA,A,25,0   A 1  1    RT 33
95 Green_Pict,StimB,B,25,0   B 1  1    RT 38
96 SticksPict,StimA,A,25,0   A 1  1    RT 32
97 Worms_Pict,StimC,B,0,25   A 0  1    RT 63
98 CirclePict,StimD,B,0,25   B 1  1    RT 31
99 Green_Pict,StimB,B,25,0   B 1  1    RT 35
100 Green_Pict,StimB,A,25,0   B 0  1    RT 31
101 CirclePict,StimD,B,0,25   A 0  0    RT 39
102 Worms_Pict,StimC,A,0,25   A 1  1    RT 71
103 Green_Pict,StimB,B,25,0   A 0  0    RT 37
104 Worms_Pict,StimC,B,0,25   B 1  0    RT 85
105 Worms_Pict,StimC,A,0,25   A 1  1    RT 56
106 Green_Pict,StimB,B,25,0   A 0  0    RT 86
107 Green_Pict,StimB,B,25,0   B 1  1    RT 27
108 CirclePict,StimD,B,0,25   B 1  1    RT 87
109 Worms_Pict,StimC,A,0,25   A 1  1    RT 85
110 Green_Pict,StimB,B,25,0   B 1  1    RT 30
111 Worms_Pict,StimC,A,0,25   A 1  1    RT 41
112 Worms_Pict,StimC,A,0,25   B 0  0    RT 23
113 CirclePict,StimD,B,0,25   A 0  0    RT 20
114 CirclePict,StimD,B,0,25   B 1  1    RT 25
115 SticksPict,StimA,A,25,0   A 1  1    RT 34
116 Green_Pict,StimB,A,25,0   B 0  1    RT 43
117 Worms_Pict,StimC,A,0,25   A 1  1    RT 38
118 CirclePict,StimD,B,0,25   B 1  1    RT 32
119 Green_Pict,StimB,B,25,0   B 1  1    RT 40
120 CirclePict,StimD,B,0,25   A 0  0    RT 34
121 CirclePict,StimD,A,0,25   B 0  1    RT 47
122 Green_Pict,StimB,A,25,0   B 0  1    RT 28
123 Green_Pict,StimB,B,25,0   B 1  1    RT 26
124 CirclePict,StimD,B,0,25   A 0  0    RT 61
125 SticksPict,StimA,A,25,0   A 1  1    RT 73
126 CirclePict,StimD,B,0,25   B 1  1    RT 36
127 CirclePict,StimD,B,0,25   A 0  0    RT 80
128 Green_Pict,StimB,A,25,0   B 0  1    RT 30
129 CirclePict,StimD,B,0,25   B 1  1    RT 41
130 SticksPict,StimA,B,25,0   A 0  1    RT 38
131 Green_Pict,StimB,B,25,0   B 1  1    RT 31
132 Worms_Pict,StimC,A,0,25   A 1  1    RT 40
133 SticksPict,StimA,B,25,0   A 0  1    RT 47
134 SticksPict,StimA,A,25,0   B 0  0    RT 44
135 CirclePict,StimD,B,0,25   B 1  1    RT 94
136 Green_Pict,StimB,B,25,0   B 1  1    RT 36
137 Worms_Pict,StimC,B,0,25   A 0  1    RT 40
138 Worms_Pict,StimC,A,0,25   B 0  0    RT 41
139 Worms_Pict,StimC,A,0,25   A 1  1    RT 41
140 CirclePict,StimD,B,0,25   B 1  1    RT 28
141 CirclePict,StimD,B,0,25   A 0  0    RT 49
142 Green_Pict,StimB,B,25,0   B 1  1    RT 30
143 SticksPict,StimA,A,25,0   B 0  0    RT 31
144 SticksPict,StimA,A,25,0   A 1  1    RT 49
145 CirclePict,StimD,A,0,25   B 0  1    RT 40
146 Worms_Pict,StimC,A,0,25   A 1  1    RT 22
147 Green_Pict,StimB,B,25,0   B 1  1    RT 31
148 Green_Pict,StimB,B,25,0   B 1  1    RT 38
149 Worms_Pict,StimC,A,0,25   B 0  0    RT 18
150 SticksPict,StimA,A,25,0   A 1  1    RT 37
151 CirclePict,StimD,B,0,25   B 1  1    RT 33
152 SticksPict,StimA,A,25,0   A 1  1    RT 37
153 SticksPict,StimA,A,25,0   A 1  1    RT 35
154 Green_Pict,StimB,B,25,0   B 1  1    RT 34
155 Worms_Pict,StimC,A,0,25   A 1  1    RT 63
156 SticksPict,StimA,A,25,0   A 1  1    RT 103
157 Green_Pict,StimB,B,25,0   B 1  1    RT 158
158 Worms_Pict,StimC,B,0,25   A 0  1    RT 45
159 Worms_Pict,StimC,A,0,25   B 0  0    RT 48
160 Worms_Pict,StimC,A,0,25   A 1  1    RT 42


Total AB Correct = 53 (66.25% correct)
Total CD Correct = 41 (51.25% correct)
Total AB optimal = 67 (83.75% optimal)
Total CD optimal = 47 (58.75% optimal)
Total points = 850
```
