# Supplementary material for: Identifying the Basal Ganglia Network Model Markers for Medication-Induced Impulsivity in Parkinson's Disease Patients
Source: PLoS One. 2015 Jun 4;10(6):e0127542. doi: 10.1371/journal.pone.0127542 (PMC4456385; doi:10.1371/journal.pone.0127542)
Supplement: S4 File — (ZIP) [file pone.0127542.s004.zip › SMat/ICDdemographics.doc]

Clinical and demographic characteristics. Participants are medicated PD patients who were followed-up and remained on the same medication.

|  | ICD (n=4) | non-ICD (n=4) |
| --- | --- | --- |
| Age (years) | 42.8 (7.2) | 42.3 (7.8) |
| Education (years) | 14.5 (6.4) | 14.3 (6.8) |
| Months since diagnosis | 4.2 (2.2) | 4.6 (2.8) |
| Dopamine agonist medication | Pramipexole (n=4):  4.8 mg/day | Pramipexole (n=1):  5.0 mg/day  Ropinirole (n=3):  5.8 mg/day |
| Full-scale IQ (WAIS-R) | 109.4 (10.3) | 109.2 (11.0) |
| Socioeconomic status  (Hollingshead) | 35.9 (18.3) | 36.7 (19.5) |
| No. of patients in Hoehn-Yahr Stage | 1.5: 3  2: 1 | 1.5: 3  2: 1 |
| UPDRS | 31.7 (7.9) | 30.6 (8.2) |
| HAM-D | 4.1 (1.6) | 4.1 (2.0) |
| HAM-A | 4.0 (1.9) | 3.9 (1.7) |

Data are mean (standard deviation). PD – Parkinson’s disease, UPDRS – Unified Parkinson’s Disease Rating Scale, HAM-D – Hamilton Depression Scale, HAM-A – Hamilton Anxiety Scale, WAIS-R - Wechsler Adult Intelligence Scale, revised
