# Supplementary material for: Identifying the Basal Ganglia Network Model Markers for Medication-Induced Impulsivity in Parkinson's Disease Patients
Source: PLoS One. 2015 Jun 4;10(6):e0127542. doi: 10.1371/journal.pone.0127542 (PMC4456385; doi:10.1371/journal.pone.0127542)
Supplement: S4 File — (ZIP) [file pone.0127542.s004.zip › SMat/nonICD1.doc]

```
QuartersCF4
Subject ID: PddenovoBN14
 Stimuli:
A = SticksPict B= Green_Pict
C = CirclePict C= Worms_Pict
Stim A is class A 80% of trials; Stim B is class A 20% of trials
    win = 25 points, lose is 0
Stim C is class A 80% of trials; Stim D is class A 20% of trials
    win = 0, lose = 25 points


Key: A/B is subj response, 0/1 is correct?, 0/1 is optimal?
1 SticksPict,StimA,A,25,0   B 0  0    RT 114
2 Green_Pict,StimB,B,25,0   B 1  1    RT 106
3 SticksPict,StimA,A,25,0   B 0  0    RT 104
4 Green_Pict,StimB,B,25,0   B 1  1    RT 52
5 SticksPict,StimA,A,25,0   B 0  0    RT 29
6 Worms_Pict,StimD,B,0,25   B 1  1    RT 333
7 Green_Pict,StimB,B,25,0   B 1  1    RT 34
8 SticksPict,StimA,A,25,0   B 0  0    RT 55
9 Green_Pict,StimB,B,25,0   B 1  1    RT 34
10 CirclePict,StimC,A,0,25   B 0  0    RT 135
11 Green_Pict,StimB,B,25,0   B 1  1    RT 99
12 Green_Pict,StimB,B,25,0   B 1  1    RT 88
13 SticksPict,StimA,B,25,0   B 1  0    RT 65
14 SticksPict,StimA,A,25,0   B 0  0    RT 106
15 Green_Pict,StimB,B,25,0   B 1  1    RT 40
16 SticksPict,StimA,A,25,0   B 0  0    RT 88
17 CirclePict,StimC,A,0,25   B 0  0    RT 79
18 CirclePict,StimC,A,0,25   A 1  1    RT 43
19 Worms_Pict,StimD,A,0,25   B 0  1    RT 54
20 CirclePict,StimC,A,0,25   A 1  1    RT 108
21 SticksPict,StimA,A,25,0   B 0  0    RT 76
22 CirclePict,StimC,A,0,25   A 1  1    RT 55
23 Worms_Pict,StimD,B,0,25   B 1  1    RT 120
24 Worms_Pict,StimD,B,0,25   B 1  1    RT 52
25 Worms_Pict,StimD,B,0,25   B 1  1    RT 56
26 SticksPict,StimA,B,25,0   B 1  0    RT 91
27 Green_Pict,StimB,A,25,0   B 0  1    RT 45
28 CirclePict,StimC,A,0,25   A 1  1    RT 86
29 Worms_Pict,StimD,B,0,25   B 1  1    RT 94
30 Green_Pict,StimB,B,25,0   B 1  1    RT 44
31 Worms_Pict,StimD,A,0,25   A 1  0    RT 126
32 Worms_Pict,StimD,B,0,25   A 0  0    RT 77
33 CirclePict,StimC,A,0,25   A 1  1    RT 77
34 Worms_Pict,StimD,B,0,25   B 1  1    RT 87
35 CirclePict,StimC,B,0,25   A 0  1    RT 78
36 CirclePict,StimC,A,0,25   B 0  0    RT 70
37 CirclePict,StimC,B,0,25   A 0  1    RT 124
38 SticksPict,StimA,A,25,0   B 0  0    RT 51
39 Green_Pict,StimB,A,25,0   B 0  1    RT 135
40 Worms_Pict,StimD,B,0,25   A 0  0    RT 93
41 Green_Pict,StimB,B,25,0   B 1  1    RT 39
42 SticksPict,StimA,A,25,0   B 0  0    RT 71
43 Green_Pict,StimB,B,25,0   B 1  1    RT 78
44 Green_Pict,StimB,B,25,0   B 1  1    RT 91
45 Green_Pict,StimB,B,25,0   B 1  1    RT 30
46 Green_Pict,StimB,B,25,0   B 1  1    RT 33
47 CirclePict,StimC,A,0,25   B 0  0    RT 108
48 CirclePict,StimC,A,0,25   A 1  1    RT 57
49 Green_Pict,StimB,B,25,0   B 1  1    RT 100
50 SticksPict,StimA,B,25,0   A 0  1    RT 47
51 Worms_Pict,StimD,A,0,25   B 0  1    RT 77
52 CirclePict,StimC,A,0,25   B 0  0    RT 90
53 SticksPict,StimA,A,25,0   A 1  1    RT 40
54 Worms_Pict,StimD,B,0,25   A 0  0    RT 111
55 Green_Pict,StimB,A,25,0   B 0  1    RT 40
56 Worms_Pict,StimD,B,0,25   B 1  1    RT 64
57 Worms_Pict,StimD,B,0,25   B 1  1    RT 58
58 Worms_Pict,StimD,A,0,25   B 0  1    RT 68
59 CirclePict,StimC,A,0,25   B 0  0    RT 80
60 CirclePict,StimC,B,0,25   A 0  1    RT 60
61 Worms_Pict,StimD,B,0,25   B 1  1    RT 55
62 Green_Pict,StimB,B,25,0   B 1  1    RT 51
63 CirclePict,StimC,A,0,25   A 1  1    RT 74
64 CirclePict,StimC,B,0,25   A 0  1    RT 73
65 SticksPict,StimA,A,25,0   B 0  0    RT 48
66 SticksPict,StimA,A,25,0   B 0  0    RT 59
67 CirclePict,StimC,A,0,25   A 1  1    RT 87
68 SticksPict,StimA,A,25,0   B 0  0    RT 61
69 Worms_Pict,StimD,B,0,25   B 1  1    RT 65
70 Worms_Pict,StimD,B,0,25   B 1  1    RT 59
71 SticksPict,StimA,A,25,0   A 1  1    RT 48
72 Green_Pict,StimB,A,25,0   B 0  1    RT 87
73 SticksPict,StimA,B,25,0   B 1  0    RT 53
74 SticksPict,StimA,A,25,0   B 0  0    RT 42
75 Worms_Pict,StimD,B,0,25   B 1  1    RT 49
76 Worms_Pict,StimD,B,0,25   B 1  1    RT 140
77 Green_Pict,StimB,B,25,0   B 1  1    RT 39
78 SticksPict,StimA,A,25,0   B 0  0    RT 57
79 CirclePict,StimC,A,0,25   A 1  1    RT 55
80 CirclePict,StimC,A,0,25   A 1  1    RT 35
81 CirclePict,StimC,A,0,25   A 1  1    RT 24
82 Worms_Pict,StimD,B,0,25   B 1  1    RT 41
83 Green_Pict,StimB,B,25,0   B 1  1    RT 43
84 SticksPict,StimA,A,25,0   B 0  0    RT 47
85 Worms_Pict,StimD,B,0,25   B 1  1    RT 41
86 Worms_Pict,StimD,A,0,25   B 0  1    RT 48
87 CirclePict,StimC,A,0,25   A 1  1    RT 151
88 SticksPict,StimA,A,25,0   B 0  0    RT 48
89 SticksPict,StimA,A,25,0   B 0  0    RT 40
90 CirclePict,StimC,A,0,25   A 1  1    RT 74
91 Green_Pict,StimB,B,25,0   B 1  1    RT 50
92 CirclePict,StimC,B,0,25   B 1  0    RT 59
93 CirclePict,StimC,A,0,25   B 0  0    RT 73
94 Green_Pict,StimB,A,25,0   B 0  1    RT 44
95 Green_Pict,StimB,B,25,0   B 1  1    RT 57
96 Worms_Pict,StimD,B,0,25   B 1  1    RT 59
97 Worms_Pict,StimD,A,0,25   B 0  1    RT 71
98 Green_Pict,StimB,B,25,0   B 1  1    RT 53
99 CirclePict,StimC,A,0,25   B 0  0    RT 66
100 CirclePict,StimC,B,0,25   A 0  1    RT 34
101 Green_Pict,StimB,B,25,0   B 1  1    RT 48
102 Green_Pict,StimB,A,25,0   B 0  1    RT 38
103 Worms_Pict,StimD,B,0,25   B 1  1    RT 68
104 SticksPict,StimA,B,25,0   B 1  0    RT 60
105 Green_Pict,StimB,B,25,0   B 1  1    RT 55
106 CirclePict,StimC,A,0,25   A 1  1    RT 56
107 SticksPict,StimA,A,25,0   B 0  0    RT 41
108 Worms_Pict,StimD,B,0,25   B 1  1    RT 43
109 SticksPict,StimA,A,25,0   B 0  0    RT 41
110 SticksPict,StimA,A,25,0   B 0  0    RT 42
111 Worms_Pict,StimD,B,0,25   B 1  1    RT 58
112 SticksPict,StimA,B,25,0   B 1  0    RT 44
113 CirclePict,StimC,A,0,25   A 1  1    RT 45
114 Green_Pict,StimB,B,25,0   B 1  1    RT 31
115 Green_Pict,StimB,B,25,0   B 1  1    RT 42
116 Worms_Pict,StimD,B,0,25   B 1  1    RT 50
117 SticksPict,StimA,A,25,0   B 0  0    RT 35
118 SticksPict,StimA,A,25,0   B 0  0    RT 29
119 Worms_Pict,StimD,B,0,25   B 1  1    RT 64
120 CirclePict,StimC,A,0,25   A 1  1    RT 47
121 SticksPict,StimA,B,25,0   B 1  0    RT 34
122 CirclePict,StimC,B,0,25   A 0  1    RT 57
123 SticksPict,StimA,B,25,0   B 1  0    RT 41
124 CirclePict,StimC,A,0,25   B 0  0    RT 33
125 Worms_Pict,StimD,B,0,25   B 1  1    RT 89
126 SticksPict,StimA,A,25,0   B 0  0    RT 62
127 Worms_Pict,StimD,B,0,25   B 1  1    RT 55
128 SticksPict,StimA,A,25,0   B 0  0    RT 42
129 Green_Pict,StimB,B,25,0   B 1  1    RT 93
130 SticksPict,StimA,A,25,0   B 0  0    RT 41
131 CirclePict,StimC,A,0,25   B 0  0    RT 35
132 SticksPict,StimA,A,25,0   B 0  0    RT 51
133 Green_Pict,StimB,B,25,0   B 1  1    RT 38
134 SticksPict,StimA,A,25,0   B 0  0    RT 137
135 Worms_Pict,StimD,B,0,25   B 1  1    RT 52
136 Green_Pict,StimB,B,25,0   B 1  1    RT 42
137 CirclePict,StimC,A,0,25   A 1  1    RT 63
138 CirclePict,StimC,A,0,25   A 1  1    RT 51
139 Worms_Pict,StimD,B,0,25   B 1  1    RT 38
140 Green_Pict,StimB,B,25,0   B 1  1    RT 40
141 CirclePict,StimC,A,0,25   A 1  1    RT 57
142 Green_Pict,StimB,A,25,0   B 0  1    RT 30
143 Worms_Pict,StimD,A,0,25   A 1  0    RT 44
144 Worms_Pict,StimD,B,0,25   B 1  1    RT 74
145 Green_Pict,StimB,B,25,0   B 1  1    RT 33
146 SticksPict,StimA,A,25,0   A 1  1    RT 37
147 Worms_Pict,StimD,B,0,25   B 1  1    RT 34
148 Green_Pict,StimB,A,25,0   B 0  1    RT 78
149 Worms_Pict,StimD,B,0,25   B 1  1    RT 36
150 SticksPict,StimA,A,25,0   B 0  0    RT 47
151 SticksPict,StimA,A,25,0   B 0  0    RT 55
152 Green_Pict,StimB,B,25,0   B 1  1    RT 44
153 CirclePict,StimC,A,0,25   B 0  0    RT 36
154 CirclePict,StimC,A,0,25   A 1  1    RT 29
155 Worms_Pict,StimD,B,0,25   B 1  1    RT 39
156 Worms_Pict,StimD,A,0,25   B 0  1    RT 39
157 Green_Pict,StimB,B,25,0   B 1  1    RT 42
158 Green_Pict,StimB,B,25,0   B 1  1    RT 37
159 CirclePict,StimC,A,0,25   A 1  1    RT 60
160 CirclePict,StimC,B,0,25   A 0  1    RT 28


Total AB Correct = 42 (52.5% correct)
Total CD Correct = 53 (66.25% correct)
Total AB optimal = 44 (55% optimal)
Total CD optimal = 63 (78.75% optimal)
Total points = 875
```
