# Supplementary material for: Identifying the Basal Ganglia Network Model Markers for Medication-Induced Impulsivity in Parkinson's Disease Patients
Source: PLoS One. 2015 Jun 4;10(6):e0127542. doi: 10.1371/journal.pone.0127542 (PMC4456385; doi:10.1371/journal.pone.0127542)
Supplement: S4 File — (ZIP) [file pone.0127542.s004.zip › SMat/nonICD2.doc]

```
QuartersCF4
Subject ID: PdmedBN1
 Stimuli:
A = CirclePict B= SticksPict
C = Worms_Pict C= Green_Pict
Stim A is class A 80% of trials; Stim B is class A 20% of trials
    win = 25 points, lose is 0
Stim C is class A 80% of trials; Stim D is class A 20% of trials
    win = 0, lose = 25 points


Key: A/B is subj response, 0/1 is correct?, 0/1 is optimal?
1 CirclePict,StimA,A,25,0   A 1  1    RT 330
2 CirclePict,StimA,A,25,0   B 0  0    RT 404
3 Worms_Pict,StimC,A,0,25   A 1  1    RT 312
4 Worms_Pict,StimC,A,0,25   B 0  0    RT 157
5 CirclePict,StimA,A,25,0   B 0  0    RT 239
6 Green_Pict,StimD,A,0,25   A 1  0    RT 158
7 CirclePict,StimA,A,25,0   B 0  0    RT 138
8 SticksPict,StimB,A,25,0   A 1  0    RT 413
9 SticksPict,StimB,B,25,0   B 1  1    RT 116
10 Green_Pict,StimD,B,0,25   B 1  1    RT 354
11 CirclePict,StimA,A,25,0   B 0  0    RT 116
12 SticksPict,StimB,B,25,0   B 1  1    RT 114
13 CirclePict,StimA,A,25,0   B 0  0    RT 86
14 SticksPict,StimB,A,25,0   B 0  1    RT 113
15 Green_Pict,StimD,B,0,25   A 0  0    RT 142
16 Worms_Pict,StimC,A,0,25   B 0  0    RT 449
17 SticksPict,StimB,B,25,0   B 1  1    RT 85
18 Green_Pict,StimD,B,0,25   B 1  1    RT 220
19 Green_Pict,StimD,B,0,25   B 1  1    RT 267
20 SticksPict,StimB,B,25,0   B 1  1    RT 167
21 Worms_Pict,StimC,A,0,25   A 1  1    RT 206
22 CirclePict,StimA,A,25,0   B 0  0    RT 69
23 Worms_Pict,StimC,B,0,25   A 0  1    RT 206
24 Green_Pict,StimD,A,0,25   B 0  1    RT 73
25 Worms_Pict,StimC,A,0,25   B 0  0    RT 148
26 CirclePict,StimA,B,25,0   B 1  0    RT 70
27 SticksPict,StimB,B,25,0   B 1  1    RT 108
28 SticksPict,StimB,B,25,0   B 1  1    RT 256
29 CirclePict,StimA,A,25,0   A 1  1    RT 190
30 Green_Pict,StimD,B,0,25   B 1  1    RT 136
31 CirclePict,StimA,B,25,0   A 0  1    RT 112
32 Worms_Pict,StimC,B,0,25   A 0  1    RT 108
33 SticksPict,StimB,B,25,0   B 1  1    RT 359
34 Green_Pict,StimD,B,0,25   B 1  1    RT 232
35 Worms_Pict,StimC,A,0,25   A 1  1    RT 84
36 Worms_Pict,StimC,A,0,25   A 1  1    RT 307
37 Green_Pict,StimD,B,0,25   B 1  1    RT 361
38 SticksPict,StimB,B,25,0   B 1  1    RT 87
39 Worms_Pict,StimC,A,0,25   A 1  1    RT 97
40 Green_Pict,StimD,B,0,25   B 1  1    RT 893
41 Green_Pict,StimD,B,0,25   B 1  1    RT 104
42 CirclePict,StimA,B,25,0   A 0  1    RT 178
43 CirclePict,StimA,A,25,0   A 1  1    RT 122
44 SticksPict,StimB,B,25,0   B 1  1    RT 74
45 SticksPict,StimB,B,25,0   B 1  1    RT 93
46 Green_Pict,StimD,B,0,25   A 0  0    RT 357
47 CirclePict,StimA,A,25,0   A 1  1    RT 169
48 SticksPict,StimB,A,25,0   B 0  1    RT 99
49 Worms_Pict,StimC,A,0,25   A 1  1    RT 77
50 CirclePict,StimA,A,25,0   A 1  1    RT 89
51 Worms_Pict,StimC,A,0,25   B 0  0    RT 205
52 Green_Pict,StimD,A,0,25   B 0  1    RT 62
53 Green_Pict,StimD,B,0,25   A 0  0    RT 90
54 SticksPict,StimB,B,25,0   B 1  1    RT 65
55 CirclePict,StimA,A,25,0   A 1  1    RT 75
56 Green_Pict,StimD,B,0,25   A 0  0    RT 78
57 CirclePict,StimA,A,25,0   A 1  1    RT 171
58 CirclePict,StimA,A,25,0   A 1  1    RT 74
59 Worms_Pict,StimC,B,0,25   A 0  1    RT 173
60 Worms_Pict,StimC,A,0,25   B 0  0    RT 146
61 Worms_Pict,StimC,A,0,25   B 0  0    RT 79
62 Worms_Pict,StimC,A,0,25   A 1  1    RT 106
63 SticksPict,StimB,B,25,0   B 1  1    RT 66
64 SticksPict,StimB,B,25,0   B 1  1    RT 178
65 CirclePict,StimA,A,25,0   A 1  1    RT 93
66 Green_Pict,StimD,B,0,25   A 0  0    RT 96
67 CirclePict,StimA,A,25,0   A 1  1    RT 67
68 Green_Pict,StimD,B,0,25   A 0  0    RT 123
69 Worms_Pict,StimC,A,0,25   A 1  1    RT 201
70 SticksPict,StimB,A,25,0   B 0  1    RT 67
71 Green_Pict,StimD,A,0,25   A 1  0    RT 265
72 SticksPict,StimB,B,25,0   B 1  1    RT 65
73 Worms_Pict,StimC,B,0,25   A 0  1    RT 213
74 Green_Pict,StimD,B,0,25   A 0  0    RT 93
75 Worms_Pict,StimC,A,0,25   A 1  1    RT 128
76 Worms_Pict,StimC,A,0,25   B 0  0    RT 158
77 SticksPict,StimB,B,25,0   B 1  1    RT 53
78 CirclePict,StimA,B,25,0   A 0  1    RT 60
79 Green_Pict,StimD,B,0,25   A 0  0    RT 292
80 SticksPict,StimB,B,25,0   B 1  1    RT 258
81 Green_Pict,StimD,B,0,25   B 1  1    RT 108
82 CirclePict,StimA,B,25,0   A 0  1    RT 127
83 CirclePict,StimA,A,25,0   A 1  1    RT 102
84 CirclePict,StimA,A,25,0   A 1  1    RT 88
85 Green_Pict,StimD,A,0,25   A 1  0    RT 69
86 Green_Pict,StimD,A,0,25   A 1  0    RT 211
87 Worms_Pict,StimC,A,0,25   A 1  1    RT 137
88 CirclePict,StimA,B,25,0   A 0  1    RT 60
89 Green_Pict,StimD,B,0,25   A 0  0    RT 273
90 SticksPict,StimB,B,25,0   B 1  1    RT 81
91 SticksPict,StimB,B,25,0   B 1  1    RT 119
92 SticksPict,StimB,B,25,0   B 1  1    RT 103
93 Worms_Pict,StimC,A,0,25   B 0  0    RT 149
94 CirclePict,StimA,A,25,0   A 1  1    RT 48
95 CirclePict,StimA,A,25,0   A 1  1    RT 58
96 CirclePict,StimA,A,25,0   A 1  1    RT 51
97 CirclePict,StimA,A,25,0   A 1  1    RT 73
98 SticksPict,StimB,B,25,0   B 1  1    RT 106
99 SticksPict,StimB,A,25,0   B 0  1    RT 65
100 SticksPict,StimB,B,25,0   B 1  1    RT 172
101 Worms_Pict,StimC,A,0,25   B 0  0    RT 200
102 Green_Pict,StimD,B,0,25   B 1  1    RT 197
103 Green_Pict,StimD,B,0,25   B 1  1    RT 131
104 Green_Pict,StimD,B,0,25   B 1  1    RT 75
105 CirclePict,StimA,A,25,0   A 1  1    RT 72
106 Green_Pict,StimD,B,0,25   B 1  1    RT 89
107 CirclePict,StimA,A,25,0   A 1  1    RT 51
108 Green_Pict,StimD,B,0,25   B 1  1    RT 60
109 Worms_Pict,StimC,B,0,25   B 1  0    RT 242
110 Worms_Pict,StimC,A,0,25   B 0  0    RT 101
111 Worms_Pict,StimC,A,0,25   A 1  1    RT 198
112 SticksPict,StimB,B,25,0   B 1  1    RT 197
113 Worms_Pict,StimC,B,0,25   A 0  1    RT 188
114 Worms_Pict,StimC,A,0,25   B 0  0    RT 127
115 Worms_Pict,StimC,A,0,25   A 1  1    RT 218
116 SticksPict,StimB,A,25,0   B 0  1    RT 89
117 Green_Pict,StimD,B,0,25   B 1  1    RT 196
118 Worms_Pict,StimC,A,0,25   B 0  0    RT 136
119 SticksPict,StimB,B,25,0   B 1  1    RT 191
120 SticksPict,StimB,B,25,0   B 1  1    RT 158
121 SticksPict,StimB,B,25,0   B 1  1    RT 82
122 SticksPict,StimB,B,25,0   B 1  1    RT 62
123 CirclePict,StimA,B,25,0   A 0  1    RT 63
124 Worms_Pict,StimC,A,0,25   B 0  0    RT 182
125 SticksPict,StimB,B,25,0   B 1  1    RT 361
126 Worms_Pict,StimC,A,0,25   A 1  1    RT 64
127 Worms_Pict,StimC,B,0,25   A 0  1    RT 111
128 Worms_Pict,StimC,A,0,25   B 0  0    RT 104
129 Worms_Pict,StimC,A,0,25   A 1  1    RT 209
130 Green_Pict,StimD,A,0,25   B 0  1    RT 344
131 CirclePict,StimA,A,25,0   A 1  1    RT 76
132 SticksPict,StimB,A,25,0   A 1  0    RT 451
133 Worms_Pict,StimC,A,0,25   A 1  1    RT 149
134 SticksPict,StimB,B,25,0   A 0  0    RT 207
135 CirclePict,StimA,A,25,0   A 1  1    RT 57
136 SticksPict,StimB,B,25,0   A 0  0    RT 223
137 Green_Pict,StimD,B,0,25   B 1  1    RT 82
138 Green_Pict,StimD,B,0,25   B 1  1    RT 93
139 SticksPict,StimB,A,25,0   A 1  0    RT 149
140 CirclePict,StimA,A,25,0   A 1  1    RT 80
141 SticksPict,StimB,B,25,0   B 1  1    RT 179
142 Green_Pict,StimD,B,0,25   B 1  1    RT 180
143 SticksPict,StimB,B,25,0   B 1  1    RT 195
144 Green_Pict,StimD,B,0,25   B 1  1    RT 86
145 CirclePict,StimA,A,25,0   A 1  1    RT 62
146 CirclePict,StimA,A,25,0   A 1  1    RT 73
147 Green_Pict,StimD,A,0,25   B 0  1    RT 150
148 CirclePict,StimA,B,25,0   A 0  1    RT 53
149 Green_Pict,StimD,B,0,25   A 0  0    RT 170
150 Green_Pict,StimD,B,0,25   B 1  1    RT 140
151 Worms_Pict,StimC,A,0,25   A 1  1    RT 148
152 CirclePict,StimA,A,25,0   A 1  1    RT 125
153 Green_Pict,StimD,B,0,25   B 1  1    RT 233
154 Worms_Pict,StimC,A,0,25   A 1  1    RT 150
155 CirclePict,StimA,A,25,0   A 1  1    RT 52
156 SticksPict,StimB,B,25,0   B 1  1    RT 223
157 CirclePict,StimA,A,25,0   A 1  1    RT 108
158 Green_Pict,StimD,B,0,25   B 1  1    RT 287
159 Worms_Pict,StimC,B,0,25   A 0  1    RT 267
160 Worms_Pict,StimC,A,0,25   B 0  0    RT 105


Total AB Correct = 60 (75% correct)
Total CD Correct = 44 (55% correct)
Total AB optimal = 68 (85% optimal)
Total CD optimal = 50 (62.5% optimal)
Total points = 1100
```
