# Supplementary material for: Identifying the Basal Ganglia Network Model Markers for Medication-Induced Impulsivity in Parkinson's Disease Patients
Source: PLoS One. 2015 Jun 4;10(6):e0127542. doi: 10.1371/journal.pone.0127542 (PMC4456385; doi:10.1371/journal.pone.0127542)
Supplement: S4 File — (ZIP) [file pone.0127542.s004.zip › SMat/nonICD5.doc]

```
QuartersCF4
Subject ID: PdmedBN2
 Tuesday, February 3, 2007
Time: 8:59:07 AM
Stimuli:
A = Green_Pict B= SticksPict
C = Worms_Pict C= CirclePict
Stim A is class A 80% of trials; Stim B is class A 20% of trials
    win = 25 points, lose is 0
Stim C is class A 80% of trials; Stim D is class A 20% of trials
    win = 0, lose = 25 points


Key: A/B is subj response, 0/1 is correct?, 0/1 is optimal?
1 CirclePict,StimD,B,0,25   A 0  0    RT 852
2 SticksPict,StimB,B,25,0   A 0  0    RT 983
3 CirclePict,StimD,B,0,25   B 1  1    RT 643
4 Green_Pict,StimA,A,25,0   A 1  1    RT 294
5 CirclePict,StimD,A,0,25   A 1  0    RT 328
6 SticksPict,StimB,B,25,0   B 1  1    RT 296
7 Green_Pict,StimA,A,25,0   B 0  0    RT 245
8 CirclePict,StimD,A,0,25   A 1  0    RT 134
9 SticksPict,StimB,B,25,0   B 1  1    RT 229
10 Green_Pict,StimA,A,25,0   B 0  0    RT 237
11 Green_Pict,StimA,A,25,0   A 1  1    RT 355
12 CirclePict,StimD,B,0,25   A 0  0    RT 85
13 Worms_Pict,StimC,A,0,25   B 0  0    RT 222
14 Worms_Pict,StimC,A,0,25   B 0  0    RT 243
15 Worms_Pict,StimC,A,0,25   A 1  1    RT 80
16 SticksPict,StimB,B,25,0   A 0  0    RT 109
17 SticksPict,StimB,B,25,0   B 1  1    RT 248
18 Worms_Pict,StimC,A,0,25   A 1  1    RT 106
19 Worms_Pict,StimC,A,0,25   B 0  0    RT 167
20 CirclePict,StimD,B,0,25   A 0  0    RT 133
21 Green_Pict,StimA,A,25,0   A 1  1    RT 102
22 Green_Pict,StimA,A,25,0   A 1  1    RT 240
23 CirclePict,StimD,B,0,25   A 0  0    RT 77
24 SticksPict,StimB,A,25,0   B 0  1    RT 161
25 Green_Pict,StimA,B,25,0   A 0  1    RT 107
26 CirclePict,StimD,B,0,25   A 0  0    RT 87
27 Worms_Pict,StimC,A,0,25   B 0  0    RT 103
28 CirclePict,StimD,B,0,25   A 0  0    RT 236
29 SticksPict,StimB,A,25,0   B 0  1    RT 148
30 Worms_Pict,StimC,B,0,25   A 0  1    RT 100
31 SticksPict,StimB,B,25,0   A 0  0    RT 151
32 SticksPict,StimB,B,25,0   A 0  0    RT 221
33 SticksPict,StimB,B,25,0   B 1  1    RT 136
34 Worms_Pict,StimC,B,0,25   A 0  1    RT 147
35 Green_Pict,StimA,B,25,0   A 0  1    RT 135
36 CirclePict,StimD,B,0,25   A 0  0    RT 103
37 Green_Pict,StimA,A,25,0   B 0  0    RT 170
38 Worms_Pict,StimC,A,0,25   A 1  1    RT 179
39 Green_Pict,StimA,A,25,0   A 1  1    RT 155
40 Worms_Pict,StimC,A,0,25   A 1  1    RT 99
41 CirclePict,StimD,B,0,25   A 0  0    RT 134
42 SticksPict,StimB,B,25,0   A 0  0    RT 220
43 Green_Pict,StimA,A,25,0   A 1  1    RT 161
44 SticksPict,StimB,B,25,0   A 0  0    RT 359
45 Worms_Pict,StimC,A,0,25   A 1  1    RT 99
46 SticksPict,StimB,B,25,0   A 0  0    RT 180
47 Green_Pict,StimA,A,25,0   A 1  1    RT 100
48 CirclePict,StimD,B,0,25   A 0  0    RT 259
49 SticksPict,StimB,B,25,0   B 1  1    RT 274
50 Green_Pict,StimA,A,25,0   A 1  1    RT 156
51 CirclePict,StimD,B,0,25   A 0  0    RT 146
52 Worms_Pict,StimC,A,0,25   B 0  0    RT 75
53 CirclePict,StimD,B,0,25   A 0  0    RT 78
54 CirclePict,StimD,B,0,25   A 0  0    RT 58
55 Green_Pict,StimA,B,25,0   A 0  1    RT 87
56 SticksPict,StimB,B,25,0   A 0  0    RT 101
57 SticksPict,StimB,B,25,0   A 0  0    RT 144
58 Worms_Pict,StimC,A,0,25   B 0  0    RT 123
59 SticksPict,StimB,A,25,0   A 1  0    RT 80
60 Worms_Pict,StimC,B,0,25   A 0  1    RT 133
61 Worms_Pict,StimC,A,0,25   B 0  0    RT 206
62 Green_Pict,StimA,A,25,0   A 1  1    RT 144
63 SticksPict,StimB,A,25,0   A 1  0    RT 102
64 SticksPict,StimB,B,25,0   A 0  0    RT 343
65 Worms_Pict,StimC,A,0,25   A 1  1    RT 131
66 Green_Pict,StimA,A,25,0   A 1  1    RT 67
67 Worms_Pict,StimC,A,0,25   A 1  1    RT 292
68 Worms_Pict,StimC,A,0,25   A 1  1    RT 79
69 SticksPict,StimB,B,25,0   A 0  0    RT 179
70 CirclePict,StimD,A,0,25   A 1  0    RT 121
71 Green_Pict,StimA,A,25,0   A 1  1    RT 104
72 Green_Pict,StimA,A,25,0   A 1  1    RT 154
73 Worms_Pict,StimC,A,0,25   B 0  0    RT 64
74 CirclePict,StimD,B,0,25   A 0  0    RT 174
75 Green_Pict,StimA,B,25,0   A 0  1    RT 60
76 CirclePict,StimD,A,0,25   A 1  0    RT 60
77 CirclePict,StimD,B,0,25   A 0  0    RT 81
78 Green_Pict,StimA,A,25,0   A 1  1    RT 111
79 Worms_Pict,StimC,B,0,25   A 0  1    RT 68
80 CirclePict,StimD,B,0,25   B 1  1    RT 90
81 Green_Pict,StimA,A,25,0   A 1  1    RT 271
82 Green_Pict,StimA,A,25,0   A 1  1    RT 123
83 SticksPict,StimB,B,25,0   A 0  0    RT 200
84 Worms_Pict,StimC,A,0,25   A 1  1    RT 67
85 CirclePict,StimD,B,0,25   B 1  1    RT 145
86 Worms_Pict,StimC,A,0,25   A 1  1    RT 66
87 Worms_Pict,StimC,B,0,25   A 0  1    RT 75
88 Green_Pict,StimA,A,25,0   A 1  1    RT 180
89 SticksPict,StimB,B,25,0   B 1  1    RT 136
90 Worms_Pict,StimC,A,0,25   A 1  1    RT 185
91 CirclePict,StimD,B,0,25   A 0  0    RT 71
92 SticksPict,StimB,B,25,0   A 0  0    RT 69
93 CirclePict,StimD,B,0,25   A 0  0    RT 215
94 SticksPict,StimB,A,25,0   B 0  1    RT 55
95 CirclePict,StimD,B,0,25   A 0  0    RT 85
96 CirclePict,StimD,A,0,25   A 1  0    RT 120
97 CirclePict,StimD,B,0,25   A 0  0    RT 152
98 SticksPict,StimB,B,25,0   A 0  0    RT 113
99 Green_Pict,StimA,A,25,0   A 1  1    RT 92
100 CirclePict,StimD,B,0,25   B 1  1    RT 85
101 Green_Pict,StimA,A,25,0   A 1  1    RT 58
102 Worms_Pict,StimC,A,0,25   A 1  1    RT 78
103 Green_Pict,StimA,B,25,0   A 0  1    RT 102
104 Green_Pict,StimA,A,25,0   A 1  1    RT 71
105 Green_Pict,StimA,B,25,0   A 0  1    RT 145
106 SticksPict,StimB,B,25,0   B 1  1    RT 91
107 CirclePict,StimD,B,0,25   A 0  0    RT 164
108 Worms_Pict,StimC,A,0,25   A 1  1    RT 72
109 SticksPict,StimB,B,25,0   A 0  0    RT 115
110 SticksPict,StimB,B,25,0   B 1  1    RT 81
111 CirclePict,StimD,A,0,25   A 1  0    RT 67
112 Worms_Pict,StimC,A,0,25   A 1  1    RT 71
113 Green_Pict,StimA,A,25,0   A 1  1    RT 71
114 SticksPict,StimB,A,25,0   B 0  1    RT 85
115 Worms_Pict,StimC,A,0,25   A 1  1    RT 63
116 CirclePict,StimD,B,0,25   A 0  0    RT 68
117 SticksPict,StimB,B,25,0   A 0  0    RT 107
118 Worms_Pict,StimC,B,0,25   A 0  1    RT 82
119 Green_Pict,StimA,A,25,0   A 1  1    RT 79
120 Worms_Pict,StimC,A,0,25   A 1  1    RT 67
121 SticksPict,StimB,A,25,0   B 0  1    RT 75
122 Worms_Pict,StimC,A,0,25   A 1  1    RT 76
123 SticksPict,StimB,B,25,0   A 0  0    RT 63
124 Worms_Pict,StimC,B,0,25   B 1  0    RT 115
125 Green_Pict,StimA,A,25,0   A 1  1    RT 57
126 CirclePict,StimD,B,0,25   A 0  0    RT 126
127 CirclePict,StimD,B,0,25   A 0  0    RT 149
128 Green_Pict,StimA,A,25,0   A 1  1    RT 58
129 Green_Pict,StimA,A,25,0   A 1  1    RT 96
130 Green_Pict,StimA,A,25,0   A 1  1    RT 143
131 CirclePict,StimD,B,0,25   B 1  1    RT 74
132 Green_Pict,StimA,B,25,0   A 0  1    RT 100
133 Worms_Pict,StimC,A,0,25   A 1  1    RT 65
134 SticksPict,StimB,B,25,0   B 1  1    RT 81
135 Worms_Pict,StimC,A,0,25   A 1  1    RT 78
136 Worms_Pict,StimC,A,0,25   A 1  1    RT 116
137 Worms_Pict,StimC,A,0,25   B 0  0    RT 117
138 CirclePict,StimD,B,0,25   A 0  0    RT 81
139 Worms_Pict,StimC,B,0,25   A 0  1    RT 78
140 Worms_Pict,StimC,A,0,25   B 0  0    RT 58
141 Green_Pict,StimA,A,25,0   A 1  1    RT 52
142 Green_Pict,StimA,A,25,0   A 1  1    RT 126
143 SticksPict,StimB,B,25,0   A 0  0    RT 80
144 Worms_Pict,StimC,A,0,25   A 1  1    RT 89
145 SticksPict,StimB,B,25,0   B 1  1    RT 87
146 CirclePict,StimD,B,0,25   A 0  0    RT 68
147 Green_Pict,StimA,A,25,0   A 1  1    RT 53
148 Green_Pict,StimA,A,25,0   A 1  1    RT 339
149 SticksPict,StimB,B,25,0   B 1  1    RT 62
150 Worms_Pict,StimC,A,0,25   A 1  1    RT 217
151 SticksPict,StimB,B,25,0   B 1  1    RT 168
152 SticksPict,StimB,B,25,0   A 0  0    RT 198
153 SticksPict,StimB,A,25,0   A 1  0    RT 94
154 CirclePict,StimD,B,0,25   B 1  1    RT 94
155 Green_Pict,StimA,B,25,0   A 0  1    RT 71
156 CirclePict,StimD,B,0,25   A 0  0    RT 88
157 CirclePict,StimD,A,0,25   A 1  0    RT 73
158 CirclePict,StimD,A,0,25   B 0  1    RT 81
159 SticksPict,StimB,B,25,0   B 1  1    RT 118
160 CirclePict,StimD,B,0,25   A 0  0    RT 62


Total AB Correct = 45 (56.25% correct)
Total CD Correct = 36 (45% correct)
Total AB optimal = 55 (68.75% optimal)
Total CD optimal = 36 (45% optimal)
Total points = 525
```
