# Supplementary material for: Identifying the Basal Ganglia Network Model Markers for Medication-Induced Impulsivity in Parkinson's Disease Patients
Source: PLoS One. 2015 Jun 4;10(6):e0127542. doi: 10.1371/journal.pone.0127542 (PMC4456385; doi:10.1371/journal.pone.0127542)
Supplement: S4 File — (ZIP) [file pone.0127542.s004.zip › SMat/Quarters_HC.doc]

```
Quarters
Subject ID: HC
Stimuli:
A = Green_Pict B= CirclePict
C = Worms_Pict C= SticksPict
Stim A is class A 80% of trials; Stim B is class A 20% of trials
    win = 25 points, lose is 0
Stim C is class A 80% of trials; Stim D is class A 20% of trials
    win = 0, lose = 25 points


Key: A/B is subj response, 0/1 is correct?, 0/1 is optimal?
1 CirclePict,StimB,A,25,0   A 1  0    RT 400
2 SticksPict,StimD,A,0,25   A 1  0    RT 192
3 SticksPict,StimD,B,0,25   B 1  1    RT 103
4 CirclePict,StimB,A,25,0   A 1  0    RT 82
5 CirclePict,StimB,B,25,0   A 0  0    RT 104
6 SticksPict,StimD,B,0,25   B 1  1    RT 61
7 SticksPict,StimD,B,0,25   A 0  0    RT 51
8 Green_Pict,StimA,B,25,0   A 0  1    RT 61
9 Green_Pict,StimA,A,25,0   A 1  1    RT 41
10 Green_Pict,StimA,A,25,0   B 0  0    RT 165
11 SticksPict,StimD,B,0,25   A 0  0    RT 64
12 Green_Pict,StimA,A,25,0   A 1  1    RT 51
13 SticksPict,StimD,B,0,25   A 0  0    RT 57
14 Worms_Pict,StimC,A,0,25   A 1  1    RT 64
15 CirclePict,StimB,B,25,0   A 0  0    RT 47
16 Worms_Pict,StimC,A,0,25   A 1  1    RT 45
17 CirclePict,StimB,B,25,0   B 1  1    RT 61
18 CirclePict,StimB,B,25,0   B 1  1    RT 71
19 Worms_Pict,StimC,A,0,25   B 0  0    RT 67
20 CirclePict,StimB,B,25,0   A 0  0    RT 73
21 CirclePict,StimB,B,25,0   B 1  1    RT 41
22 Worms_Pict,StimC,A,0,25   A 1  1    RT 59
23 SticksPict,StimD,A,0,25   A 1  0    RT 213
24 SticksPict,StimD,B,0,25   A 0  0    RT 54
25 SticksPict,StimD,B,0,25   B 1  1    RT 32
26 Green_Pict,StimA,A,25,0   A 1  1    RT 43
27 Worms_Pict,StimC,A,0,25   A 1  1    RT 82
28 Worms_Pict,StimC,B,0,25   B 1  0    RT 38
29 Green_Pict,StimA,B,25,0   A 0  1    RT 39
30 Green_Pict,StimA,A,25,0   A 1  1    RT 28
31 Worms_Pict,StimC,B,0,25   A 0  1    RT 102
32 SticksPict,StimD,B,0,25   B 1  1    RT 70
33 Green_Pict,StimA,A,25,0   A 1  1    RT 47
34 Worms_Pict,StimC,A,0,25   A 1  1    RT 58
35 CirclePict,StimB,B,25,0   A 0  0    RT 29
36 Green_Pict,StimA,A,25,0   A 1  1    RT 34
37 Worms_Pict,StimC,A,0,25   A 1  1    RT 37
38 Worms_Pict,StimC,A,0,25   A 1  1    RT 34
39 Green_Pict,StimA,A,25,0   A 1  1    RT 34
40 CirclePict,StimB,B,25,0   A 0  0    RT 37
41 CirclePict,StimB,B,25,0   A 0  0    RT 46
42 SticksPict,StimD,B,0,25   B 1  1    RT 79
43 Worms_Pict,StimC,A,0,25   B 0  0    RT 76
44 SticksPict,StimD,B,0,25   B 1  1    RT 75
45 Green_Pict,StimA,A,25,0   A 1  1    RT 28
46 CirclePict,StimB,A,25,0   A 1  0    RT 30
47 Green_Pict,StimA,A,25,0   A 1  1    RT 49
48 Worms_Pict,StimC,A,0,25   A 1  1    RT 39
49 Worms_Pict,StimC,A,0,25   B 0  0    RT 51
50 SticksPict,StimD,B,0,25   B 1  1    RT 120
51 SticksPict,StimD,B,0,25   B 1  1    RT 74
52 Green_Pict,StimA,A,25,0   A 1  1    RT 34
53 Green_Pict,StimA,A,25,0   A 1  1    RT 30
54 CirclePict,StimB,B,25,0   A 0  0    RT 34
55 CirclePict,StimB,B,25,0   A 0  0    RT 22
56 SticksPict,StimD,B,0,25   A 0  0    RT 79
57 Green_Pict,StimA,A,25,0   A 1  1    RT 30
58 CirclePict,StimB,B,25,0   A 0  0    RT 33
59 Worms_Pict,StimC,A,0,25   A 1  1    RT 38
60 SticksPict,StimD,B,0,25   B 1  1    RT 69
61 Green_Pict,StimA,B,25,0   A 0  1    RT 32
62 CirclePict,StimB,B,25,0   A 0  0    RT 39
63 Green_Pict,StimA,A,25,0   A 1  1    RT 38
64 Worms_Pict,StimC,A,0,25   A 1  1    RT 74
65 SticksPict,StimD,B,0,25   B 1  1    RT 68
66 Worms_Pict,StimC,A,0,25   B 0  0    RT 146
67 CirclePict,StimB,B,25,0   A 0  0    RT 38
68 CirclePict,StimB,B,25,0   B 1  1    RT 31
69 CirclePict,StimB,A,25,0   B 0  1    RT 66
70 CirclePict,StimB,B,25,0   B 1  1    RT 40
71 Green_Pict,StimA,A,25,0   A 1  1    RT 42
72 Green_Pict,StimA,A,25,0   A 1  1    RT 43
73 Green_Pict,StimA,B,25,0   A 0  1    RT 46
74 Worms_Pict,StimC,A,0,25   A 1  1    RT 93
75 SticksPict,StimD,A,0,25   B 0  1    RT 44
76 SticksPict,StimD,A,0,25   B 0  1    RT 55
77 Worms_Pict,StimC,B,0,25   A 0  1    RT 41
78 SticksPict,StimD,B,0,25   B 1  1    RT 45
79 Worms_Pict,StimC,A,0,25   A 1  1    RT 54
80 Worms_Pict,StimC,B,0,25   A 0  1    RT 36
81 SticksPict,StimD,B,0,25   B 1  1    RT 33
82 CirclePict,StimB,B,25,0   A 0  0    RT 43
83 SticksPict,StimD,B,0,25   B 1  1    RT 43
84 CirclePict,StimB,B,25,0   B 1  1    RT 23
85 Worms_Pict,StimC,A,0,25   A 1  1    RT 107
86 Green_Pict,StimA,A,25,0   A 1  1    RT 32
87 Worms_Pict,StimC,B,0,25   A 0  1    RT 72
88 CirclePict,StimB,B,25,0   B 1  1    RT 52
89 CirclePict,StimB,B,25,0   B 1  1    RT 58
90 CirclePict,StimB,A,25,0   A 1  0    RT 76
91 SticksPict,StimD,A,0,25   A 1  0    RT 127
92 SticksPict,StimD,B,0,25   B 1  1    RT 48
93 Worms_Pict,StimC,B,0,25   A 0  1    RT 133
94 SticksPict,StimD,B,0,25   B 1  1    RT 53
95 Worms_Pict,StimC,A,0,25   B 0  0    RT 51
96 Worms_Pict,StimC,A,0,25   A 1  1    RT 43
97 SticksPict,StimD,B,0,25   B 1  1    RT 46
98 CirclePict,StimB,B,25,0   B 1  1    RT 47
99 SticksPict,StimD,A,0,25   B 0  1    RT 43
100 Worms_Pict,StimC,A,0,25   A 1  1    RT 33
101 Green_Pict,StimA,A,25,0   A 1  1    RT 35
102 SticksPict,StimD,B,0,25   A 0  0    RT 52
103 Green_Pict,StimA,A,25,0   A 1  1    RT 29
104 Green_Pict,StimA,A,25,0   A 1  1    RT 26
105 Green_Pict,StimA,B,25,0   B 1  0    RT 27
106 Worms_Pict,StimC,A,0,25   B 0  0    RT 59
107 CirclePict,StimB,B,25,0   B 1  1    RT 50
108 Worms_Pict,StimC,A,0,25   A 1  1    RT 74
109 Green_Pict,StimA,A,25,0   A 1  1    RT 49
110 SticksPict,StimD,B,0,25   B 1  1    RT 88
111 Green_Pict,StimA,A,25,0   A 1  1    RT 41
112 CirclePict,StimB,B,25,0   B 1  1    RT 50
113 CirclePict,StimB,B,25,0   B 1  1    RT 47
114 Worms_Pict,StimC,A,0,25   A 1  1    RT 146
115 CirclePict,StimB,A,25,0   A 1  0    RT 50
116 SticksPict,StimD,B,0,25   A 0  0    RT 74
117 Green_Pict,StimA,B,25,0   A 0  1    RT 40
118 Green_Pict,StimA,A,25,0   A 1  1    RT 55
119 Worms_Pict,StimC,A,0,25   B 0  0    RT 188
120 Green_Pict,StimA,A,25,0   A 1  1    RT 29
121 Worms_Pict,StimC,B,0,25   A 0  1    RT 42
122 CirclePict,StimB,B,25,0   A 0  0    RT 37
123 SticksPict,StimD,B,0,25   B 1  1    RT 70
124 SticksPict,StimD,B,0,25   B 1  1    RT 59
125 Green_Pict,StimA,A,25,0   A 1  1    RT 24
126 CirclePict,StimB,A,25,0   A 1  0    RT 34
127 SticksPict,StimD,B,0,25   B 1  1    RT 46
128 SticksPict,StimD,B,0,25   B 1  1    RT 37
129 Worms_Pict,StimC,A,0,25   B 0  0    RT 54
130 SticksPict,StimD,B,0,25   A 0  0    RT 57
131 CirclePict,StimB,B,25,0   A 0  0    RT 55
132 Green_Pict,StimA,A,25,0   A 1  1    RT 35
133 CirclePict,StimB,B,25,0   A 0  0    RT 46
134 Green_Pict,StimA,A,25,0   A 1  1    RT 41
135 Green_Pict,StimA,A,25,0   A 1  1    RT 29
136 Green_Pict,StimA,A,25,0   B 0  0    RT 27
137 SticksPict,StimD,A,0,25   B 0  1    RT 72
138 Worms_Pict,StimC,A,0,25   B 0  0    RT 55
139 Worms_Pict,StimC,A,0,25   A 1  1    RT 39
140 Worms_Pict,StimC,A,0,25   A 1  1    RT 27
141 SticksPict,StimD,B,0,25   A 0  0    RT 48
142 CirclePict,StimB,B,25,0   B 1  1    RT 64
143 Worms_Pict,StimC,A,0,25   B 0  0    RT 40
144 CirclePict,StimB,B,25,0   A 0  0    RT 28
145 Worms_Pict,StimC,A,0,25   B 0  0    RT 80
146 CirclePict,StimB,B,25,0   B 1  1    RT 53
147 Worms_Pict,StimC,A,0,25   A 1  1    RT 45
148 CirclePict,StimB,B,25,0   A 0  0    RT 45
149 Green_Pict,StimA,B,25,0   A 0  1    RT 33
150 Green_Pict,StimA,A,25,0   A 1  1    RT 28
151 Worms_Pict,StimC,B,0,25   A 0  1    RT 109
152 Green_Pict,StimA,A,25,0   A 1  1    RT 40
153 Green_Pict,StimA,B,25,0   A 0  1    RT 111
154 CirclePict,StimB,A,25,0   A 1  0    RT 41
155 Worms_Pict,StimC,A,0,25   A 1  1    RT 62
156 SticksPict,StimD,B,0,25   B 1  1    RT 61
157 Green_Pict,StimA,A,25,0   A 1  1    RT 44
158 CirclePict,StimB,B,25,0   A 0  0    RT 40
159 SticksPict,StimD,B,0,25   B 1  1    RT 49
160 SticksPict,StimD,A,0,25   B 0  1    RT 54


Total AB Correct = 52 (65% correct)
Total CD Correct = 48 (60% correct)
Total AB optimal = 52 (65% optimal)
Total CD optimal = 56 (70% optimal)
Total points = 1000
```
