# Supplementary material for: Identifying the Basal Ganglia Network Model Markers for Medication-Induced Impulsivity in Parkinson's Disease Patients
Source: PLoS One. 2015 Jun 4;10(6):e0127542. doi: 10.1371/journal.pone.0127542 (PMC4456385; doi:10.1371/journal.pone.0127542)
Supplement: S4 File — (ZIP) [file pone.0127542.s004.zip › SMat/Quarters_HC12.doc]

```
QuartersCF4
Subject ID: HC 12
 Stimuli:
A = CirclePict B= Green_Pict
C = SticksPict C= Worms_Pict
Stim A is class A 80% of trials; Stim B is class A 20% of trials
    win = 25 points, lose is 0
Stim C is class A 80% of trials; Stim D is class A 20% of trials
    win = 0, lose = 25 points


Key: A/B is subj response, 0/1 is correct?, 0/1 is optimal?
1 SticksPict,StimC,B,0,25   A 0  1    RT 152
2 SticksPict,StimC,A,0,25   B 0  0    RT 98
3 Green_Pict,StimB,B,25,0   B 1  1    RT 247
4 Worms_Pict,StimD,B,0,25   A 0  0    RT 231
5 Green_Pict,StimB,B,25,0   B 1  1    RT 81
6 CirclePict,StimA,B,25,0   A 0  1    RT 155
7 Worms_Pict,StimD,B,0,25   B 1  1    RT 118
8 SticksPict,StimC,A,0,25   B 0  0    RT 63
9 Green_Pict,StimB,B,25,0   B 1  1    RT 87
10 Worms_Pict,StimD,B,0,25   A 0  0    RT 99
11 CirclePict,StimA,A,25,0   A 1  1    RT 64
12 CirclePict,StimA,B,25,0   A 0  1    RT 55
13 Worms_Pict,StimD,A,0,25   B 0  1    RT 114
14 SticksPict,StimC,A,0,25   A 1  1    RT 66
15 Worms_Pict,StimD,A,0,25   B 0  1    RT 133
16 Worms_Pict,StimD,B,0,25   A 0  0    RT 75
17 Green_Pict,StimB,B,25,0   B 1  1    RT 274
18 Worms_Pict,StimD,B,0,25   B 1  1    RT 92
19 Green_Pict,StimB,B,25,0   B 1  1    RT 39
20 CirclePict,StimA,A,25,0   A 1  1    RT 89
21 Green_Pict,StimB,B,25,0   B 1  1    RT 34
22 Green_Pict,StimB,A,25,0   B 0  1    RT 37
23 Worms_Pict,StimD,B,0,25   B 1  1    RT 69
24 SticksPict,StimC,A,0,25   B 0  0    RT 76
25 SticksPict,StimC,A,0,25   A 1  1    RT 66
26 CirclePict,StimA,A,25,0   A 1  1    RT 144
27 CirclePict,StimA,A,25,0   A 1  1    RT 89
28 Green_Pict,StimB,A,25,0   B 0  1    RT 32
29 Worms_Pict,StimD,B,0,25   A 0  0    RT 109
30 SticksPict,StimC,A,0,25   A 1  1    RT 88
31 SticksPict,StimC,B,0,25   A 0  1    RT 47
32 SticksPict,StimC,A,0,25   A 1  1    RT 45
33 CirclePict,StimA,A,25,0   A 1  1    RT 41
34 SticksPict,StimC,A,0,25   B 0  0    RT 45
35 Green_Pict,StimB,B,25,0   B 1  1    RT 124
36 CirclePict,StimA,A,25,0   B 0  0    RT 31
37 Worms_Pict,StimD,B,0,25   A 0  0    RT 102
38 CirclePict,StimA,A,25,0   A 1  1    RT 46
39 CirclePict,StimA,A,25,0   B 0  0    RT 99
40 Green_Pict,StimB,B,25,0   B 1  1    RT 54
41 CirclePict,StimA,A,25,0   A 1  1    RT 70
42 SticksPict,StimC,A,0,25   B 0  0    RT 40
43 CirclePict,StimA,A,25,0   B 0  0    RT 17
44 CirclePict,StimA,A,25,0   B 0  0    RT 42
45 Green_Pict,StimB,A,25,0   B 0  1    RT 31
46 Worms_Pict,StimD,B,0,25   B 1  1    RT 19
47 SticksPict,StimC,A,0,25   B 0  0    RT 31
48 SticksPict,StimC,A,0,25   B 0  0    RT 12
49 Green_Pict,StimB,B,25,0   B 1  1    RT 15
50 SticksPict,StimC,B,0,25   B 1  0    RT 12
51 CirclePict,StimA,A,25,0   B 0  0    RT 21
52 Green_Pict,StimB,B,25,0   B 1  1    RT 13
53 Green_Pict,StimB,B,25,0   A 0  0    RT 34
54 Worms_Pict,StimD,B,0,25   A 0  0    RT 15
55 CirclePict,StimA,B,25,0   A 0  1    RT 14
56 SticksPict,StimC,A,0,25   B 0  0    RT 41
57 Worms_Pict,StimD,B,0,25   A 0  0    RT 15
58 Green_Pict,StimB,A,25,0   A 1  0    RT 10
59 Green_Pict,StimB,B,25,0   A 0  0    RT 14
60 Green_Pict,StimB,B,25,0   A 0  0    RT 13
61 Worms_Pict,StimD,B,0,25   A 0  0    RT 11
62 Worms_Pict,StimD,B,0,25   B 1  1    RT 13
63 CirclePict,StimA,A,25,0   B 0  0    RT 51
64 SticksPict,StimC,A,0,25   B 0  0    RT 16
65 Worms_Pict,StimD,B,0,25   B 1  1    RT 13
66 Green_Pict,StimB,B,25,0   B 1  1    RT 12
67 SticksPict,StimC,B,0,25   B 1  0    RT 14
68 SticksPict,StimC,A,0,25   B 0  0    RT 11
69 CirclePict,StimA,A,25,0   B 0  0    RT 13
70 SticksPict,StimC,A,0,25   B 0  0    RT 13
71 Worms_Pict,StimD,B,0,25   B 1  1    RT 15
72 Green_Pict,StimB,B,25,0   B 1  1    RT 17
73 CirclePict,StimA,B,25,0   A 0  1    RT 35
74 Worms_Pict,StimD,B,0,25   A 0  0    RT 174
75 Worms_Pict,StimD,A,0,25   B 0  1    RT 225
76 CirclePict,StimA,A,25,0   A 1  1    RT 39
77 Green_Pict,StimB,B,25,0   B 1  1    RT 21
78 CirclePict,StimA,A,25,0   A 1  1    RT 43
79 Worms_Pict,StimD,A,0,25   B 0  1    RT 53
80 SticksPict,StimC,A,0,25   B 0  0    RT 54
81 Green_Pict,StimB,B,25,0   B 1  1    RT 34
82 Green_Pict,StimB,B,25,0   B 1  1    RT 40
83 Worms_Pict,StimD,B,0,25   A 0  0    RT 68
84 Green_Pict,StimB,A,25,0   B 0  1    RT 46
85 SticksPict,StimC,A,0,25   B 0  0    RT 55
86 CirclePict,StimA,A,25,0   A 1  1    RT 39
87 SticksPict,StimC,A,0,25   A 1  1    RT 67
88 Green_Pict,StimB,B,25,0   B 1  1    RT 37
89 Worms_Pict,StimD,B,0,25   A 0  0    RT 83
90 CirclePict,StimA,A,25,0   A 1  1    RT 34
91 Green_Pict,StimB,A,25,0   B 0  1    RT 35
92 Worms_Pict,StimD,A,0,25   B 0  1    RT 45
93 Worms_Pict,StimD,B,0,25   A 0  0    RT 165
94 SticksPict,StimC,A,0,25   B 0  0    RT 49
95 Worms_Pict,StimD,B,0,25   A 0  0    RT 67
96 CirclePict,StimA,A,25,0   A 1  1    RT 34
97 Green_Pict,StimB,B,25,0   B 1  1    RT 27
98 Green_Pict,StimB,B,25,0   B 1  1    RT 38
99 SticksPict,StimC,A,0,25   B 0  0    RT 81
100 CirclePict,StimA,A,25,0   A 1  1    RT 29
101 SticksPict,StimC,A,0,25   A 1  1    RT 43
102 CirclePict,StimA,B,25,0   A 0  1    RT 54
103 SticksPict,StimC,A,0,25   A 1  1    RT 65
104 CirclePict,StimA,A,25,0   A 1  1    RT 49
105 CirclePict,StimA,A,25,0   A 1  1    RT 39
106 Worms_Pict,StimD,B,0,25   B 1  1    RT 65
107 Green_Pict,StimB,B,25,0   B 1  1    RT 31
108 Worms_Pict,StimD,B,0,25   B 1  1    RT 67
109 Worms_Pict,StimD,B,0,25   B 1  1    RT 35
110 SticksPict,StimC,A,0,25   A 1  1    RT 78
111 Green_Pict,StimB,B,25,0   B 1  1    RT 39
112 CirclePict,StimA,A,25,0   A 1  1    RT 38
113 Green_Pict,StimB,B,25,0   B 1  1    RT 35
114 SticksPict,StimC,A,0,25   A 1  1    RT 40
115 CirclePict,StimA,A,25,0   A 1  1    RT 42
116 Worms_Pict,StimD,A,0,25   B 0  1    RT 59
117 SticksPict,StimC,B,0,25   A 0  1    RT 53
118 SticksPict,StimC,B,0,25   A 0  1    RT 68
119 Worms_Pict,StimD,B,0,25   A 0  0    RT 63
120 CirclePict,StimA,B,25,0   A 0  1    RT 40
121 Green_Pict,StimB,B,25,0   B 1  1    RT 34
122 Green_Pict,StimB,B,25,0   B 1  1    RT 36
123 SticksPict,StimC,A,0,25   A 1  1    RT 48
124 Worms_Pict,StimD,B,0,25   B 1  1    RT 75
125 SticksPict,StimC,A,0,25   A 1  1    RT 96
126 Green_Pict,StimB,B,25,0   B 1  1    RT 39
127 CirclePict,StimA,A,25,0   A 1  1    RT 40
128 SticksPict,StimC,A,0,25   A 1  1    RT 50
129 CirclePict,StimA,A,25,0   A 1  1    RT 38
130 SticksPict,StimC,A,0,25   A 1  1    RT 36
131 CirclePict,StimA,A,25,0   A 1  1    RT 36
132 Green_Pict,StimB,B,25,0   B 1  1    RT 37
133 Worms_Pict,StimD,A,0,25   B 0  1    RT 36
134 SticksPict,StimC,A,0,25   A 1  1    RT 45
135 CirclePict,StimA,A,25,0   A 1  1    RT 39
136 CirclePict,StimA,A,25,0   A 1  1    RT 31
137 Worms_Pict,StimD,B,0,25   B 1  1    RT 47
138 SticksPict,StimC,A,0,25   A 1  1    RT 40
139 Worms_Pict,StimD,B,0,25   B 1  1    RT 51
140 SticksPict,StimC,B,0,25   A 0  1    RT 34
141 Worms_Pict,StimD,B,0,25   B 1  1    RT 39
142 Worms_Pict,StimD,B,0,25   A 0  0    RT 44
143 Worms_Pict,StimD,A,0,25   B 0  1    RT 43
144 CirclePict,StimA,A,25,0   A 1  1    RT 35
145 Green_Pict,StimB,B,25,0   B 1  1    RT 33
146 SticksPict,StimC,B,0,25   A 0  1    RT 37
147 CirclePict,StimA,A,25,0   A 1  1    RT 36
148 Green_Pict,StimB,A,25,0   B 0  1    RT 29
149 CirclePict,StimA,B,25,0   A 0  1    RT 62
150 SticksPict,StimC,A,0,25   A 1  1    RT 38
151 Worms_Pict,StimD,B,0,25   B 1  1    RT 37
152 Worms_Pict,StimD,B,0,25   B 1  1    RT 35
153 Worms_Pict,StimD,B,0,25   B 1  1    RT 40
154 Green_Pict,StimB,B,25,0   B 1  1    RT 35
155 Green_Pict,StimB,A,25,0   B 0  1    RT 28
156 Green_Pict,StimB,B,25,0   B 1  1    RT 35
157 CirclePict,StimA,B,25,0   A 0  1    RT 40
158 Green_Pict,StimB,B,25,0   A 0  0    RT 39
159 CirclePict,StimA,A,25,0   A 1  1    RT 29
160 SticksPict,StimC,A,0,25   B 0  0    RT 3


Total AB Correct = 54 (67.5% correct)
Total CD Correct = 35 (43.75% correct)
Total AB optimal = 68 (85% optimal)
Total CD optimal = 47 (58.75% optimal)
Total points = 725
```
