# Supplementary material for: Identifying the Basal Ganglia Network Model Markers for Medication-Induced Impulsivity in Parkinson's Disease Patients
Source: PLoS One. 2015 Jun 4;10(6):e0127542. doi: 10.1371/journal.pone.0127542 (PMC4456385; doi:10.1371/journal.pone.0127542)
Supplement: S4 File — (ZIP) [file pone.0127542.s004.zip › SMat/Quarters_HC13.doc]

```
QuartersCF4
Subject ID: HC 13
 Stimuli:
A = SticksPict B= Green_Pict
C = Worms_Pict C= CirclePict
Stim A is class A 80% of trials; Stim B is class A 20% of trials
    win = 25 points, lose is 0
Stim C is class A 80% of trials; Stim D is class A 20% of trials
    win = 0, lose = 25 points


Key: A/B is subj response, 0/1 is correct?, 0/1 is optimal?
1 Worms_Pict,StimC,A,0,25   A 1  1    RT 417
2 CirclePict,StimD,B,0,25   A 0  0    RT 261
3 Worms_Pict,StimC,A,0,25   B 0  0    RT 77
4 Worms_Pict,StimC,A,0,25   B 0  0    RT 158
5 CirclePict,StimD,B,0,25   B 1  1    RT 106
6 CirclePict,StimD,B,0,25   B 1  1    RT 83
7 CirclePict,StimD,B,0,25   B 1  1    RT 99
8 Worms_Pict,StimC,A,0,25   A 1  1    RT 157
9 Worms_Pict,StimC,B,0,25   A 0  1    RT 101
10 Worms_Pict,StimC,A,0,25   B 0  0    RT 740
11 CirclePict,StimD,B,0,25   B 1  1    RT 81
12 Worms_Pict,StimC,A,0,25   B 0  0    RT 144
13 CirclePict,StimD,A,0,25   B 0  1    RT 180
14 Worms_Pict,StimC,B,0,25   A 0  1    RT 74
15 CirclePict,StimD,A,0,25   B 0  1    RT 117
16 Worms_Pict,StimC,A,0,25   B 0  0    RT 36
17 Worms_Pict,StimC,A,0,25   A 1  1    RT 73
18 CirclePict,StimD,B,0,25   A 0  0    RT 159
19 CirclePict,StimD,B,0,25   B 1  1    RT 39
20 CirclePict,StimD,B,0,25   B 1  1    RT 37
21 Worms_Pict,StimC,B,0,25   B 1  0    RT 152
22 Worms_Pict,StimC,A,0,25   B 0  0    RT 119
23 CirclePict,StimD,A,0,25   A 1  0    RT 57
24 CirclePict,StimD,A,0,25   A 1  0    RT 20
25 Worms_Pict,StimC,A,0,25   A 1  1    RT 21
26 Worms_Pict,StimC,A,0,25   B 0  0    RT 40
27 CirclePict,StimD,B,0,25   A 0  0    RT 15
28 Worms_Pict,StimC,A,0,25   B 0  0    RT 19
29 CirclePict,StimD,B,0,25   B 1  1    RT 22
30 Worms_Pict,StimC,A,0,25   B 0  0    RT 22
31 Worms_Pict,StimC,A,0,25   A 1  1    RT 3
32 Worms_Pict,StimC,A,0,25   B 0  0    RT 16
33 CirclePict,StimD,B,0,25   A 0  0    RT 18
34 CirclePict,StimD,B,0,25   B 1  1    RT 33
35 CirclePict,StimD,B,0,25   B 1  1    RT 16
36 CirclePict,StimD,B,0,25   B 1  1    RT 28
37 Worms_Pict,StimC,B,0,25   B 1  0    RT 283
38 Worms_Pict,StimC,A,0,25   B 0  0    RT 37
39 CirclePict,StimD,B,0,25   A 0  0    RT 118
40 CirclePict,StimD,B,0,25   B 1  1    RT 262
41 Worms_Pict,StimC,A,0,25   B 0  0    RT 19
42 Worms_Pict,StimC,B,0,25   A 0  1    RT 23
43 CirclePict,StimD,B,0,25   B 1  1    RT 0
44 CirclePict,StimD,B,0,25   B 1  1    RT 31
45 Worms_Pict,StimC,A,0,25   B 0  0    RT 13
46 CirclePict,StimD,B,0,25   A 0  0    RT 10
47 CirclePict,StimD,B,0,25   B 1  1    RT 10
48 CirclePict,StimD,B,0,25   B 1  1    RT 17
49 Worms_Pict,StimC,A,0,25   B 0  0    RT 21
50 Worms_Pict,StimC,A,0,25   B 0  0    RT 37
51 Worms_Pict,StimC,B,0,25   B 1  0    RT 12
52 Worms_Pict,StimC,A,0,25   A 1  1    RT 34
53 CirclePict,StimD,A,0,25   A 1  0    RT 11
54 CirclePict,StimD,B,0,25   A 0  0    RT 16
55 Worms_Pict,StimC,A,0,25   B 0  0    RT 9
56 Worms_Pict,StimC,A,0,25   A 1  1    RT 11
57 CirclePict,StimD,B,0,25   A 0  0    RT 22
58 CirclePict,StimD,A,0,25   A 1  0    RT 15
59 CirclePict,StimD,B,0,25   B 1  1    RT 10
60 Worms_Pict,StimC,A,0,25   A 1  1    RT 21
61 CirclePict,StimD,B,0,25   B 1  1    RT 11
62 Worms_Pict,StimC,A,0,25   B 0  0    RT 25
63 CirclePict,StimD,B,0,25   A 0  0    RT 13
64 Worms_Pict,StimC,A,0,25   B 0  0    RT 7
65 Worms_Pict,StimC,A,0,25   A 1  1    RT 0
66 Worms_Pict,StimC,A,0,25   A 1  1    RT 18
67 Worms_Pict,StimC,B,0,25   A 0  1    RT 11
68 CirclePict,StimD,A,0,25   B 0  1    RT 14
69 CirclePict,StimD,B,0,25   A 0  0    RT 13
70 CirclePict,StimD,B,0,25   B 1  1    RT 0
71 CirclePict,StimD,B,0,25   A 0  0    RT 18
72 CirclePict,StimD,B,0,25   B 1  1    RT 0
73 Worms_Pict,StimC,A,0,25   A 1  1    RT 0
74 Worms_Pict,StimC,A,0,25   B 0  0    RT 3
75 CirclePict,StimD,A,0,25   B 0  1    RT 9
76 CirclePict,StimD,B,0,25   B 1  1    RT 5
77 Worms_Pict,StimC,A,0,25   B 0  0    RT 1
78 Worms_Pict,StimC,A,0,25   B 0  0    RT 0
79 Worms_Pict,StimC,B,0,25   B 1  0    RT 0
80 CirclePict,StimD,B,0,25   A 0  0    RT 0
1 SticksPict,StimA,A,25,0   B 0  0    RT 58
2 Green_Pict,StimB,B,25,0   B 1  1    RT 36
3 SticksPict,StimA,A,25,0   B 0  0    RT 22
4 SticksPict,StimA,B,25,0   A 0  1    RT 13
5 Green_Pict,StimB,B,25,0   B 1  1    RT 13
6 Green_Pict,StimB,B,25,0   A 0  0    RT 48
7 Green_Pict,StimB,B,25,0   A 0  0    RT 0
8 SticksPict,StimA,A,25,0   A 1  1    RT 13
9 Green_Pict,StimB,B,25,0   B 1  1    RT 9
10 Green_Pict,StimB,B,25,0   B 1  1    RT 10
11 SticksPict,StimA,A,25,0   A 1  1    RT 11
12 SticksPict,StimA,A,25,0   A 1  1    RT 11
13 SticksPict,StimA,A,25,0   B 0  0    RT 12
14 Green_Pict,StimB,A,25,0   A 1  0    RT 13
15 Green_Pict,StimB,B,25,0   A 0  0    RT 10
16 SticksPict,StimA,B,25,0   B 1  0    RT 15
17 Green_Pict,StimB,A,25,0   B 0  1    RT 14
18 Green_Pict,StimB,B,25,0   A 0  0    RT 0
19 SticksPict,StimA,A,25,0   B 0  0    RT 3
20 SticksPict,StimA,A,25,0   B 0  0    RT 4
21 SticksPict,StimA,A,25,0   B 0  0    RT 0
22 SticksPict,StimA,A,25,0   B 0  0    RT 22
23 Green_Pict,StimB,B,25,0   B 1  1    RT 0
24 SticksPict,StimA,A,25,0   B 0  0    RT 1
25 Green_Pict,StimB,B,25,0   B 1  1    RT 0
26 Green_Pict,StimB,B,25,0   B 1  1    RT 1
27 Green_Pict,StimB,B,25,0   B 1  1    RT 1
28 SticksPict,StimA,B,25,0   B 1  0    RT 1
29 SticksPict,StimA,B,25,0   A 0  1    RT 0
30 Green_Pict,StimB,A,25,0   B 0  1    RT 24
31 SticksPict,StimA,A,25,0   B 0  0    RT 12
32 Green_Pict,StimB,A,25,0   A 1  0    RT 17
33 Green_Pict,StimB,B,25,0   A 0  0    RT 1
34 Green_Pict,StimB,B,25,0   B 1  1    RT 13
35 SticksPict,StimA,A,25,0   B 0  0    RT 13
36 Green_Pict,StimB,B,25,0   A 0  0    RT 10
37 SticksPict,StimA,A,25,0   B 0  0    RT 11
38 SticksPict,StimA,A,25,0   B 0  0    RT 3
39 SticksPict,StimA,A,25,0   A 1  1    RT 23
40 Green_Pict,StimB,B,25,0   B 1  1    RT 12
41 Green_Pict,StimB,B,25,0   B 1  1    RT 11
42 Green_Pict,StimB,B,25,0   B 1  1    RT 17
43 SticksPict,StimA,A,25,0   B 0  0    RT 10
44 Green_Pict,StimB,B,25,0   A 0  0    RT 12
45 Green_Pict,StimB,B,25,0   B 1  1    RT 19
46 Green_Pict,StimB,A,25,0   B 0  1    RT 17
47 Green_Pict,StimB,B,25,0   A 0  0    RT 14
48 Green_Pict,StimB,B,25,0   B 1  1    RT 0
49 Green_Pict,StimB,B,25,0   B 1  1    RT 10
50 Green_Pict,StimB,A,25,0   B 0  1    RT 5
51 SticksPict,StimA,B,25,0   A 0  1    RT 10
52 SticksPict,StimA,A,25,0   B 0  0    RT 17
53 SticksPict,StimA,A,25,0   A 1  1    RT 4
54 SticksPict,StimA,A,25,0   B 0  0    RT 17
55 SticksPict,StimA,A,25,0   A 1  1    RT 10
56 SticksPict,StimA,A,25,0   A 1  1    RT 8
57 SticksPict,StimA,B,25,0   A 0  1    RT 11
58 SticksPict,StimA,A,25,0   B 0  0    RT 9
59 SticksPict,StimA,A,25,0   A 1  1    RT 6
60 Green_Pict,StimB,B,25,0   A 0  0    RT 9
61 SticksPict,StimA,B,25,0   A 0  1    RT 12
62 SticksPict,StimA,A,25,0   B 0  0    RT 0
63 Green_Pict,StimB,B,25,0   A 0  0    RT 12
64 SticksPict,StimA,A,25,0   B 0  0    RT 0
65 SticksPict,StimA,A,25,0   B 0  0    RT 0
66 Green_Pict,StimB,B,25,0   B 1  1    RT 0
67 SticksPict,StimA,B,25,0   B 1  0    RT 0
68 Green_Pict,StimB,B,25,0   B 1  1    RT 0
69 SticksPict,StimA,A,25,0   B 0  0    RT 0
70 Green_Pict,StimB,B,25,0   B 1  1    RT 61
71 SticksPict,StimA,A,25,0   B 0  0    RT 12
72 Green_Pict,StimB,B,25,0   A 0  0    RT 13
73 SticksPict,StimA,A,25,0   B 0  0    RT 6
74 Green_Pict,StimB,A,25,0   B 0  1    RT 0
75 SticksPict,StimA,A,25,0   B 0  0    RT 10
76 Green_Pict,StimB,B,25,0   B 1  1    RT 13
77 Green_Pict,StimB,A,25,0   B 0  1    RT 10
78 Green_Pict,StimB,B,25,0   A 0  0    RT 3
79 Green_Pict,StimB,B,25,0   B 1  1    RT 32
80 SticksPict,StimA,A,25,0   B 0  0    RT 1


Total AB Correct = 33 (41.25%)
Total CD Correct = 39 (48.75%)
Total AB Optimal = 39 (48.75%)
Total CD Optimal = 39 (48.75%)
Total points = 2200
```
