# Supplementary material for: Identifying the Basal Ganglia Network Model Markers for Medication-Induced Impulsivity in Parkinson's Disease Patients
Source: PLoS One. 2015 Jun 4;10(6):e0127542. doi: 10.1371/journal.pone.0127542 (PMC4456385; doi:10.1371/journal.pone.0127542)
Supplement: S4 File — (ZIP) [file pone.0127542.s004.zip › SMat/Quarters_HC14.doc]

```
QuartersCF4
Subject ID: HC 14
 Stimuli:
A = CirclePict B= SticksPict
C = Green_Pict C= Worms_Pict
Stim A is class A 80% of trials; Stim B is class A 20% of trials
    win = 25 points, lose is 0
Stim C is class A 80% of trials; Stim D is class A 20% of trials
    win = 0, lose = 25 points


Key: A/B is subj response, 0/1 is correct?, 0/1 is optimal?
1 Worms_Pict,StimD,B,0,25   A 0  0    RT 457
2 Worms_Pict,StimD,A,0,25   B 0  1    RT 254
3 Worms_Pict,StimD,B,0,25   A 0  0    RT 352
4 Green_Pict,StimC,A,0,25   B 0  0    RT 449
5 CirclePict,StimA,A,25,0   B 0  0    RT 381
6 Worms_Pict,StimD,B,0,25   A 0  0    RT 275
7 Green_Pict,StimC,A,0,25   A 1  1    RT 221
8 Green_Pict,StimC,A,0,25   A 1  1    RT 72
9 SticksPict,StimB,B,25,0   A 0  0    RT 241
10 Worms_Pict,StimD,B,0,25   A 0  0    RT 163
11 Worms_Pict,StimD,B,0,25   B 1  1    RT 74
12 SticksPict,StimB,B,25,0   A 0  0    RT 73
13 Worms_Pict,StimD,A,0,25   B 0  1    RT 60
14 Green_Pict,StimC,B,0,25   B 1  0    RT 199
15 CirclePict,StimA,B,25,0   A 0  1    RT 166
16 SticksPict,StimB,A,25,0   A 1  0    RT 143
17 Worms_Pict,StimD,B,0,25   A 0  0    RT 118
18 SticksPict,StimB,B,25,0   A 0  0    RT 90
19 Worms_Pict,StimD,B,0,25   B 1  1    RT 86
20 CirclePict,StimA,A,25,0   A 1  1    RT 103
21 SticksPict,StimB,A,25,0   A 1  0    RT 55
22 CirclePict,StimA,A,25,0   A 1  1    RT 96
23 CirclePict,StimA,A,25,0   A 1  1    RT 45
24 Worms_Pict,StimD,B,0,25   B 1  1    RT 109
25 Green_Pict,StimC,A,0,25   A 1  1    RT 277
26 SticksPict,StimB,B,25,0   A 0  0    RT 59
27 SticksPict,StimB,B,25,0   A 0  0    RT 33
28 CirclePict,StimA,A,25,0   A 1  1    RT 41
29 SticksPict,StimB,B,25,0   A 0  0    RT 39
30 SticksPict,StimB,B,25,0   A 0  0    RT 29
31 Green_Pict,StimC,A,0,25   B 0  0    RT 254
32 Green_Pict,StimC,A,0,25   A 1  1    RT 40
33 CirclePict,StimA,B,25,0   A 0  1    RT 46
34 CirclePict,StimA,A,25,0   A 1  1    RT 27
35 Green_Pict,StimC,A,0,25   A 1  1    RT 84
36 Green_Pict,StimC,A,0,25   A 1  1    RT 30
37 Green_Pict,StimC,B,0,25   A 0  1    RT 84
38 CirclePict,StimA,A,25,0   A 1  1    RT 92
39 SticksPict,StimB,B,25,0   A 0  0    RT 117
40 CirclePict,StimA,A,25,0   A 1  1    RT 56
41 CirclePict,StimA,A,25,0   A 1  1    RT 91
42 Green_Pict,StimC,A,0,25   A 1  1    RT 93
43 SticksPict,StimB,B,25,0   A 0  0    RT 50
44 Worms_Pict,StimD,B,0,25   B 1  1    RT 81
45 Green_Pict,StimC,A,0,25   A 1  1    RT 91
46 CirclePict,StimA,A,25,0   A 1  1    RT 41
47 CirclePict,StimA,A,25,0   A 1  1    RT 48
48 Green_Pict,StimC,B,0,25   A 0  1    RT 104
49 SticksPict,StimB,B,25,0   A 0  0    RT 53
50 CirclePict,StimA,A,25,0   A 1  1    RT 39
51 SticksPict,StimB,B,25,0   A 0  0    RT 41
52 Green_Pict,StimC,B,0,25   B 1  0    RT 45
53 Worms_Pict,StimD,B,0,25   B 1  1    RT 93
54 Worms_Pict,StimD,A,0,25   B 0  1    RT 49
55 Worms_Pict,StimD,B,0,25   A 0  0    RT 43
56 SticksPict,StimB,A,25,0   A 1  0    RT 62
57 Worms_Pict,StimD,A,0,25   B 0  1    RT 123
58 CirclePict,StimA,B,25,0   A 0  1    RT 37
59 Green_Pict,StimC,A,0,25   B 0  0    RT 101
60 Worms_Pict,StimD,B,0,25   B 1  1    RT 67
61 SticksPict,StimB,B,25,0   A 0  0    RT 39
62 CirclePict,StimA,A,25,0   A 1  1    RT 36
63 CirclePict,StimA,A,25,0   A 1  1    RT 33
64 Worms_Pict,StimD,B,0,25   A 0  0    RT 75
65 Worms_Pict,StimD,B,0,25   B 1  1    RT 47
66 SticksPict,StimB,A,25,0   A 1  0    RT 39
67 SticksPict,StimB,B,25,0   A 0  0    RT 39
68 Worms_Pict,StimD,B,0,25   B 1  1    RT 95
69 SticksPict,StimB,B,25,0   A 0  0    RT 39
70 Green_Pict,StimC,A,0,25   B 0  0    RT 208
71 CirclePict,StimA,B,25,0   A 0  1    RT 39
72 SticksPict,StimB,B,25,0   A 0  0    RT 35
73 Green_Pict,StimC,A,0,25   A 1  1    RT 81
74 Green_Pict,StimC,A,0,25   A 1  1    RT 33
75 CirclePict,StimA,A,25,0   A 1  1    RT 41
76 CirclePict,StimA,A,25,0   A 1  1    RT 29
77 Green_Pict,StimC,A,0,25   A 1  1    RT 72
78 SticksPict,StimB,B,25,0   A 0  0    RT 35
79 Worms_Pict,StimD,B,0,25   A 0  0    RT 146
80 Green_Pict,StimC,A,0,25   A 1  1    RT 35
81 SticksPict,StimB,B,25,0   A 0  0    RT 33
82 Worms_Pict,StimD,B,0,25   B 1  1    RT 41
83 SticksPict,StimB,B,25,0   A 0  0    RT 42
84 Worms_Pict,StimD,A,0,25   B 0  1    RT 61
85 SticksPict,StimB,A,25,0   A 1  0    RT 91
86 SticksPict,StimB,B,25,0   A 0  0    RT 35
87 SticksPict,StimB,B,25,0   A 0  0    RT 40
88 Green_Pict,StimC,B,0,25   A 0  1    RT 112
89 Green_Pict,StimC,A,0,25   B 0  0    RT 45
90 CirclePict,StimA,A,25,0   A 1  1    RT 69
91 Green_Pict,StimC,A,0,25   B 0  0    RT 55
92 CirclePict,StimA,B,25,0   A 0  1    RT 41
93 Worms_Pict,StimD,A,0,25   A 1  0    RT 107
94 Worms_Pict,StimD,B,0,25   A 0  0    RT 34
95 SticksPict,StimB,A,25,0   A 1  0    RT 58
96 Worms_Pict,StimD,B,0,25   B 1  1    RT 62
97 Worms_Pict,StimD,B,0,25   B 1  1    RT 44
98 CirclePict,StimA,B,25,0   A 0  1    RT 174
99 SticksPict,StimB,B,25,0   A 0  0    RT 43
100 Green_Pict,StimC,A,0,25   A 1  1    RT 106
101 Green_Pict,StimC,A,0,25   A 1  1    RT 51
102 Green_Pict,StimC,A,0,25   A 1  1    RT 39
103 Green_Pict,StimC,A,0,25   A 1  1    RT 38
104 CirclePict,StimA,A,25,0   A 1  1    RT 45
105 CirclePict,StimA,A,25,0   A 1  1    RT 31
106 Worms_Pict,StimD,B,0,25   A 0  0    RT 164
107 Worms_Pict,StimD,B,0,25   B 1  1    RT 110
108 Green_Pict,StimC,A,0,25   A 1  1    RT 55
109 CirclePict,StimA,A,25,0   A 1  1    RT 36
110 Worms_Pict,StimD,B,0,25   B 1  1    RT 72
111 CirclePict,StimA,A,25,0   A 1  1    RT 31
112 Green_Pict,StimC,B,0,25   A 0  1    RT 81
113 Green_Pict,StimC,A,0,25   B 0  0    RT 58
114 CirclePict,StimA,A,25,0   A 1  1    RT 39
115 SticksPict,StimB,B,25,0   A 0  0    RT 35
116 SticksPict,StimB,B,25,0   A 0  0    RT 26
117 SticksPict,StimB,B,25,0   A 0  0    RT 27
118 CirclePict,StimA,A,25,0   A 1  1    RT 36
119 CirclePict,StimA,A,25,0   A 1  1    RT 28
120 Worms_Pict,StimD,B,0,25   B 1  1    RT 57
121 Green_Pict,StimC,B,0,25   B 1  0    RT 76
122 Worms_Pict,StimD,B,0,25   B 1  1    RT 50
123 Worms_Pict,StimD,B,0,25   B 1  1    RT 36
124 Worms_Pict,StimD,B,0,25   B 1  1    RT 34
125 CirclePict,StimA,B,25,0   A 0  1    RT 54
126 Green_Pict,StimC,A,0,25   B 0  0    RT 125
127 CirclePict,StimA,A,25,0   A 1  1    RT 34
128 SticksPict,StimB,B,25,0   A 0  0    RT 36
129 Worms_Pict,StimD,B,0,25   B 1  1    RT 68
130 SticksPict,StimB,B,25,0   A 0  0    RT 36
131 CirclePict,StimA,A,25,0   A 1  1    RT 29
132 SticksPict,StimB,B,25,0   A 0  0    RT 34
133 SticksPict,StimB,B,25,0   A 0  0    RT 32
134 Green_Pict,StimC,A,0,25   B 0  0    RT 81
135 SticksPict,StimB,B,25,0   A 0  0    RT 35
136 CirclePict,StimA,A,25,0   A 1  1    RT 29
137 Green_Pict,StimC,A,0,25   A 1  1    RT 52
138 CirclePict,StimA,A,25,0   A 1  1    RT 40
139 Worms_Pict,StimD,B,0,25   B 1  1    RT 123
140 Worms_Pict,StimD,B,0,25   B 1  1    RT 55
141 Green_Pict,StimC,A,0,25   A 1  1    RT 49
142 SticksPict,StimB,B,25,0   A 0  0    RT 41
143 SticksPict,StimB,A,25,0   A 1  0    RT 28
144 Worms_Pict,StimD,A,0,25   B 0  1    RT 52
145 CirclePict,StimA,A,25,0   A 1  1    RT 34
146 Green_Pict,StimC,A,0,25   A 1  1    RT 150
147 Green_Pict,StimC,A,0,25   A 1  1    RT 30
148 CirclePict,StimA,A,25,0   A 1  1    RT 33
149 SticksPict,StimB,B,25,0   A 0  0    RT 31
150 CirclePict,StimA,A,25,0   A 1  1    RT 29
151 Worms_Pict,StimD,A,0,25   A 1  0    RT 165
152 CirclePict,StimA,A,25,0   A 1  1    RT 32
153 Worms_Pict,StimD,B,0,25   A 0  0    RT 24
154 Green_Pict,StimC,B,0,25   A 0  1    RT 74
155 Green_Pict,StimC,A,0,25   B 0  0    RT 36
156 Worms_Pict,StimD,B,0,25   B 1  1    RT 173
157 SticksPict,StimB,B,25,0   A 0  0    RT 63
158 CirclePict,StimA,B,25,0   A 0  1    RT 35
159 Green_Pict,StimC,A,0,25   A 1  1    RT 71
160 SticksPict,StimB,A,25,0   A 1  0    RT 38


Total AB Correct = 39 (48.75% correct)
Total CD Correct = 48 (60% correct)
Total AB optimal = 39 (48.75% optimal)
Total CD optimal = 54 (67.5% optimal)
Total points = 675
```
