# Supplementary material for: Identifying the Basal Ganglia Network Model Markers for Medication-Induced Impulsivity in Parkinson's Disease Patients
Source: PLoS One. 2015 Jun 4;10(6):e0127542. doi: 10.1371/journal.pone.0127542 (PMC4456385; doi:10.1371/journal.pone.0127542)
Supplement: S4 File — (ZIP) [file pone.0127542.s004.zip › SMat/Quarters_PDoff1.doc]

```
QuartersCF4
Subject ID: Off 1
 Stimuli:
A = Green_Pict B= SticksPict
C = Worms_Pict C= CirclePict
Stim A is class A 80% of trials; Stim B is class A 20% of trials
    win = 25 points, lose is 0
Stim C is class A 80% of trials; Stim D is class A 20% of trials
    win = 0, lose = 25 points


Key: A/B is subj response, 0/1 is correct?, 0/1 is optimal?
1 CirclePict,StimD,A,0,25   B 0  1    RT 44
2 Worms_Pict,StimC,B,0,25   A 0  1    RT 61
3 CirclePict,StimD,B,0,25   A 0  0    RT 330
4 Worms_Pict,StimC,A,0,25   B 0  0    RT 48
5 Green_Pict,StimA,B,25,0   A 0  1    RT 112
6 SticksPict,StimB,B,25,0   B 1  1    RT 40
7 SticksPict,StimB,B,25,0   B 1  1    RT 85
8 SticksPict,StimB,B,25,0   B 1  1    RT 59
9 SticksPict,StimB,B,25,0   B 1  1    RT 45
10 CirclePict,StimD,B,0,25   A 0  0    RT 72
11 CirclePict,StimD,B,0,25   B 1  1    RT 44
12 Worms_Pict,StimC,A,0,25   A 1  1    RT 98
13 SticksPict,StimB,B,25,0   A 0  0    RT 102
14 SticksPict,StimB,B,25,0   A 0  0    RT 74
15 Green_Pict,StimA,A,25,0   B 0  0    RT 48
16 SticksPict,StimB,B,25,0   A 0  0    RT 52
17 CirclePict,StimD,B,0,25   B 1  1    RT 46
18 Green_Pict,StimA,A,25,0   B 0  0    RT 53
19 Worms_Pict,StimC,A,0,25   B 0  0    RT 67
20 CirclePict,StimD,B,0,25   B 1  1    RT 32
21 Worms_Pict,StimC,B,0,25   A 0  1    RT 64
22 CirclePict,StimD,B,0,25   B 1  1    RT 39
23 CirclePict,StimD,B,0,25   B 1  1    RT 35
24 Worms_Pict,StimC,A,0,25   B 0  0    RT 32
25 Green_Pict,StimA,B,25,0   A 0  1    RT 63
26 Worms_Pict,StimC,A,0,25   B 0  0    RT 58
27 Green_Pict,StimA,A,25,0   B 0  0    RT 46
28 Green_Pict,StimA,A,25,0   B 0  0    RT 220
29 CirclePict,StimD,B,0,25   B 1  1    RT 57
30 Worms_Pict,StimC,A,0,25   A 1  1    RT 56
31 Green_Pict,StimA,A,25,0   B 0  0    RT 48
32 Worms_Pict,StimC,A,0,25   A 1  1    RT 30
33 CirclePict,StimD,A,0,25   B 0  1    RT 29
34 SticksPict,StimB,B,25,0   A 0  0    RT 81
35 Green_Pict,StimA,A,25,0   A 1  1    RT 44
36 Worms_Pict,StimC,A,0,25   B 0  0    RT 33
37 SticksPict,StimB,A,25,0   A 1  0    RT 86
38 Green_Pict,StimA,A,25,0   B 0  0    RT 49
39 SticksPict,StimB,A,25,0   A 1  0    RT 121
40 Green_Pict,StimA,A,25,0   B 0  0    RT 46
41 Worms_Pict,StimC,A,0,25   B 0  0    RT 63
42 SticksPict,StimB,B,25,0   A 0  0    RT 85
43 CirclePict,StimD,A,0,25   B 0  1    RT 38
44 Green_Pict,StimA,A,25,0   A 1  1    RT 55
45 Worms_Pict,StimC,A,0,25   B 0  0    RT 52
46 Green_Pict,StimA,B,25,0   A 0  1    RT 49
47 Green_Pict,StimA,A,25,0   A 1  1    RT 30
48 CirclePict,StimD,B,0,25   B 1  1    RT 48
49 SticksPict,StimB,B,25,0   A 0  0    RT 208
50 SticksPict,StimB,A,25,0   A 1  0    RT 36
51 SticksPict,StimB,B,25,0   A 0  0    RT 26
52 SticksPict,StimB,B,25,0   A 0  0    RT 27
53 CirclePict,StimD,A,0,25   B 0  1    RT 37
54 Worms_Pict,StimC,A,0,25   A 1  1    RT 78
55 Green_Pict,StimA,A,25,0   A 1  1    RT 37
56 Worms_Pict,StimC,B,0,25   A 0  1    RT 54
57 CirclePict,StimD,B,0,25   B 1  1    RT 44
58 CirclePict,StimD,B,0,25   B 1  1    RT 28
59 Worms_Pict,StimC,A,0,25   A 1  1    RT 48
60 CirclePict,StimD,B,0,25   B 1  1    RT 28
61 SticksPict,StimB,B,25,0   A 0  0    RT 139
62 Green_Pict,StimA,B,25,0   A 0  1    RT 64
63 Worms_Pict,StimC,A,0,25   A 1  1    RT 74
64 Green_Pict,StimA,A,25,0   A 1  1    RT 99
65 Green_Pict,StimA,A,25,0   A 1  1    RT 28
66 Worms_Pict,StimC,B,0,25   A 0  1    RT 85
67 CirclePict,StimD,B,0,25   B 1  1    RT 32
68 Green_Pict,StimA,A,25,0   A 1  1    RT 46
69 CirclePict,StimD,B,0,25   B 1  1    RT 34
70 Worms_Pict,StimC,A,0,25   B 0  0    RT 119
71 Worms_Pict,StimC,A,0,25   A 1  1    RT 48
72 Worms_Pict,StimC,A,0,25   A 1  1    RT 24
73 Green_Pict,StimA,A,25,0   A 1  1    RT 49
74 SticksPict,StimB,B,25,0   A 0  0    RT 56
75 SticksPict,StimB,B,25,0   A 0  0    RT 27
76 CirclePict,StimD,B,0,25   B 1  1    RT 45
77 Green_Pict,StimA,A,25,0   A 1  1    RT 45
78 SticksPict,StimB,B,25,0   A 0  0    RT 51
79 CirclePict,StimD,B,0,25   B 1  1    RT 37
80 SticksPict,StimB,A,25,0   A 1  0    RT 43
81 Green_Pict,StimA,A,25,0   A 1  1    RT 37
82 Worms_Pict,StimC,A,0,25   A 1  1    RT 61
83 CirclePict,StimD,A,0,25   B 0  1    RT 36
84 Worms_Pict,StimC,A,0,25   A 1  1    RT 40
85 CirclePict,StimD,A,0,25   B 0  1    RT 40
86 Green_Pict,StimA,A,25,0   A 1  1    RT 86
87 Green_Pict,StimA,A,25,0   A 1  1    RT 38
88 SticksPict,StimB,B,25,0   A 0  0    RT 92
89 SticksPict,StimB,B,25,0   A 0  0    RT 37
90 SticksPict,StimB,B,25,0   A 0  0    RT 30
91 CirclePict,StimD,B,0,25   B 1  1    RT 35
92 Green_Pict,StimA,A,25,0   B 0  0    RT 74
93 Worms_Pict,StimC,A,0,25   A 1  1    RT 69
94 Green_Pict,StimA,A,25,0   B 0  0    RT 78
95 SticksPict,StimB,B,25,0   A 0  0    RT 60
96 Worms_Pict,StimC,B,0,25   A 0  1    RT 112
97 CirclePict,StimD,B,0,25   B 1  1    RT 30
98 CirclePict,StimD,B,0,25   B 1  1    RT 32
99 Worms_Pict,StimC,B,0,25   B 1  0    RT 75
100 Worms_Pict,StimC,A,0,25   B 0  0    RT 29
101 SticksPict,StimB,B,25,0   A 0  0    RT 77
102 Worms_Pict,StimC,A,0,25   A 1  1    RT 54
103 SticksPict,StimB,B,25,0   B 1  1    RT 126
104 CirclePict,StimD,B,0,25   B 1  1    RT 39
105 Green_Pict,StimA,B,25,0   B 1  0    RT 56
106 SticksPict,StimB,A,25,0   A 1  0    RT 93
107 CirclePict,StimD,B,0,25   B 1  1    RT 34
108 Worms_Pict,StimC,A,0,25   A 1  1    RT 88
109 SticksPict,StimB,B,25,0   A 0  0    RT 111
110 Worms_Pict,StimC,A,0,25   A 1  1    RT 56
111 CirclePict,StimD,B,0,25   B 1  1    RT 36
112 CirclePict,StimD,B,0,25   B 1  1    RT 47
113 SticksPict,StimB,B,25,0   A 0  0    RT 57
114 Green_Pict,StimA,A,25,0   B 0  0    RT 69
115 Green_Pict,StimA,A,25,0   B 0  0    RT 48
116 Worms_Pict,StimC,A,0,25   A 1  1    RT 65
117 CirclePict,StimD,B,0,25   B 1  1    RT 37
118 SticksPict,StimB,A,25,0   B 0  1    RT 54
119 Green_Pict,StimA,B,25,0   B 1  0    RT 47
120 Green_Pict,StimA,A,25,0   B 0  0    RT 126
121 Worms_Pict,StimC,A,0,25   A 1  1    RT 75
122 Green_Pict,StimA,A,25,0   B 0  0    RT 51
123 Green_Pict,StimA,A,25,0   B 0  0    RT 42
124 Green_Pict,StimA,A,25,0   B 0  0    RT 38
125 Worms_Pict,StimC,B,0,25   A 0  1    RT 45
126 CirclePict,StimD,A,0,25   B 0  1    RT 40
127 CirclePict,StimD,B,0,25   B 1  1    RT 30
128 SticksPict,StimB,B,25,0   A 0  0    RT 39
129 CirclePict,StimD,B,0,25   B 1  1    RT 35
130 Worms_Pict,StimC,A,0,25   A 1  1    RT 35
131 SticksPict,StimB,B,25,0   B 1  1    RT 77
132 Worms_Pict,StimC,A,0,25   A 1  1    RT 38
133 CirclePict,StimD,B,0,25   B 1  1    RT 39
134 Green_Pict,StimA,B,25,0   B 1  0    RT 48
135 Green_Pict,StimA,A,25,0   A 1  1    RT 69
136 SticksPict,StimB,B,25,0   B 1  1    RT 53
137 CirclePict,StimD,B,0,25   B 1  1    RT 40
138 CirclePict,StimD,B,0,25   B 1  1    RT 33
139 Worms_Pict,StimC,A,0,25   A 1  1    RT 48
140 SticksPict,StimB,B,25,0   B 1  1    RT 116
141 Green_Pict,StimA,A,25,0   A 1  1    RT 46
142 SticksPict,StimB,B,25,0   A 0  0    RT 75
143 SticksPict,StimB,A,25,0   A 1  0    RT 30
144 Worms_Pict,StimC,A,0,25   B 0  0    RT 91
145 SticksPict,StimB,A,25,0   B 0  1    RT 54
146 Green_Pict,StimA,A,25,0   B 0  0    RT 50
147 SticksPict,StimB,B,25,0   A 0  0    RT 55
148 SticksPict,StimB,B,25,0   A 0  0    RT 36
149 Worms_Pict,StimC,B,0,25   B 1  0    RT 75
150 Worms_Pict,StimC,A,0,25   B 0  0    RT 49
151 CirclePict,StimD,B,0,25   B 1  1    RT 37
152 Green_Pict,StimA,A,25,0   A 1  1    RT 140
153 Green_Pict,StimA,B,25,0   A 0  1    RT 89
154 SticksPict,StimB,B,25,0   A 0  0    RT 51
155 Green_Pict,StimA,A,25,0   A 1  1    RT 93
156 CirclePict,StimD,B,0,25   B 1  1    RT 41
157 Worms_Pict,StimC,A,0,25   A 1  1    RT 70
158 CirclePict,StimD,A,0,25   B 0  1    RT 33
159 CirclePict,StimD,B,0,25   B 1  1    RT 31
160 Worms_Pict,StimC,A,0,25   A 1  1    RT 40


Total AB Correct = 33 (41.25% correct)
Total CD Correct = 53 (66.25% correct)
Total AB optimal = 31 (38.75% optimal)
Total CD optimal = 65 (81.25% optimal)
Total points = 650
```
