# Supplementary material for: Identifying the Basal Ganglia Network Model Markers for Medication-Induced Impulsivity in Parkinson's Disease Patients
Source: PLoS One. 2015 Jun 4;10(6):e0127542. doi: 10.1371/journal.pone.0127542 (PMC4456385; doi:10.1371/journal.pone.0127542)
Supplement: S4 File — (ZIP) [file pone.0127542.s004.zip › SMat/Quarters_PDoff2.doc]

```
QuartersCF4
Subject ID: Off 2
 Stimuli:
A = Green_Pict B= Worms_Pict
C = CirclePict C= SticksPict
Stim A is class A 80% of trials; Stim B is class A 20% of trials
    win = 25 points, lose is 0
Stim C is class A 80% of trials; Stim D is class A 20% of trials
    win = 0, lose = 25 points


Key: A/B is subj response, 0/1 is correct?, 0/1 is optimal?
1 Worms_Pict,StimB,A,25,0   B 0  1    RT 78
2 CirclePict,StimC,A,0,25   B 0  0    RT 47
3 SticksPict,StimD,A,0,25   A 1  0    RT 51
4 CirclePict,StimC,A,0,25   A 1  1    RT 91
5 Worms_Pict,StimB,B,25,0   A 0  0    RT 44
6 Worms_Pict,StimB,A,25,0   B 0  1    RT 29
7 Worms_Pict,StimB,B,25,0   B 1  1    RT 60
8 Green_Pict,StimA,A,25,0   A 1  1    RT 42
9 Worms_Pict,StimB,B,25,0   A 0  0    RT 67
10 Worms_Pict,StimB,B,25,0   A 0  0    RT 28
11 Green_Pict,StimA,A,25,0   B 0  0    RT 68
12 Worms_Pict,StimB,B,25,0   A 0  0    RT 57
13 Worms_Pict,StimB,B,25,0   A 0  0    RT 26
14 Green_Pict,StimA,A,25,0   B 0  0    RT 86
15 CirclePict,StimC,A,0,25   A 1  1    RT 34
16 CirclePict,StimC,A,0,25   A 1  1    RT 94
17 SticksPict,StimD,B,0,25   B 1  1    RT 42
18 CirclePict,StimC,A,0,25   B 0  0    RT 24
19 SticksPict,StimD,B,0,25   A 0  0    RT 70
20 Worms_Pict,StimB,B,25,0   A 0  0    RT 60
21 CirclePict,StimC,A,0,25   B 0  0    RT 63
22 SticksPict,StimD,B,0,25   A 0  0    RT 76
23 SticksPict,StimD,B,0,25   B 1  1    RT 94
24 CirclePict,StimC,B,0,25   A 0  1    RT 51
25 Green_Pict,StimA,A,25,0   A 1  1    RT 56
26 SticksPict,StimD,B,0,25   B 1  1    RT 45
27 CirclePict,StimC,A,0,25   A 1  1    RT 105
28 Green_Pict,StimA,A,25,0   A 1  1    RT 58
29 Green_Pict,StimA,A,25,0   A 1  1    RT 81
30 Green_Pict,StimA,B,25,0   A 0  1    RT 33
31 SticksPict,StimD,B,0,25   B 1  1    RT 32
32 SticksPict,StimD,B,0,25   B 1  1    RT 31
33 Green_Pict,StimA,B,25,0   B 1  0    RT 75
34 SticksPict,StimD,A,0,25   B 0  1    RT 28
35 CirclePict,StimC,B,0,25   A 0  1    RT 36
36 CirclePict,StimC,A,0,25   A 1  1    RT 58
37 SticksPict,StimD,B,0,25   B 1  1    RT 35
38 Green_Pict,StimA,A,25,0   A 1  1    RT 43
39 Green_Pict,StimA,A,25,0   B 0  0    RT 89
40 Worms_Pict,StimB,B,25,0   B 1  1    RT 42
41 Green_Pict,StimA,B,25,0   A 0  1    RT 61
42 CirclePict,StimC,A,0,25   A 1  1    RT 48
43 SticksPict,StimD,B,0,25   B 1  1    RT 36
44 Green_Pict,StimA,A,25,0   A 1  1    RT 53
45 Green_Pict,StimA,A,25,0   B 0  0    RT 56
46 SticksPict,StimD,A,0,25   B 0  1    RT 77
47 SticksPict,StimD,B,0,25   B 1  1    RT 26
48 Green_Pict,StimA,A,25,0   B 0  0    RT 63
49 Worms_Pict,StimB,B,25,0   A 0  0    RT 121
50 CirclePict,StimC,A,0,25   A 1  1    RT 58
51 Green_Pict,StimA,A,25,0   A 1  1    RT 58
52 Worms_Pict,StimB,B,25,0   A 0  0    RT 52
53 CirclePict,StimC,A,0,25   A 1  1    RT 24
54 CirclePict,StimC,A,0,25   A 1  1    RT 30
55 Green_Pict,StimA,A,25,0   B 0  0    RT 53
56 Worms_Pict,StimB,A,25,0   A 1  0    RT 102
57 Green_Pict,StimA,A,25,0   B 0  0    RT 46
58 CirclePict,StimC,A,0,25   A 1  1    RT 43
59 Worms_Pict,StimB,B,25,0   B 1  1    RT 44
60 SticksPict,StimD,B,0,25   B 1  1    RT 32
61 Worms_Pict,StimB,A,25,0   A 1  0    RT 40
62 Worms_Pict,StimB,B,25,0   B 1  1    RT 39
63 SticksPict,StimD,B,0,25   A 0  0    RT 22
64 CirclePict,StimC,B,0,25   A 0  1    RT 55
65 SticksPict,StimD,B,0,25   B 1  1    RT 25
66 Worms_Pict,StimB,B,25,0   B 1  1    RT 57
67 SticksPict,StimD,B,0,25   B 1  1    RT 57
68 Green_Pict,StimA,A,25,0   A 1  1    RT 53
69 SticksPict,StimD,B,0,25   B 1  1    RT 40
70 Worms_Pict,StimB,B,25,0   B 1  1    RT 63
71 Green_Pict,StimA,B,25,0   B 1  0    RT 43
72 Worms_Pict,StimB,B,25,0   A 0  0    RT 44
73 CirclePict,StimC,A,0,25   A 1  1    RT 53
74 SticksPict,StimD,B,0,25   B 1  1    RT 35
75 Green_Pict,StimA,A,25,0   B 0  0    RT 74
76 SticksPict,StimD,A,0,25   B 0  1    RT 40
77 CirclePict,StimC,A,0,25   A 1  1    RT 39
78 Worms_Pict,StimB,B,25,0   A 0  0    RT 48
79 CirclePict,StimC,A,0,25   A 1  1    RT 148
80 CirclePict,StimC,B,0,25   A 0  1    RT 33
81 Worms_Pict,StimB,B,25,0   A 0  0    RT 68
82 Green_Pict,StimA,A,25,0   B 0  0    RT 162
83 Worms_Pict,StimB,B,25,0   A 0  0    RT 45
84 Green_Pict,StimA,B,25,0   B 1  0    RT 48
85 SticksPict,StimD,B,0,25   B 1  1    RT 37
86 SticksPict,StimD,B,0,25   B 1  1    RT 28
87 CirclePict,StimC,A,0,25   A 1  1    RT 42
88 Green_Pict,StimA,A,25,0   B 0  0    RT 37
89 Green_Pict,StimA,A,25,0   B 0  0    RT 54
90 CirclePict,StimC,A,0,25   A 1  1    RT 41
91 Worms_Pict,StimB,B,25,0   B 1  1    RT 44
92 Green_Pict,StimA,A,25,0   A 1  1    RT 52
93 Green_Pict,StimA,A,25,0   A 1  1    RT 58
94 Green_Pict,StimA,A,25,0   A 1  1    RT 53
95 CirclePict,StimC,A,0,25   B 0  0    RT 77
96 SticksPict,StimD,B,0,25   B 1  1    RT 41
97 Green_Pict,StimA,A,25,0   A 1  1    RT 138
98 CirclePict,StimC,A,0,25   B 0  0    RT 71
99 Worms_Pict,StimB,A,25,0   B 0  1    RT 63
100 SticksPict,StimD,A,0,25   B 0  1    RT 44
101 SticksPict,StimD,B,0,25   A 0  0    RT 119
102 SticksPict,StimD,B,0,25   B 1  1    RT 42
103 Worms_Pict,StimB,B,25,0   B 1  1    RT 51
104 SticksPict,StimD,B,0,25   B 1  1    RT 35
105 Worms_Pict,StimB,B,25,0   B 1  1    RT 65
106 CirclePict,StimC,A,0,25   A 1  1    RT 70
107 Worms_Pict,StimB,B,25,0   A 0  0    RT 78
108 Green_Pict,StimA,A,25,0   B 0  0    RT 48
109 CirclePict,StimC,B,0,25   A 0  1    RT 46
110 CirclePict,StimC,A,0,25   B 0  0    RT 61
111 CirclePict,StimC,B,0,25   A 0  1    RT 91
112 CirclePict,StimC,A,0,25   A 1  1    RT 41
113 Worms_Pict,StimB,A,25,0   B 0  1    RT 41
114 CirclePict,StimC,A,0,25   A 1  1    RT 28
115 SticksPict,StimD,B,0,25   B 1  1    RT 40
116 Green_Pict,StimA,B,25,0   B 1  0    RT 62
117 SticksPict,StimD,B,0,25   B 1  1    RT 40
118 Worms_Pict,StimB,B,25,0   B 1  1    RT 39
119 SticksPict,StimD,A,0,25   B 0  1    RT 40
120 Worms_Pict,StimB,B,25,0   A 0  0    RT 51
121 Worms_Pict,StimB,B,25,0   A 0  0    RT 27
122 Worms_Pict,StimB,B,25,0   A 0  0    RT 28
123 CirclePict,StimC,A,0,25   A 1  1    RT 59
124 Worms_Pict,StimB,B,25,0   A 0  0    RT 45
125 Green_Pict,StimA,A,25,0   A 1  1    RT 59
126 SticksPict,StimD,A,0,25   B 0  1    RT 59
127 SticksPict,StimD,B,0,25   B 1  1    RT 36
128 Worms_Pict,StimB,B,25,0   A 0  0    RT 35
129 SticksPict,StimD,B,0,25   B 1  1    RT 52
130 Worms_Pict,StimB,A,25,0   A 1  0    RT 29
131 Green_Pict,StimA,A,25,0   B 0  0    RT 79
132 SticksPict,StimD,B,0,25   B 1  1    RT 55
133 Green_Pict,StimA,A,25,0   B 0  0    RT 46
134 SticksPict,StimD,A,0,25   B 0  1    RT 34
135 Worms_Pict,StimB,B,25,0   A 0  0    RT 36
136 SticksPict,StimD,B,0,25   B 1  1    RT 36
137 CirclePict,StimC,A,0,25   A 1  1    RT 110
138 SticksPict,StimD,B,0,25   B 1  1    RT 33
139 SticksPict,StimD,B,0,25   B 1  1    RT 37
140 Green_Pict,StimA,B,25,0   B 1  0    RT 79
141 SticksPict,StimD,B,0,25   B 1  1    RT 31
142 CirclePict,StimC,A,0,25   A 1  1    RT 36
143 Worms_Pict,StimB,B,25,0   B 1  1    RT 54
144 Worms_Pict,StimB,A,25,0   A 1  0    RT 68
145 SticksPict,StimD,B,0,25   B 1  1    RT 28
146 CirclePict,StimC,B,0,25   A 0  1    RT 37
147 CirclePict,StimC,A,0,25   A 1  1    RT 42
148 Green_Pict,StimA,B,25,0   B 1  0    RT 42
149 CirclePict,StimC,A,0,25   A 1  1    RT 39
150 Green_Pict,StimA,A,25,0   B 0  0    RT 49
151 CirclePict,StimC,B,0,25   A 0  1    RT 37
152 CirclePict,StimC,A,0,25   B 0  0    RT 43
153 Green_Pict,StimA,A,25,0   A 1  1    RT 35
154 Green_Pict,StimA,A,25,0   B 0  0    RT 33
155 Green_Pict,StimA,A,25,0   B 0  0    RT 36
156 Worms_Pict,StimB,B,25,0   A 0  0    RT 52
157 CirclePict,StimC,A,0,25   B 0  0    RT 48
158 Green_Pict,StimA,A,25,0   A 1  1    RT 37
159 Worms_Pict,StimB,B,25,0   A 0  0    RT 85
160 CirclePict,StimC,A,0,25   A 1  1    RT 75


Total AB Correct = 36 (45% correct)
Total CD Correct = 53 (66.25% correct)
Total AB optimal = 32 (40% optimal)
Total CD optimal = 67 (83.75% optimal)
Total points = 725
```
