# Supplementary material for: Identifying the Basal Ganglia Network Model Markers for Medication-Induced Impulsivity in Parkinson's Disease Patients
Source: PLoS One. 2015 Jun 4;10(6):e0127542. doi: 10.1371/journal.pone.0127542 (PMC4456385; doi:10.1371/journal.pone.0127542)
Supplement: S4 File — (ZIP) [file pone.0127542.s004.zip › SMat/Quarters_PDoff3.doc]

```
QuartersCF4
Subject ID: Off 3
 Stimuli:
A = SticksPict B= Green_Pict
C = CirclePict C= Worms_Pict
Stim A is class A 80% of trials; Stim B is class A 20% of trials
    win = 25 points, lose is 0
Stim C is class A 80% of trials; Stim D is class A 20% of trials
    win = 0, lose = 25 points


Key: A/B is subj response, 0/1 is correct?, 0/1 is optimal?
1 Worms_Pict,StimD,A,0,25   B 0  1    RT 111
2 Green_Pict,StimB,B,25,0   A 0  0    RT 89
3 CirclePict,StimC,A,0,25   B 0  0    RT 271
4 CirclePict,StimC,A,0,25   A 1  1    RT 95
5 CirclePict,StimC,A,0,25   A 1  1    RT 91
6 SticksPict,StimA,A,25,0   B 0  0    RT 108
7 Green_Pict,StimB,B,25,0   A 0  0    RT 71
8 SticksPict,StimA,A,25,0   B 0  0    RT 180
9 Green_Pict,StimB,B,25,0   A 0  0    RT 49
10 Green_Pict,StimB,B,25,0   A 0  0    RT 56
11 Green_Pict,StimB,B,25,0   A 0  0    RT 47
12 Green_Pict,StimB,B,25,0   A 0  0    RT 52
13 SticksPict,StimA,B,25,0   B 1  0    RT 36
14 SticksPict,StimA,A,25,0   B 0  0    RT 61
15 Worms_Pict,StimD,B,0,25   B 1  1    RT 82
16 Worms_Pict,StimD,B,0,25   B 1  1    RT 78
17 CirclePict,StimC,A,0,25   A 1  1    RT 40
18 SticksPict,StimA,A,25,0   B 0  0    RT 68
19 Worms_Pict,StimD,A,0,25   A 1  0    RT 36
20 Green_Pict,StimB,A,25,0   A 1  0    RT 35
21 CirclePict,StimC,B,0,25   B 1  0    RT 56
22 CirclePict,StimC,A,0,25   B 0  0    RT 30
23 Worms_Pict,StimD,B,0,25   A 0  0    RT 52
24 Worms_Pict,StimD,B,0,25   B 1  1    RT 43
25 SticksPict,StimA,A,25,0   B 0  0    RT 45
26 SticksPict,StimA,A,25,0   B 0  0    RT 33
27 Worms_Pict,StimD,B,0,25   B 1  1    RT 85
28 CirclePict,StimC,A,0,25   A 1  1    RT 143
29 Green_Pict,StimB,B,25,0   A 0  0    RT 38
30 CirclePict,StimC,A,0,25   B 0  0    RT 42
31 Green_Pict,StimB,B,25,0   A 0  0    RT 41
32 CirclePict,StimC,A,0,25   A 1  1    RT 68
33 Worms_Pict,StimD,B,0,25   A 0  0    RT 133
34 SticksPict,StimA,A,25,0   B 0  0    RT 40
35 Green_Pict,StimB,A,25,0   A 1  0    RT 38
36 CirclePict,StimC,B,0,25   B 1  0    RT 81
37 Worms_Pict,StimD,B,0,25   B 1  1    RT 81
38 SticksPict,StimA,A,25,0   B 0  0    RT 40
39 SticksPict,StimA,B,25,0   B 1  0    RT 26
40 Worms_Pict,StimD,B,0,25   B 1  1    RT 79
41 Worms_Pict,StimD,B,0,25   B 1  1    RT 32
42 SticksPict,StimA,A,25,0   B 0  0    RT 43
43 CirclePict,StimC,A,0,25   B 0  0    RT 90
44 Green_Pict,StimB,B,25,0   A 0  0    RT 37
45 CirclePict,StimC,B,0,25   A 0  1    RT 30
46 CirclePict,StimC,B,0,25   A 0  1    RT 30
47 Green_Pict,StimB,B,25,0   A 0  0    RT 35
48 SticksPict,StimA,A,25,0   B 0  0    RT 34
49 SticksPict,StimA,B,25,0   B 1  0    RT 22
50 CirclePict,StimC,A,0,25   A 1  1    RT 161
51 CirclePict,StimC,A,0,25   A 1  1    RT 26
52 Green_Pict,StimB,B,25,0   A 0  0    RT 36
53 Worms_Pict,StimD,B,0,25   B 1  1    RT 72
54 Worms_Pict,StimD,B,0,25   B 1  1    RT 24
55 Worms_Pict,StimD,B,0,25   B 1  1    RT 26
56 Worms_Pict,StimD,B,0,25   B 1  1    RT 24
57 SticksPict,StimA,A,25,0   B 0  0    RT 85
58 CirclePict,StimC,A,0,25   A 1  1    RT 172
59 Green_Pict,StimB,A,25,0   A 1  0    RT 38
60 SticksPict,StimA,B,25,0   B 1  0    RT 54
61 Worms_Pict,StimD,A,0,25   B 0  1    RT 65
62 CirclePict,StimC,A,0,25   A 1  1    RT 221
63 Green_Pict,StimB,B,25,0   A 0  0    RT 41
64 CirclePict,StimC,A,0,25   B 0  0    RT 50
65 Worms_Pict,StimD,B,0,25   B 1  1    RT 78
66 SticksPict,StimA,A,25,0   B 0  0    RT 54
67 Green_Pict,StimB,B,25,0   A 0  0    RT 38
68 Worms_Pict,StimD,B,0,25   B 1  1    RT 49
69 Green_Pict,StimB,A,25,0   A 1  0    RT 26
70 SticksPict,StimA,A,25,0   B 0  0    RT 61
71 SticksPict,StimA,A,25,0   B 0  0    RT 35
72 CirclePict,StimC,A,0,25   A 1  1    RT 62
73 Green_Pict,StimB,B,25,0   A 0  0    RT 39
74 SticksPict,StimA,A,25,0   B 0  0    RT 36
75 SticksPict,StimA,A,25,0   B 0  0    RT 47
76 Green_Pict,StimB,B,25,0   A 0  0    RT 32
77 CirclePict,StimC,A,0,25   A 1  1    RT 61
78 Worms_Pict,StimD,A,0,25   B 0  1    RT 32
79 Worms_Pict,StimD,B,0,25   A 0  0    RT 84
80 Green_Pict,StimB,B,25,0   A 0  0    RT 36
81 CirclePict,StimC,A,0,25   A 1  1    RT 52
82 Green_Pict,StimB,B,25,0   A 0  0    RT 34
83 Green_Pict,StimB,A,25,0   A 1  0    RT 51
84 Worms_Pict,StimD,B,0,25   B 1  1    RT 78
85 Green_Pict,StimB,B,25,0   A 0  0    RT 35
86 Worms_Pict,StimD,A,0,25   B 0  1    RT 85
87 CirclePict,StimC,A,0,25   A 1  1    RT 57
88 SticksPict,StimA,A,25,0   B 0  0    RT 44
89 SticksPict,StimA,A,25,0   B 0  0    RT 28
90 CirclePict,StimC,A,0,25   A 1  1    RT 63
91 SticksPict,StimA,A,25,0   B 0  0    RT 65
92 Worms_Pict,StimD,B,0,25   B 1  1    RT 109
93 CirclePict,StimC,A,0,25   A 1  1    RT 51
94 Green_Pict,StimB,A,25,0   A 1  0    RT 34
95 Green_Pict,StimB,B,25,0   A 0  0    RT 31
96 Worms_Pict,StimD,B,0,25   B 1  1    RT 52
97 SticksPict,StimA,B,25,0   B 1  0    RT 61
98 Worms_Pict,StimD,B,0,25   B 1  1    RT 50
99 Green_Pict,StimB,B,25,0   A 0  0    RT 42
100 SticksPict,StimA,A,25,0   B 0  0    RT 43
101 SticksPict,StimA,A,25,0   B 0  0    RT 34
102 Green_Pict,StimB,B,25,0   A 0  0    RT 40
103 Worms_Pict,StimD,B,0,25   B 1  1    RT 37
104 Worms_Pict,StimD,B,0,25   B 1  1    RT 33
105 SticksPict,StimA,A,25,0   B 0  0    RT 41
106 CirclePict,StimC,A,0,25   A 1  1    RT 41
107 Green_Pict,StimB,B,25,0   A 0  0    RT 39
108 CirclePict,StimC,B,0,25   A 0  1    RT 64
109 Worms_Pict,StimD,A,0,25   B 0  1    RT 76
110 Green_Pict,StimB,B,25,0   A 0  0    RT 95
111 Worms_Pict,StimD,B,0,25   A 0  0    RT 33
112 Green_Pict,StimB,B,25,0   A 0  0    RT 35
113 CirclePict,StimC,B,0,25   A 0  1    RT 56
114 CirclePict,StimC,A,0,25   A 1  1    RT 28
115 CirclePict,StimC,A,0,25   A 1  1    RT 24
116 SticksPict,StimA,A,25,0   B 0  0    RT 43
117 Worms_Pict,StimD,B,0,25   B 1  1    RT 61
118 SticksPict,StimA,B,25,0   B 1  0    RT 51
119 SticksPict,StimA,A,25,0   B 0  0    RT 53
120 CirclePict,StimC,A,0,25   A 1  1    RT 42
121 CirclePict,StimC,A,0,25   A 1  1    RT 32
122 Worms_Pict,StimD,B,0,25   B 1  1    RT 36
123 Green_Pict,StimB,B,25,0   A 0  0    RT 36
124 SticksPict,StimA,B,25,0   B 1  0    RT 36
125 Worms_Pict,StimD,B,0,25   B 1  1    RT 46
126 CirclePict,StimC,A,0,25   A 1  1    RT 46
127 SticksPict,StimA,A,25,0   B 0  0    RT 36
128 SticksPict,StimA,A,25,0   B 0  0    RT 29
129 CirclePict,StimC,A,0,25   A 1  1    RT 132
130 SticksPict,StimA,A,25,0   B 0  0    RT 37
131 Green_Pict,StimB,B,25,0   A 0  0    RT 41
132 SticksPict,StimA,A,25,0   B 0  0    RT 47
133 CirclePict,StimC,A,0,25   A 1  1    RT 35
134 CirclePict,StimC,A,0,25   A 1  1    RT 31
135 Worms_Pict,StimD,B,0,25   B 1  1    RT 36
136 Green_Pict,StimB,B,25,0   A 0  0    RT 35
137 Worms_Pict,StimD,B,0,25   B 1  1    RT 35
138 CirclePict,StimC,A,0,25   A 1  1    RT 34
139 Green_Pict,StimB,A,25,0   A 1  0    RT 40
140 Green_Pict,StimB,B,25,0   A 0  0    RT 25
141 Worms_Pict,StimD,B,0,25   B 1  1    RT 35
142 SticksPict,StimA,B,25,0   B 1  0    RT 37
143 Green_Pict,StimB,B,25,0   A 0  0    RT 41
144 Worms_Pict,StimD,A,0,25   B 0  1    RT 39
145 Worms_Pict,StimD,A,0,25   B 0  1    RT 51
146 CirclePict,StimC,B,0,25   A 0  1    RT 30
147 SticksPict,StimA,A,25,0   B 0  0    RT 85
148 Green_Pict,StimB,B,25,0   A 0  0    RT 34
149 SticksPict,StimA,A,25,0   B 0  0    RT 42
150 SticksPict,StimA,A,25,0   B 0  0    RT 27
151 CirclePict,StimC,A,0,25   A 1  1    RT 29
152 CirclePict,StimC,A,0,25   A 1  1    RT 23
153 Worms_Pict,StimD,B,0,25   B 1  1    RT 34
154 Green_Pict,StimB,A,25,0   A 1  0    RT 40
155 Green_Pict,StimB,B,25,0   A 0  0    RT 35
156 CirclePict,StimC,B,0,25   A 0  1    RT 39
157 Green_Pict,StimB,B,25,0   A 0  0    RT 39
158 Worms_Pict,StimD,B,0,25   B 1  1    RT 65
159 Worms_Pict,StimD,B,0,25   B 1  1    RT 45
160 SticksPict,StimA,A,25,0   B 0  0    RT 39


Total AB Correct = 16 (20% correct)
Total CD Correct = 58 (72.5% correct)
Total AB optimal = 0 (0% optimal)
Total CD optimal = 68 (85% optimal)
Total points = 350


BALANCE Green_Pict B  A 1    RT 31
BALANCE SticksPict A  B 1    RT 137
BALANCE Green_Pict B  A 1    RT 35
BALANCE Green_Pict B  A 1    RT 31
BALANCE Green_Pict B  A 1    RT 44
BALANCE Green_Pict B  A 0    RT 28
BALANCE Green_Pict B  A 1    RT 50
BALANCE SticksPict A  B 1    RT 38
```
