# Supplementary material for: Identifying the Basal Ganglia Network Model Markers for Medication-Induced Impulsivity in Parkinson's Disease Patients
Source: PLoS One. 2015 Jun 4;10(6):e0127542. doi: 10.1371/journal.pone.0127542 (PMC4456385; doi:10.1371/journal.pone.0127542)
Supplement: S4 File — (ZIP) [file pone.0127542.s004.zip › SMat/Quarters_PDoff6.doc]

```
QuartersCF4
Subject ID: 4
 Stimuli:
A = SticksPict B= CirclePict
C = Green_Pict C= Worms_Pict
Stim A is class A 80% of trials; Stim B is class A 20% of trials
    win = 25 points, lose is 0
Stim C is class A 80% of trials; Stim D is class A 20% of trials
    win = 0, lose = 25 points


Key: A/B is subj response, 0/1 is correct?, 0/1 is optimal?
1 SticksPict,StimA,B,25,0   B 1  0    RT 63
2 Worms_Pict,StimD,B,0,25   A 0  0    RT 44
3 Green_Pict,StimC,B,0,25   A 0  1    RT 51
4 SticksPict,StimA,A,25,0   B 0  0    RT 52
5 Green_Pict,StimC,A,0,25   B 0  0    RT 52
6 Green_Pict,StimC,A,0,25   A 1  1    RT 32
7 CirclePict,StimB,B,25,0   A 0  0    RT 49
8 Green_Pict,StimC,A,0,25   A 1  1    RT 25
9 CirclePict,StimB,B,25,0   B 1  1    RT 58
10 Worms_Pict,StimD,B,0,25   A 0  0    RT 52
11 Green_Pict,StimC,A,0,25   A 1  1    RT 85
12 SticksPict,StimA,B,25,0   B 1  0    RT 58
13 SticksPict,StimA,A,25,0   B 0  0    RT 39
14 Worms_Pict,StimD,B,0,25   B 1  1    RT 57
15 SticksPict,StimA,A,25,0   A 1  1    RT 51
16 CirclePict,StimB,A,25,0   B 0  1    RT 53
17 Worms_Pict,StimD,B,0,25   A 0  0    RT 67
18 CirclePict,StimB,B,25,0   B 1  1    RT 53
19 Green_Pict,StimC,A,0,25   B 0  0    RT 53
20 CirclePict,StimB,B,25,0   B 1  1    RT 61
21 SticksPict,StimA,A,25,0   B 0  0    RT 48
22 CirclePict,StimB,A,25,0   A 1  0    RT 41
23 CirclePict,StimB,B,25,0   A 0  0    RT 26
24 CirclePict,StimB,B,25,0   A 0  0    RT 22
25 CirclePict,StimB,B,25,0   A 0  0    RT 23
26 Worms_Pict,StimD,B,0,25   B 1  1    RT 87
27 SticksPict,StimA,A,25,0   B 0  0    RT 41
28 Worms_Pict,StimD,A,0,25   A 1  0    RT 64
29 SticksPict,StimA,A,25,0   B 0  0    RT 39
30 Green_Pict,StimC,A,0,25   A 1  1    RT 24
31 Worms_Pict,StimD,B,0,25   B 1  1    RT 31
32 Worms_Pict,StimD,B,0,25   B 1  1    RT 40
33 Worms_Pict,StimD,A,0,25   B 0  1    RT 26
34 Green_Pict,StimC,A,0,25   A 1  1    RT 29
35 Green_Pict,StimC,B,0,25   A 0  1    RT 58
36 SticksPict,StimA,A,25,0   B 0  0    RT 42
37 Green_Pict,StimC,A,0,25   A 1  1    RT 59
38 Worms_Pict,StimD,B,0,25   B 1  1    RT 39
39 CirclePict,StimB,B,25,0   A 0  0    RT 43
40 SticksPict,StimA,A,25,0   B 0  0    RT 33
41 Worms_Pict,StimD,A,0,25   B 0  1    RT 54
42 SticksPict,StimA,A,25,0   B 0  0    RT 51
43 Green_Pict,StimC,A,0,25   A 1  1    RT 30
44 Worms_Pict,StimD,B,0,25   B 1  1    RT 42
45 Worms_Pict,StimD,A,0,25   B 0  1    RT 34
46 Green_Pict,StimC,A,0,25   A 1  1    RT 40
47 SticksPict,StimA,B,25,0   B 1  0    RT 48
48 Green_Pict,StimC,B,0,25   A 0  1    RT 73
49 CirclePict,StimB,B,25,0   B 1  1    RT 94
50 Worms_Pict,StimD,B,0,25   B 1  1    RT 63
51 Worms_Pict,StimD,B,0,25   B 1  1    RT 71
52 SticksPict,StimA,A,25,0   B 0  0    RT 40
53 SticksPict,StimA,A,25,0   B 0  0    RT 40
54 CirclePict,StimB,B,25,0   A 0  0    RT 61
55 SticksPict,StimA,A,25,0   B 0  0    RT 55
56 Green_Pict,StimC,B,0,25   A 0  1    RT 125
57 Green_Pict,StimC,A,0,25   B 0  0    RT 39
58 Worms_Pict,StimD,B,0,25   B 1  1    RT 37
59 CirclePict,StimB,B,25,0   B 1  1    RT 45
60 Green_Pict,StimC,A,0,25   A 1  1    RT 43
61 SticksPict,StimA,A,25,0   B 0  0    RT 36
62 CirclePict,StimB,B,25,0   A 0  0    RT 38
63 CirclePict,StimB,B,25,0   A 0  0    RT 535
64 CirclePict,StimB,B,25,0   A 0  0    RT 28
65 SticksPict,StimA,B,25,0   B 1  0    RT 37
66 Green_Pict,StimC,A,0,25   A 1  1    RT 41
67 Worms_Pict,StimD,B,0,25   B 1  1    RT 39
68 CirclePict,StimB,A,25,0   A 1  0    RT 37
69 CirclePict,StimB,B,25,0   A 0  0    RT 25
70 Worms_Pict,StimD,B,0,25   B 1  1    RT 42
71 Green_Pict,StimC,A,0,25   A 1  1    RT 44
72 SticksPict,StimA,A,25,0   B 0  0    RT 41
73 Green_Pict,StimC,A,0,25   A 1  1    RT 139
74 SticksPict,StimA,A,25,0   B 0  0    RT 2973
75 Worms_Pict,StimD,B,0,25   B 1  1    RT 86
76 Worms_Pict,StimD,B,0,25   B 1  1    RT 26
77 CirclePict,StimB,B,25,0   A 0  0    RT 31
78 Green_Pict,StimC,A,0,25   A 1  1    RT 49
79 CirclePict,StimB,A,25,0   A 1  0    RT 69
80 SticksPict,StimA,A,25,0   B 0  0    RT 38
81 CirclePict,StimB,B,25,0   A 0  0    RT 31
82 CirclePict,StimB,A,25,0   A 1  0    RT 22
83 SticksPict,StimA,A,25,0   B 0  0    RT 39
84 SticksPict,StimA,B,25,0   B 1  0    RT 320
85 Worms_Pict,StimD,B,0,25   B 1  1    RT 77
86 Green_Pict,StimC,A,0,25   A 1  1    RT 39
87 SticksPict,StimA,A,25,0   B 0  0    RT 32
88 Worms_Pict,StimD,B,0,25   B 1  1    RT 45
89 Worms_Pict,StimD,B,0,25   B 1  1    RT 31
90 SticksPict,StimA,A,25,0   B 0  0    RT 39
91 Green_Pict,StimC,A,0,25   A 1  1    RT 35
92 Green_Pict,StimC,B,0,25   A 0  1    RT 30
93 Green_Pict,StimC,A,0,25   A 1  1    RT 25
94 Worms_Pict,StimD,B,0,25   B 1  1    RT 38
95 CirclePict,StimB,B,25,0   A 0  0    RT 56
96 CirclePict,StimB,A,25,0   A 1  0    RT 41
97 SticksPict,StimA,A,25,0   B 0  0    RT 37
98 SticksPict,StimA,A,25,0   B 0  0    RT 36
99 Green_Pict,StimC,A,0,25   A 1  1    RT 34
100 Worms_Pict,StimD,B,0,25   B 1  1    RT 43
101 SticksPict,StimA,A,25,0   B 0  0    RT 39
102 Green_Pict,StimC,A,0,25   A 1  1    RT 46
103 Green_Pict,StimC,A,0,25   A 1  1    RT 61
104 SticksPict,StimA,B,25,0   B 1  0    RT 40
105 Green_Pict,StimC,A,0,25   A 1  1    RT 40
106 Green_Pict,StimC,A,0,25   A 1  1    RT 23
107 CirclePict,StimB,B,25,0   A 0  0    RT 44
108 Worms_Pict,StimD,A,0,25   B 0  1    RT 42
109 SticksPict,StimA,A,25,0   B 0  0    RT 42
110 CirclePict,StimB,B,25,0   A 0  0    RT 34
111 Worms_Pict,StimD,B,0,25   B 1  1    RT 38
112 CirclePict,StimB,B,25,0   A 0  0    RT 38
113 SticksPict,StimA,A,25,0   B 0  0    RT 33
114 Green_Pict,StimC,B,0,25   A 0  1    RT 32
115 CirclePict,StimB,B,25,0   A 0  0    RT 33
116 Worms_Pict,StimD,A,0,25   B 0  1    RT 31
117 CirclePict,StimB,B,25,0   A 0  0    RT 31
118 Worms_Pict,StimD,B,0,25   B 1  1    RT 28
119 CirclePict,StimB,B,25,0   A 0  0    RT 34
120 Worms_Pict,StimD,B,0,25   B 1  1    RT 34
121 CirclePict,StimB,B,25,0   A 0  0    RT 52
122 Worms_Pict,StimD,B,0,25   B 1  1    RT 41
123 SticksPict,StimA,A,25,0   B 0  0    RT 36
124 SticksPict,StimA,A,25,0   B 0  0    RT 30
125 SticksPict,StimA,A,25,0   B 0  0    RT 26
126 CirclePict,StimB,B,25,0   A 0  0    RT 31
127 CirclePict,StimB,B,25,0   A 0  0    RT 23
128 SticksPict,StimA,A,25,0   B 0  0    RT 34
129 Green_Pict,StimC,A,0,25   A 1  1    RT 34
130 Green_Pict,StimC,A,0,25   A 1  1    RT 25
131 Green_Pict,StimC,A,0,25   A 1  1    RT 24
132 Worms_Pict,StimD,B,0,25   B 1  1    RT 59
133 SticksPict,StimA,B,25,0   B 1  0    RT 39
134 Green_Pict,StimC,A,0,25   A 1  1    RT 34
135 Green_Pict,StimC,A,0,25   A 1  1    RT 24
136 Worms_Pict,StimD,B,0,25   B 1  1    RT 30
137 SticksPict,StimA,A,25,0   B 0  0    RT 39
138 CirclePict,StimB,B,25,0   A 0  0    RT 39
139 SticksPict,StimA,A,25,0   B 0  0    RT 34
140 SticksPict,StimA,A,25,0   B 0  0    RT 39
141 Worms_Pict,StimD,B,0,25   B 1  1    RT 41
142 Green_Pict,StimC,B,0,25   A 0  1    RT 30
143 CirclePict,StimB,B,25,0   A 0  0    RT 43
144 CirclePict,StimB,B,25,0   A 0  0    RT 23
145 CirclePict,StimB,A,25,0   A 1  0    RT 21
146 Green_Pict,StimC,B,0,25   A 0  1    RT 34
147 CirclePict,StimB,B,25,0   B 1  1    RT 26
148 SticksPict,StimA,A,25,0   B 0  0    RT 36
149 Worms_Pict,StimD,B,0,25   B 1  1    RT 63
150 Green_Pict,StimC,A,0,25   A 1  1    RT 34
151 Worms_Pict,StimD,B,0,25   B 1  1    RT 42
152 CirclePict,StimB,B,25,0   A 0  0    RT 54
153 CirclePict,StimB,A,25,0   A 1  0    RT 112
154 SticksPict,StimA,B,25,0   B 1  0    RT 32
155 Green_Pict,StimC,A,0,25   A 1  1    RT 30
156 Green_Pict,StimC,A,0,25   A 1  1    RT 29
157 Worms_Pict,StimD,A,0,25   B 0  1    RT 35
158 Worms_Pict,StimD,B,0,25   B 1  1    RT 33
159 Worms_Pict,StimD,A,0,25   B 0  1    RT 29
160 Worms_Pict,StimD,B,0,25   A 0  0    RT 37


Total AB Correct = 22 (27.5% correct)
Total CD Correct = 58 (72.5% correct)
Total AB optimal = 8 (10% optimal)
Total CD optimal = 72 (90% optimal)
Total points = 500


BALANCE SticksPict A  B 0    RT 57
BALANCE CirclePict B  A 1    RT 35
```
