# Supplementary material for: Identifying the Basal Ganglia Network Model Markers for Medication-Induced Impulsivity in Parkinson's Disease Patients
Source: PLoS One. 2015 Jun 4;10(6):e0127542. doi: 10.1371/journal.pone.0127542 (PMC4456385; doi:10.1371/journal.pone.0127542)
Supplement: S4 File — (ZIP) [file pone.0127542.s004.zip › SMat/Quarters_PDoff7.doc]

```
QuartersCF4
Subject ID: Off 7
 Stimuli:
A = Green_Pict B= Worms_Pict
C = CirclePict C= SticksPict
Stim A is class A 80% of trials; Stim B is class A 20% of trials
    win = 25 points, lose is 0
Stim C is class A 80% of trials; Stim D is class A 20% of trials
    win = 0, lose = 25 points


Key: A/B is subj response, 0/1 is correct?, 0/1 is optimal?
1 SticksPict,StimD,B,0,25   B 1  1    RT 124
2 SticksPict,StimD,B,0,25   B 1  1    RT 43
3 SticksPict,StimD,B,0,25   B 1  1    RT 31
4 Green_Pict,StimA,B,25,0   B 1  0    RT 115
5 Green_Pict,StimA,A,25,0   B 0  0    RT 43
6 Worms_Pict,StimB,B,25,0   A 0  0    RT 200
7 Worms_Pict,StimB,B,25,0   A 0  0    RT 81
8 Worms_Pict,StimB,A,25,0   A 1  0    RT 31
9 Worms_Pict,StimB,B,25,0   A 0  0    RT 105
10 Green_Pict,StimA,A,25,0   B 0  0    RT 29
11 Green_Pict,StimA,A,25,0   B 0  0    RT 51
12 SticksPict,StimD,B,0,25   A 0  0    RT 286
13 CirclePict,StimC,A,0,25   A 1  1    RT 208
14 CirclePict,StimC,B,0,25   A 0  1    RT 33
15 Worms_Pict,StimB,B,25,0   B 1  1    RT 68
16 Worms_Pict,StimB,B,25,0   B 1  1    RT 43
17 Worms_Pict,StimB,B,25,0   B 1  1    RT 31
18 SticksPict,StimD,B,0,25   A 0  0    RT 51
19 Worms_Pict,StimB,A,25,0   B 0  1    RT 37
20 Green_Pict,StimA,B,25,0   B 1  0    RT 77
21 CirclePict,StimC,A,0,25   A 1  1    RT 54
22 Green_Pict,StimA,A,25,0   B 0  0    RT 33
23 Green_Pict,StimA,A,25,0   B 0  0    RT 23
24 CirclePict,StimC,A,0,25   A 1  1    RT 57
25 Green_Pict,StimA,A,25,0   B 0  0    RT 135
26 Green_Pict,StimA,A,25,0   B 0  0    RT 26
27 SticksPict,StimD,A,0,25   A 1  0    RT 85
28 CirclePict,StimC,A,0,25   B 0  0    RT 48
29 CirclePict,StimC,B,0,25   A 0  1    RT 24
30 Worms_Pict,StimB,B,25,0   B 1  1    RT 36
31 SticksPict,StimD,B,0,25   A 0  0    RT 107
32 CirclePict,StimC,A,0,25   A 1  1    RT 51
33 SticksPict,StimD,B,0,25   B 1  1    RT 27
34 CirclePict,StimC,A,0,25   A 1  1    RT 60
35 CirclePict,StimC,A,0,25   A 1  1    RT 30
36 SticksPict,StimD,B,0,25   B 1  1    RT 36
37 SticksPict,StimD,A,0,25   B 0  1    RT 27
38 Green_Pict,StimA,A,25,0   B 0  0    RT 61
39 Worms_Pict,StimB,B,25,0   A 0  0    RT 52
40 CirclePict,StimC,A,0,25   B 0  0    RT 52
41 SticksPict,StimD,B,0,25   B 1  1    RT 35
42 SticksPict,StimD,B,0,25   B 1  1    RT 38
43 SticksPict,StimD,B,0,25   B 1  1    RT 27
44 SticksPict,StimD,A,0,25   B 0  1    RT 24
45 Green_Pict,StimA,A,25,0   B 0  0    RT 70
46 Green_Pict,StimA,B,25,0   B 1  0    RT 26
47 CirclePict,StimC,A,0,25   A 1  1    RT 38
48 Green_Pict,StimA,A,25,0   B 0  0    RT 44
49 CirclePict,StimC,A,0,25   A 1  1    RT 46
50 Worms_Pict,StimB,B,25,0   B 1  1    RT 44
51 Green_Pict,StimA,A,25,0   A 1  1    RT 71
52 Worms_Pict,StimB,B,25,0   B 1  1    RT 33
53 Green_Pict,StimA,A,25,0   A 1  1    RT 33
54 Worms_Pict,StimB,B,25,0   B 1  1    RT 33
55 Worms_Pict,StimB,B,25,0   B 1  1    RT 43
56 CirclePict,StimC,A,0,25   A 1  1    RT 48
57 SticksPict,StimD,B,0,25   B 1  1    RT 69
58 Green_Pict,StimA,A,25,0   B 0  0    RT 78
59 Green_Pict,StimA,B,25,0   B 1  0    RT 29
60 SticksPict,StimD,B,0,25   A 0  0    RT 193
61 Worms_Pict,StimB,B,25,0   A 0  0    RT 90
62 Worms_Pict,StimB,B,25,0   A 0  0    RT 53
63 Green_Pict,StimA,A,25,0   B 0  0    RT 50
64 CirclePict,StimC,B,0,25   A 0  1    RT 53
65 Worms_Pict,StimB,B,25,0   A 0  0    RT 70
66 SticksPict,StimD,B,0,25   B 1  1    RT 47
67 Worms_Pict,StimB,A,25,0   A 1  0    RT 53
68 CirclePict,StimC,A,0,25   A 1  1    RT 40
69 Green_Pict,StimA,A,25,0   B 0  0    RT 49
70 CirclePict,StimC,A,0,25   A 1  1    RT 45
71 CirclePict,StimC,A,0,25   A 1  1    RT 25
72 CirclePict,StimC,A,0,25   A 1  1    RT 29
73 SticksPict,StimD,A,0,25   B 0  1    RT 44
74 SticksPict,StimD,B,0,25   B 1  1    RT 37
75 SticksPict,StimD,B,0,25   B 1  1    RT 21
76 Worms_Pict,StimB,A,25,0   A 1  0    RT 119
77 Worms_Pict,StimB,B,25,0   A 0  0    RT 26
78 CirclePict,StimC,A,0,25   A 1  1    RT 46
79 CirclePict,StimC,B,0,25   A 0  1    RT 25
80 Green_Pict,StimA,A,25,0   B 0  0    RT 57
81 Green_Pict,StimA,A,25,0   B 0  0    RT 27
82 SticksPict,StimD,A,0,25   B 0  1    RT 196
83 SticksPict,StimD,B,0,25   B 1  1    RT 23
84 Green_Pict,StimA,A,25,0   B 0  0    RT 63
85 Worms_Pict,StimB,B,25,0   A 0  0    RT 45
86 SticksPict,StimD,B,0,25   B 1  1    RT 283
87 Green_Pict,StimA,A,25,0   B 0  0    RT 40
88 Green_Pict,StimA,B,25,0   B 1  0    RT 34
89 CirclePict,StimC,A,0,25   A 1  1    RT 48
90 Green_Pict,StimA,A,25,0   B 0  0    RT 40
91 Green_Pict,StimA,A,25,0   B 0  0    RT 28
92 SticksPict,StimD,B,0,25   B 1  1    RT 138
93 CirclePict,StimC,B,0,25   A 0  1    RT 68
94 Worms_Pict,StimB,A,25,0   A 1  0    RT 235
95 CirclePict,StimC,B,0,25   B 1  0    RT 57
96 CirclePict,StimC,A,0,25   B 0  0    RT 36
97 Green_Pict,StimA,B,25,0   B 1  0    RT 45
98 Worms_Pict,StimB,B,25,0   A 0  0    RT 40
99 Worms_Pict,StimB,B,25,0   A 0  0    RT 27
100 Worms_Pict,StimB,A,25,0   A 1  0    RT 25
101 CirclePict,StimC,A,0,25   A 1  1    RT 68
102 Worms_Pict,StimB,B,25,0   A 0  0    RT 38
103 Green_Pict,StimA,A,25,0   B 0  0    RT 37
104 SticksPict,StimD,B,0,25   B 1  1    RT 126
105 SticksPict,StimD,B,0,25   B 1  1    RT 32
106 Worms_Pict,StimB,B,25,0   A 0  0    RT 48
107 CirclePict,StimC,A,0,25   A 1  1    RT 51
108 Green_Pict,StimA,A,25,0   B 0  0    RT 37
109 CirclePict,StimC,A,0,25   A 1  1    RT 49
110 CirclePict,StimC,A,0,25   A 1  1    RT 34
111 CirclePict,StimC,A,0,25   A 1  1    RT 30
112 SticksPict,StimD,A,0,25   B 0  1    RT 36
113 SticksPict,StimD,B,0,25   A 0  0    RT 59
114 Worms_Pict,StimB,B,25,0   A 0  0    RT 45
115 Worms_Pict,StimB,B,25,0   A 0  0    RT 65
116 Green_Pict,StimA,A,25,0   B 0  0    RT 49
117 SticksPict,StimD,B,0,25   A 0  0    RT 331
118 CirclePict,StimC,A,0,25   A 1  1    RT 43
119 SticksPict,StimD,B,0,25   B 1  1    RT 33
120 Worms_Pict,StimB,B,25,0   A 0  0    RT 39
121 CirclePict,StimC,B,0,25   A 0  1    RT 47
122 Green_Pict,StimA,B,25,0   B 1  0    RT 34
123 CirclePict,StimC,A,0,25   A 1  1    RT 81
124 Green_Pict,StimA,A,25,0   B 0  0    RT 41
125 Green_Pict,StimA,A,25,0   B 0  0    RT 40
126 CirclePict,StimC,A,0,25   A 1  1    RT 42
127 CirclePict,StimC,A,0,25   A 1  1    RT 34
128 SticksPict,StimD,B,0,25   B 1  1    RT 54
129 Worms_Pict,StimB,B,25,0   A 0  0    RT 51
130 SticksPict,StimD,A,0,25   B 0  1    RT 42
131 SticksPict,StimD,B,0,25   B 1  1    RT 29
132 CirclePict,StimC,A,0,25   A 1  1    RT 41
133 SticksPict,StimD,B,0,25   B 1  1    RT 35
134 Green_Pict,StimA,B,25,0   B 1  0    RT 54
135 Worms_Pict,StimB,B,25,0   A 0  0    RT 43
136 Worms_Pict,StimB,B,25,0   A 0  0    RT 68
137 Green_Pict,StimA,A,25,0   B 0  0    RT 38
138 SticksPict,StimD,B,0,25   B 1  1    RT 53
139 SticksPict,StimD,B,0,25   B 1  1    RT 25
140 CirclePict,StimC,A,0,25   A 1  1    RT 35
141 CirclePict,StimC,A,0,25   A 1  1    RT 61
142 Worms_Pict,StimB,A,25,0   A 1  0    RT 185
143 Green_Pict,StimA,A,25,0   B 0  0    RT 36
144 Green_Pict,StimA,A,25,0   B 0  0    RT 36
145 Green_Pict,StimA,A,25,0   B 0  0    RT 22
146 SticksPict,StimD,B,0,25   B 1  1    RT 131
147 Worms_Pict,StimB,B,25,0   A 0  0    RT 40
148 Worms_Pict,StimB,B,25,0   A 0  0    RT 44
149 Worms_Pict,StimB,B,25,0   A 0  0    RT 28
150 SticksPict,StimD,B,0,25   B 1  1    RT 33
151 Green_Pict,StimA,A,25,0   B 0  0    RT 43
152 SticksPict,StimD,A,0,25   B 0  1    RT 29
153 Worms_Pict,StimB,B,25,0   A 0  0    RT 48
154 SticksPict,StimD,B,0,25   B 1  1    RT 32
155 Green_Pict,StimA,A,25,0   B 0  0    RT 41
156 CirclePict,StimC,A,0,25   A 1  1    RT 69
157 Worms_Pict,StimB,B,25,0   A 0  0    RT 45
158 Worms_Pict,StimB,A,25,0   A 1  0    RT 32
159 CirclePict,StimC,B,0,25   A 0  1    RT 32
160 CirclePict,StimC,A,0,25   A 1  1    RT 24


Total AB Correct = 25 (31.25% correct)
Total CD Correct = 57 (71.25% correct)
Total AB optimal = 11 (13.75% optimal)
Total CD optimal = 69 (86.25% optimal)
Total points = 550
```
