# Supplementary material for: Identifying the Basal Ganglia Network Model Markers for Medication-Induced Impulsivity in Parkinson's Disease Patients
Source: PLoS One. 2015 Jun 4;10(6):e0127542. doi: 10.1371/journal.pone.0127542 (PMC4456385; doi:10.1371/journal.pone.0127542)
Supplement: S4 File — (ZIP) [file pone.0127542.s004.zip › SMat/Quarters_PDoff8.doc]

```
QuartersCF4
Subject ID: Off med 8
Stimuli:
A = CirclePict B= Green_Pict
C = Worms_Pict C= SticksPict
Stim A is class A 80% of trials; Stim B is class A 20% of trials
    win = 25 points, lose is 0
Stim C is class A 80% of trials; Stim D is class A 20% of trials
    win = 0, lose = 25 points


Key: A/B is subj response, 0/1 is correct?, 0/1 is optimal?
1 CirclePict,StimA,A,25,0   A 1  1    RT 281
2 Green_Pict,StimB,B,25,0   B 1  1    RT 396
3 Green_Pict,StimB,B,25,0   B 1  1    RT 225
4 Green_Pict,StimB,A,25,0   B 0  1    RT 121
5 CirclePict,StimA,A,25,0   A 1  1    RT 109
6 Green_Pict,StimB,B,25,0   A 0  0    RT 102
7 CirclePict,StimA,B,25,0   B 1  0    RT 84
8 CirclePict,StimA,A,25,0   A 1  1    RT 88
9 CirclePict,StimA,A,25,0   B 0  0    RT 84
10 CirclePict,StimA,A,25,0   A 1  1    RT 171
11 Green_Pict,StimB,B,25,0   A 0  0    RT 93
12 Green_Pict,StimB,B,25,0   B 1  1    RT 126
13 Green_Pict,StimB,B,25,0   A 0  0    RT 101
14 CirclePict,StimA,A,25,0   B 0  0    RT 71
15 CirclePict,StimA,B,25,0   A 0  1    RT 68
16 Green_Pict,StimB,B,25,0   A 0  0    RT 70
17 Green_Pict,StimB,A,25,0   B 0  1    RT 39
18 Green_Pict,StimB,B,25,0   A 0  0    RT 55
19 CirclePict,StimA,A,25,0   A 1  1    RT 32
20 CirclePict,StimA,A,25,0   A 1  1    RT 63
21 CirclePict,StimA,A,25,0   B 0  0    RT 72
22 CirclePict,StimA,A,25,0   A 1  1    RT 49
23 CirclePict,StimA,A,25,0   A 1  1    RT 42
24 CirclePict,StimA,A,25,0   A 1  1    RT 47
25 CirclePict,StimA,B,25,0   A 0  1    RT 30
26 CirclePict,StimA,A,25,0   B 0  0    RT 59
27 CirclePict,StimA,A,25,0   A 1  1    RT 47
28 Green_Pict,StimB,B,25,0   A 0  0    RT 60
29 Green_Pict,StimB,B,25,0   B 1  1    RT 90
30 CirclePict,StimA,A,25,0   A 1  1    RT 130
31 Green_Pict,StimB,B,25,0   B 1  1    RT 324
32 CirclePict,StimA,A,25,0   A 1  1    RT 87
33 Green_Pict,StimB,A,25,0   B 0  1    RT 81
34 Green_Pict,StimB,B,25,0   A 0  0    RT 75
35 CirclePict,StimA,B,25,0   A 0  1    RT 58
36 Green_Pict,StimB,B,25,0   B 1  1    RT 165
37 Green_Pict,StimB,B,25,0   B 1  1    RT 25
38 Green_Pict,StimB,B,25,0   B 1  1    RT 45
39 Green_Pict,StimB,B,25,0   A 0  0    RT 126
40 Green_Pict,StimB,A,25,0   B 0  1    RT 57
41 CirclePict,StimA,A,25,0   A 1  1    RT 47
42 CirclePict,StimA,B,25,0   A 0  1    RT 32
43 Green_Pict,StimB,A,25,0   B 0  1    RT 65
44 CirclePict,StimA,A,25,0   A 1  1    RT 45
45 Green_Pict,StimB,B,25,0   A 0  0    RT 118
46 CirclePict,StimA,A,25,0   A 1  1    RT 103
47 CirclePict,StimA,A,25,0   A 1  1    RT 28
48 Green_Pict,StimB,B,25,0   B 1  1    RT 52
49 CirclePict,StimA,A,25,0   A 1  1    RT 90
50 CirclePict,StimA,B,25,0   A 0  1    RT 57
51 Green_Pict,StimB,B,25,0   B 1  1    RT 67
52 Green_Pict,StimB,B,25,0   B 1  1    RT 25
53 CirclePict,StimA,A,25,0   A 1  1    RT 91
54 Green_Pict,StimB,B,25,0   B 1  1    RT 53
55 Green_Pict,StimB,A,25,0   A 1  0    RT 51
56 CirclePict,StimA,A,25,0   A 1  1    RT 95
57 Green_Pict,StimB,B,25,0   B 1  1    RT 67
58 Green_Pict,StimB,B,25,0   A 0  0    RT 53
59 Green_Pict,StimB,B,25,0   B 1  1    RT 34
60 CirclePict,StimA,A,25,0   A 1  1    RT 32
61 Green_Pict,StimB,B,25,0   B 1  1    RT 61
62 CirclePict,StimA,A,25,0   A 1  1    RT 175
63 Green_Pict,StimB,A,25,0   B 0  1    RT 51
64 Green_Pict,StimB,A,25,0   B 0  1    RT 105
65 Green_Pict,StimB,B,25,0   A 0  0    RT 25
66 CirclePict,StimA,A,25,0   A 1  1    RT 52
67 CirclePict,StimA,A,25,0   A 1  1    RT 42
68 Green_Pict,StimB,B,25,0   B 1  1    RT 75
69 Green_Pict,StimB,B,25,0   B 1  1    RT 49
70 CirclePict,StimA,A,25,0   A 1  1    RT 51
71 Green_Pict,StimB,B,25,0   A 0  0    RT 27
72 CirclePict,StimA,A,25,0   A 1  1    RT 55
73 Green_Pict,StimB,B,25,0   B 1  1    RT 58
74 CirclePict,StimA,B,25,0   A 0  1    RT 46
75 CirclePict,StimA,A,25,0   B 0  0    RT 73
76 Green_Pict,StimB,B,25,0   B 1  1    RT 245
77 CirclePict,StimA,A,25,0   A 1  1    RT 51
78 Green_Pict,StimB,B,25,0   B 1  1    RT 48
79 CirclePict,StimA,A,25,0   A 1  1    RT 38
80 CirclePict,StimA,B,25,0   A 0  1    RT 85
1 Worms_Pict,StimC,A,0,25   A 1  1    RT 162
```

2 SticksPict,Sti2222222222222222222222222222222222222222222222222222222222222222222222222222222222222222222222222222222222222222222222222222222222222222222222222222222222222222222222222222222222222222222222222222222222222222222222222222222222222222222222222222222222222222222222222222222222222222222222222222222222222222222222222222222222222222222222222222222222222222222222222222222222222222222222222222222222222222222222222222222222222222222222222222222222222222222222222222222222222222222222222222222222222222222222222222222222222222222222222222222222222222222222222222222222222222222222222222222222222222222222222222222222222222222222222222222222222222222222222222222222222222222222222222222222222222222222222222222222222222222222222222222222222222222222222222222222222222222222222222222222222222222222222222222222222222222222222222222222222222222222222222222222222222222222222222222222222222222222222222222222222222222222222222222222222222222222222222222222222333333333333333333333333333333333333333333333333333333333333333333333333333333333333333333333333333333333333333333333333333333333333333333333333333333333333333333333333333333333333333333333333333333333333333333333333333333333333333333333333333333333333333333333333333333333333333333333333333333333333333333333333333333333333333333333333333333333333333333333333333333333333333333333333333333333333333333333333333333333333333333333333333333333333333333333333333333333333333333333333333333333333333333333333333333333333333333333333333333333333333333333333333333333333333333333333333333333333333333333333333333333333333333333333333333333333333333333333333333333333333333333333333333333333333333333333333333333333333333333333333333333333333333333333333333333333333333333333333333333333333333333333333333333333333333333333333333333333333333333333333333333333333333333333333333333333333333333333333333333333333333333333333333333333333333333333333333333333333333333333333333333333333333333333333333333333333333333333333333333333333333333333333333333333333333333333333333333333333333333333333333333333333333333333333333333333333333333333333333333333333333333333333333333333333333333333333333333333333333333333333333333333333333333333333333333333333333333333333333333333333333333333333333333333333333333333333333333333333333333333333333333333333333333333333333333333333333333333333333333333333333333333333333333333333333333333333333333333333333333333333333333333333333333333333333333333333333333333333333333333333333333333333333333333333333333333333333333333333333333333333333333333333333333333333333333333333333333333333333333333333333333333333333333333333333333333333333333333333333333333333333333333333333333333333333333333333333333333333333333333333333333333333333333333333333333333333333333333333333333333333333333333333333333333333333333333333333333333333333333333333333333333333333333333333333333333333333333333333333333333333333333333333333333333333333333333333333333333333333333333333333333333333333333333333333333333333333333333333333333333333333333333333333333333333333333333333333333333333333333333333333333333333333333333333333333333333333333333333333333333333333333333333333333333333333333333333333333333333333333333333333333333333333333333333333333333333333333333333333333333333333333333333333333333333333333333333333333333333333333333333333333333333333333333333333333333333333333333333333333333333333333333333333333333333333333333333333333333333333333333333333333333333333333333333333333333333333333333333333333333333333333333333333333333333333333333333333333333333333333333333333333333333333333333333333333333333333333333333333333333333333333333333333333333333333333333333333333333333333333333333333333333333333333333333333333333
